# Unprecedentedly High Activity and/or High Regio-/Stereoselectivity of Fluorenyl-Based CGC Allyl-Type η^3^:η^1^-*tert*-Butyl(dimethylfluorenylsilyl)amido Ligated Rare Earth Metal Monoalkyl Complexes in Olefin Polymerization

**DOI:** 10.3390/polym11050836

**Published:** 2019-05-08

**Authors:** Ge Guo, Xiaolu Wu, Xiangqian Yan, Li Yan, Xiaofang Li, Shaowen Zhang, Nannan Qiu

**Affiliations:** 1 Key Laboratory of Cluster Science of Ministry of Education, School of Chemistry and Chemical Engineering, Beijing Institute of Technology, 5 South Zhongguancun Street, Haidian District, Beijing 100081, China; gge1993@163.com (G.G.); xiaolustefanie@163.com (X.W.); hsdyxq@126.com (X.Y.); 2 Analytical and Testing Center, Liangxiang Campus of Beijing Institute of Technology, Liangxiang East Road, Fangshan District, Beijing 102488, China; 13581796538@163.com; 3 NHC Key Laboratory of Food Safety Risk Assessment, China National Center for Food Safety Risk Assessment, Beijing 100021, China

**Keywords:** fluorenyl-based CGC allyl-type rare earth metal catalyst, η^3^:η^1^-*tert*-butyl(dimethylfluorenylsilyl)amido ligand, coordination–insertion polymerization, olefins, regio-/stereoselectivity, active species

## Abstract

A series of fluorenyl-based constrained-geometry-configuration (CGC) allyl-type rare earth metal monoalkyl complexes bearing the divalent anionic η^3^:η^1^-*tert*-butyl(dimethylfluorenylsilyl)amido (η^3^:η^1^-FluSiMe_2_N*^t^*Bu) ligand (η^3^:η^1^-FluSiMe_2_N*^t^*Bu)Ln(CH_2_SiMe_3_)(THF)_2_ (**1**–**3**) have been synthesized via the alkane elimination reaction between the FluHSiMe_2_NH*^t^*Bu ligand and rare earth metal tri(trimethylsilylmethyl) complexes Ln(CH_2_SiMe_3_)_3_(THF)_n_. Their structures are characterized by means of NMR spectrum, elemental analyses, and X-ray diffraction. These complexes **1**–**3** are isostructural and isomorphous, and each of them adopts a distorted-trigonal-bipyramidal configuration containing one η^3^:η^1^-FluSiMe_2_N*^t^*Bu ligand, one CH_2_SiMe_3_ ligand, and two THF molecules. Unlike traditional CGC allyl-type rare earth metal complexes showing no or low activity and regio-/stereoselectivity in styrene or MMA polymerization, these complexes **1**–**3** exhibit high catalytic activities and/or high regio-/stereoselectivities in the *cis*-1,4-polymerization of isoprene and myrcene or in the syndiotactic polymerization of styrene under the aid of different activators (borate or borane) and AlR_3_. The in situ ^1^H NMR spectra suggest that the exchanges of chelating ligands such as alkyl groups and divalent anionic η^3^:η^1^-FluSiMe_2_N*^t^*Bu ligands between rare earth metal centers and Al centers result in the formation of a heterobimetallic tetraalkylaluminate complex R_2_Al(*μ*-R)_2_Ln(R)(*μ*-R)_2_AlR_2_, which is activated by activators to form a divalent cationic species [Ln(*μ*-R)_2_AlR_2_]^2+^ as a catalytically active species in the coordination–insertion polymerization of olefins.

## 1. Introduction

**Chart 1:**
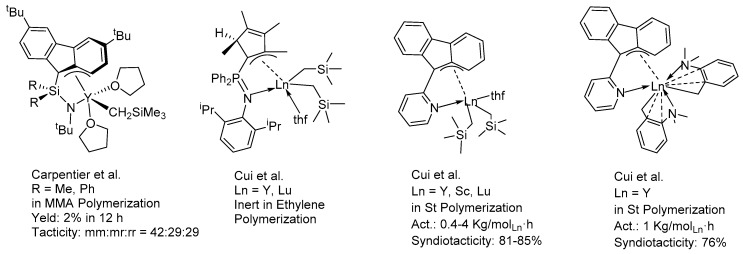
The known constrained-geometry-configuration (CGC) allyl-type rare earth metal catalysts.

The development of highly efficient and highly regio-/stereoselective rare earth metal catalysts has become a hot topic in the coordination–insertion polymerization of olefin over the past two decades, which brings new opportunities for the synthesis of high-performance (co)polymers unavailable from transition metal catalysts [1,2,3,4,5,6,7,8,9,10,11]. So far, a large number of the metallocene [12,13,14,15], constrained-geometry-configuration (CGC) [16,17,18,19], half-sandwich [20,21,22,23], and non-metallocene rare earth metal catalysts precursors bearing different chelating ligands [24,25,26] have emerged for the polymerization of olefins. Among them, the metallocene or nonmetallocene rare earth metal catalysts usually show high activities and different regio-/stereoselectivities in the polymerization of conjugated dienes. The half-sandwich or CGC type rare earth metal catalysts exhibit unprecedentedly high activities and high syndiotactic or isotactic selectivities in the polymerization of styrene. Despite of these results, the CGC allyl-type rare earth metal catalysts containing an η^3^-allyl bonding mode of cyclopentadiene (Cp), indenyl (Ind), or fluorenyl (Flu) moiety are rare [27,28,29], and all of them show no or low activities and regio-/stereoselectivies in olefin polymerization (Chart 1). In 2003, Carpentier et al. reported the synthesis of the amido-functionalized Flu-based CGC allyl-type yttrium monoalkyl complexes [η^3^:η^1^-(3,6-*^t^*Bu_2_Flu)SiR_2_N*^t^*Bu]Y(CH_2_SiMe_3_)(THF)_2_. However, such complexes showed no activities in the ethylene polymerization in a larger range of temperature or very low activities and tacticities in the polymerization of MMA [27]. In 2012, Cui et al. synthesized the phosphazene-functionalized Cp-based CGC allyl-type rare earth metal dialkyl complexes [η^3^:η^1^-(C_5_Me_4_)PPh_2_N(2,6-*^i^*Pr_2_C_6_H_3_)]Y(CH_2_SiMe_3_)_2_(THF), which were inert even in ethylene polymerization [28]. Subsequently, the pyridyl-functionalized Flu-based CGC allyl-type rare earth metal dialkyl complexes (η^3^:η^1^-FluC_5_H_4_N)Ln(CH_2_SiMe_3_)_2_THF and (η^3^:η^1^-FluC_5_H_4_N)Y(CH_2_C_6_H_4_-*o*-NMe_2_)_2_ also developed by Cui and co-workers could promote the polymerization of styrene (ST) but with low activities and moderate syndiotacticities [29]. Therefore, it is of great interest to develop highly efficient and highly regio-/stereoselective CGC allyl-type rare earth metal catalysts and explore their catalytic performances in the polymerization of olefins.

Recently, we have paid much attention to the synthesis of the half-sandwich Flu-ligated rare earth metal dialkyl complexes Flu’Ln(CH_2_SiMe_3_)_2_(THF)_n_ and their applications in the coordination–insertion (co)polymerization of olefins such as ST or conjugated dienes. In 2013, these complexes displayed high activities up to 3.4 × 10^7^ (g of polymer)/(mol_Ln_ h) and syndiotacticities up to >99% in ST polymerization when activated by an activator with or without a small amount of Al*^i^*Bu_3_ [30]. Moreover, such complexes also showed very high activities up to 1.9 × 10^7^ (g of polymer)/(mol_Ln_ h) and high *cis*-1,4-selectivities, 93% in the polymerization of isoprene (IP) in the presence of activator and AlR_3_ [31]. In addition, such catalysts were also active in the regioselective polymerization of 1,3-cyclohexadiene and copolymerization with ST and IP [32]. These results demonstrate that the effective adjustment of the skeleton of the Flu ligand of these complexes has an important impact on their catalytical performance in the olefin polymerization, which arouses our interests to explore more Flu-based rare earth metal complexes and detect their catalytic performance in olefin polymerization. Herein, we report the synthesis and structural characterization of three Flu-based CGC allyl-type rare earth metal monoalkyl complexes (η^3^:η^1^-FluSiMe_2_N*^t^*Bu)Ln(CH_2_SiMe_3_)(THF)_2_ **1**–**3** (**1**: Ln = Sc; **2**: Ln = Lu; **3**: Ln = Y) via the alkane elimination reaction between the *tert*-butyl(dimethylfluorenylsilyl)amido (FluHSiMe_2_NH*^t^*Bu) ligand and the rare earth metal trialkyl complexes Ln(CH_2_SiMe_3_)_3_(THF)_n_. Activated by different cocatalysts, these complexes **1**–**3** unprecedentedly exhibit high activities and high regio-/stereoselectivities in the polymerization of IP, myrcene (MY), or ST, affording the *cis*-1,4-poly(conjugated diene)s or syndiotactic polystyrenes with high molecular weights and moderate molecular weight distributions. The possible coordination–insertion polymerization mechanism is investigated by means of the in situ ^1^H NMR spectrum.

## 2. Materials and Methods

### 2.1. Materials

All manipulations that were sensitive to air or moisture were performed in a MBraun glovebox (Munich, Germany). Activator borate and borane were bought from Tosoh Finechem Corporation (Tokyo, Japan). LiCH_2_SiMe_3_ (1.0 M solution in pentane) and LnCl_3_ (Ln = Sc, Y, Lu; 99.9% analytically pure) were bought from Aldrich (St. Louis, MO, USA). Al*^i^*Bu_3_ (1.0 M solution in hexane), AlMe_3_ (1.0 M solution in toluene), AlEt_3_ (1.0 M solution in heptane), fluorine (99% analytically pure), *tert*-butylamine (98% analytically pure), triethylamine (analytically pure), dichlorodimethylsilane (98% analytically pure), *n*-BuLi (2.4 M in hexane), Na_2_SO_4_ (analytically pure), CaH_2_ (98% analytically pure), dichloromethane (analytically pure), petroleum ether (analytically pure), and methanol (analytically pure) were obtained from Energy Chemistry (Beijing, China). The FluHSiMe_2_NH*^t^*Bu ligand was prepared according to the literature (Supplementary Materials) [33]. IP, MY and ST (analytically pure) were purchased from Aldrich and TCI (Tokyo, Japan). Toluene (Tol), THF, and hexane were purified by a solvent purification system (SPS-800, Mbraun, Garching, Germany), and dried over Na in the glovebox. Chlorobenzene (PhCl), ortho-dichlorobenzene (PhCl_2_), and 1,1,2,2-tetrachloroethane (C_2_H_2_Cl_4_) were dried over CaH_2_ under stirring for 48 h and distilled under reduced pressure before use. The deuterated solvents C_6_D_6_ (99.6 atom% D), C_7_D_8_ (99.5 atom% D), and CDCl_3_ (99.8 atom% D) were purchased from Cambridge Isotope (Tewksbury, MA, USA).

### 2.2. Method

By using J. Young valve NMR tubes, the samples of rare earth metal catalysts were prepared for NMR spectroscopic measurements in the glove box. ^1^H, ^13^C NMR spectra of ligand and catalysts were tested on a Bruker AVANCE 400 spectrometer in C_6_D_6_ or C_7_D_8_ at room temperature. ^1^H, ^13^C NMR spectra of polyisoprene (PIP), polymyrcene (PMY) and polystyrene (PST) samples were recorded on a Bruker AVANCE 400 spectrometer in CDCl_3_ at room temperature or at 60 °C. The molecular weights and the molecular weight distributions (PDI) of the poly(conjugated dienes)s were performed at 25 °C by gel permeation chromatography (GPC) on a WATERS 1515 apparatus. THF was selected as the eluent at a flow rate of 1 mL/min. For SPSTs, GPC data were performed in 1,2,4-trichlorobenzene at 150 °C using IR detection and calibration against polystyrene. Differential scanning calorimetry (DSC) measurements were carried out on a TA 60 (TA Co.) at a rate of 10 °C/min. Any thermal history difference in the poly(conjugated diene)s was eliminated by first heating the specimen to 100 °C, cooling at 10 °C/min to –100 °C, and then recording the second DSC scan. For SPSTs, DSC parameter was set to 10 °C/min to speed up to 300 °C, then cooled at 10 °C/min to room temperature, before recording the second DSC scan. Elemental analyses were performed on an Elementary Vario MICRO CUBE (Germany).

### 2.3. X-ray Crystallographic Analysis

The crystals of complexes **1**–**3** were oil sealed under a microscope in the glove box. For data collection at –100 °C, a CCD area detector using graphite-monochromated Mo Kα radiation (λ = 0.71073 Å) was chosen on a Bruker Smart-Apex CCD diffractometer. The SMART program package was used to determine the crystal class and unit cell parameters. SAINT and SADABS were adopted to process the original frame data and generated the reflection data file. Shelxtl-97 program was applied to solve the structure. F2 anisotropic non-hydrogen atoms were refined by using the full matrix least square method. All the non-hydrogen atoms were anisotropy refined, and all hydrogen atoms were introduced in the calculated positions and were included in the structure calculation without further refinement of the parameters. Crystallographic data (excluding structure factors) have been deposited with the Cambridge Crystallographic Data Centre as supplementary publication nos. CCDC-1904924 (**1**), CCDC-1904923 (**2**), and CCDC-1904925 (**3**) containing the supplementary crystallographic data for this paper. These data can be obtained free of charge via www.ccdc.cam.ac.uk/data_request/cif from The Cambridge Crystallographic Data Centre.

### 2.4. Synthesis of (η^3^:η^1^-FluSiMe_2_N^t^Bu)Ln(CH_2_SiMe_3_)(THF)_2_ **1**–**3**

To a colorless hexane solution (15.0 mL) of Ln(CH_2_SiMe_3_)_3_(THF)_2_ **1**–**3** (**1**: Ln = Sc; **2**: Ln = Lu; **3**: Ln = Y. 1.0 mmol) was added a solution of the FluHSiMe_2_NH*^t^*Bu ligand (1.0 mmol) in hexane (15.0 mL) at room temperature. The mixture was stirred at room temperature for 2–3 h. After removal of all volatiles in vacuo, the residue was recrystallized from toluene/hexane at –30 °C to give Flu-based CGC allyl type rare earth metal monoalkyl complexes [η^3^:η^1^-FluSiMe_2_N*^t^*Bu]Ln(CH_2_SiMe_3_)(THF)_2_ **1**–**3** (**1**: Ln = Sc, 63%; **2**: Ln = Lu, 67%; **3**: Ln = Y, 84%).

^1^H NMR of complex **1** (400 MHz, Tol-*d*8): *δ* 8.10 (d, *J* = 7.8 Hz, 2H, Flu), 8.02 (d, *J* = 8.0 Hz, 2H, Flu), 7.41 (t, *J* = 7.3 Hz, 2H, Flu), 7.12 (d, *J* = 7.4 Hz, 2H, Flu), 3.00 (br, 8H, THF-α-CH_2_), 1.50 (s, 9H, NC(CH_3_)_3_), 1.14 (t, 8H, THF-β-CH_2_), 0.78 (s, 6H, Si(CH_3_)_2_), 0.17 (s, 9H, CH_2_Si(CH_3_)_3_), –1.09 (s, 2H, CH_2_Si(CH_3_)_3_). ^13^C NMR of complex **1** (100 MHz, C_6_D_6_): *δ* 142.83, 131.10, 125.80, 120.91, 117.65, 116.96, 84.13 (C1), 69.92 (α-THF), 54.47 (NC(CH_3_)_3_), 36.51 (NC(CH_3_)_3_), 30.59 (d, *J* = 45.4 Hz, ScCH_2_Si(CH_3_)_3_), 25.02 (β-THF), 5.94 (Si(CH_3_)_2_), 4.62 (CH_2_Si(CH_3_)_3_). Anal. Calcd (%) for C_31_H_50_NO_2_ScSi_2_: C, 65.45; H, 8.68; N, 2.46. Found: C, 65.40; H, 8.61; N, 2.53.

^1^H NMR of complex **2** (400 MHz, C_6_D_6_): *δ* 8.21 (d, *J* = 7.7 Hz, 2H, Flu), 8.07 (d, *J* = 7.7 Hz, 2H, Flu), 7.52 (t, *J* = 7.1 Hz, 2H, Flu), 7.22 (t, *J* = 7.2 Hz, 2H, Flu), 2.94 (br, 8H, THF-α-CH_2_), 1.59 (s, 9H, NC(CH_3_)_3_), 1.08 (t, 8H, THF-β-CH_2_), 0.79 (s, 6H, Si(CH_3_)_2_), 0.28 (s, 9H, CH_2_Si(CH_3_)_3_), –0.82 (s, 2H, CH_2_Si(CH_3_)_3_). ^13^C NMR of complex **2** (100 MHz, C_6_D_6_): *δ* 144.20, 132.67, 125.73, 120.34, 117.47, 116.83 (s), 84.02 (C1), 69.77 (α-THF), 54.40 (NC(CH_3_)_3_), 36.22 (NC(CH_3_)_3_), 31.98 (LuCH_2_Si(CH_3_)_3_), 25.22 (β-THF), 5.55 (Si(CH_3_)_2_), 4.76 (CH_2_Si(CH_3_)_3_). Anal. Calcd (%) for C_31_H_50_NO_2_LuSi_2_: C, 53.28; H, 7.07; N, 2.00. Found: C, 53.21; H, 7.01; N, 2.04.

^1^H NMR of complex **3** (400 MHz, Tol-*d*8): *δ* 8.16 (d, *J* = 7.8 Hz, 2H, Flu), 8.09 (d, *J* = 8.1 Hz, 2H, Flu), 7.47 (t, *J* = 7.5 Hz, 2H, Flu), 7.19 (s, 2H, Flu), 3.05 (br, 8H, THF-α-CH_2_), 1.55 (s, 9H, NC(CH_3_)_3_), 1.19 (t, 8H, THF-β-CH_2_), 0.85 (s, 6H, Si(CH_3_)_2_), 0.23 (s, 9H, CH_2_Si(CH_3_)_3_), –1.06 (s, 2H, CH_2_Si(CH_3_)_3_). ^13^C NMR of complex **3** (100 MHz, C_6_D_6_): *δ* 142.51, 131.62, 124.59, 120.16, 117.53, 116.66, 83.57 (C1), 69.75 (α-THF), 53.97 (NC(CH_3_)_3_), 36.41 (NC(CH_3_)_3_), 35.62 (YCH_2_Si(CH_3_)_3_), 24.74 (β-THF), 5.34 (Si(CH_3_)_2_), 4.38 (CH_2_Si(CH_3_)_3_). Anal. Calcd (%) for C_31_H_50_NO_2_YSi_2_: C, 60.76; H, 8.06; N, 2.29. Found: C, 60.72; H, 8.00; N, 2.34.

### 2.5. A Typical Procedure for IP Polymerization in Table 2 Entry 4

In a glovebox at 25 °C, toluene solution (3.5 mL), Al*^i^*Bu_3_ (100 μL, 1.0 M, 100 μmol), complex **1** (0.0057 g, 10 µmol), a toluene solution (1.5 mL) of [Ph_3_C][B(C_6_F_5_)_4_] (0.0093 g, 10 μmol), and IP (0.34 g, 5 mmol) were added into a 50 mL round bottom flask in succession. The reaction system became sticky rapidly. After 2 min, the flask was taken outside and then quenched by addition of ethanol (50 mL, containing 5% butylhydroxytoluene (BHT) as stabilizing agent). The mixture was washed with ethanol and then dried under vacuum at 45 °C to a constant weight (0.34 g, yield = 100%). The resulting polymer was soluble in THF and chloroform at room temperature. The isomer contents of the polyisoprene was calculated from the ^1^H and ^13^C NMR spectra according to the following Formulas (1)–(5):

Mol 1,4-IP% = [I_H1_/(I_H1_ + 0.5I_H2_)] × 100% (1)

Mol 3,4-IP% = [0.5I_H2_/(I_H1_ + 0.5I_H2_)] × 100% (2)

in which I_H1_ represents the resonance integration of the one vinyl proton of the 1,4-isoprene unit at 5.13 ppm in the ^1^H NMR spectrum; and I_H2_ represents the resonance integration of the two vinyl protons of the 3,4-isoprene unit at 4.72 ppm in the ^1^H NMR spectrum.

Mol *cis*-1,4-IP% = [I_C1_/(I_C1_ + I_C2_ + I_C3_)] × 100% (3)

Mol *trans*-1,4-IP% = [I_C3_/(I_C1_ + I_C2_ + I_C3_)] × 100% (4)

Mol 3,4-IP% = [I_C2_/(I_C1_ + I_C2_ + I_C3_)] × 100% (5)

in which I_C1_ is the integration of 23.2 ppm signals of the *cis*-1,4-isoprene unit methyl carbon, and I_C2_ is the integration of 18.5 ppm signals of the 3,4-isoprene unit methyl carbon, while I_C3_ is the integration of 15.9 ppm signals of the *trans*-1,4-isoprene unit methyl carbon in the ^13^C NMR spectrum.

### 2.6. A Typical Procedure for MY Polymerization in Table 3 Entry 3

In a glovebox at 25 °C, toluene solution (3.5 mL), Al*^i^*Bu_3_ (100 μL, 1.0 M, 100 μmol), complex **3** (0.0063 g, 10 μmol), a toluene solution (1.5 mL) of B(C_6_F_5_)_3_ (0.0052 g, 10 μmol), and MY (0.68 g, 5 mmol) were added into a 50 mL round bottom flask in succession. The reaction system became sticky rapidly. After 1 h, the flask was taken outside and then quenched by addition of ethanol (50 mL, containing 5% butylhydroxytoluene (BHT) as stabilizing agent). The mixture was washed with ethanol and then dried under vacuum at 45 °C to a constant weight (0.49 g, yield = 72%). The resulting polymer was soluble in THF and chloroform at room temperature. The isomer contents of the PMY products were calculated from the ^1^H NMR spectra (Formulas (6)–(8) and ^13^C NMR spectra (Formulas (9)–(12).

Mol 1,2-MY% = [I_5.30_/(I_5.11_ + 0.5I_4.76_)] × 100% (6)

Mol 3,4-MY% = [I_4.76_-2I_5.30_/(I_5.11_ + 0.5I_4.76_)] × 100% (7)

Mol 1,4-MY% = [(I_5.11_-0.5I_4.76_)/(I_5.11_ + 0.5I_4.76_)] × 100% (8)

Mol 1,2-MY% = [I_29.07_/(I_29.07_ + I_37.09_ + I_37.51_ + I_42.18_)] × 100% (9)

Mol 3,4-MY% = [I_42.18_/(I_29.07_ + I_37.09_ + I_37.51_ + I_42.18_)] × 100% (10)

Mol *cis*-1,4-MY% = [I_37.09_/(I_29.07_ + I_37.09_ + I_37.51_ + I_42.18_)] × 100% (11)

Mol *trans*-1,4-MY% = [I_37.51_/(I_29.07_ + I_37.09_ + I_37.51_ + I_42.18_)] × 100% (12)

### 2.7.A Typical Procedure for ST Polymerization in Table 4 Entry 9

In a glovebox at 25 °C, toluene solution (3.5 mL), Al*^i^*Bu_3_ (100 μL, 1.0 M, 100 μmol), complex **1** (0.0057 g, 10 μmol), a toluene solution (1.5 mL) of [PhNHMe_2_][B(C_6_F_5_)_4_] (0.0081 g, 10 μmol), and ST (0.52 g, 5 mmol) were added into a 50 mL round bottom flask in succession. Some solids were gradually precipitated from the reaction system. After 20 h, the flask was taken outside and then quenched by addition of ethanol (50 mL, containing 5% butylhydroxytoluene (BHT) as stabilizing agent). The mixture was washed with ethanol and then dried under vacuum at 45 °C to a constant weight (0.22 g, yield = 43%). The resulting polymer is soluble in CHCl_3_ and 1,2,4-trichlorobenzene at high temperature. The isomer contents of the polystyrene products were calculated from the ^1^H and ^13^C NMR spectra according to the following Formulas (13) and (14):

Mol isotactic PST% = [I_C1_/I_C1_] × 100% (13)

Mol syndiotactic PST% = [I_C2_/I_C3_] × 100% (14)

in which I_C1_ is the integration of the resonance at 146.8 ppm (*mmmm*) and I_C2_ is the integration of the resonance at 145.4 ppm (*rrrr*) in the ^13^C NMR spectrum.

## 3. Results and Discussion

### 3.1. Synthesis of Flu-Based CGC Allyl-Type Rare Earth Metal Monoalkyl Complexes 1–3

**Scheme 1:**
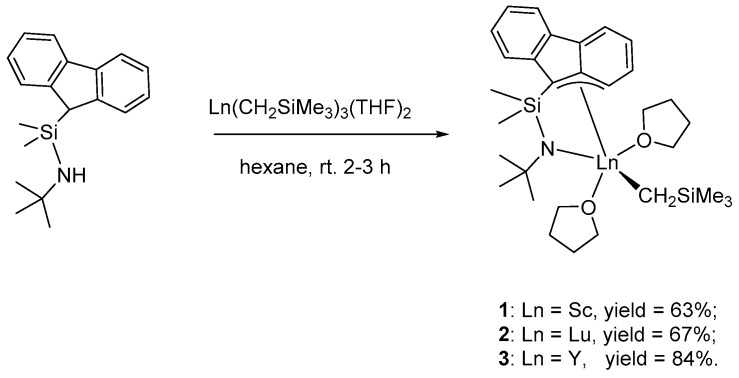
Synthesis of the Flu-based CGC allyl-type rare earth metal monoalkyl complexes **1**–**3**.

The FluSiMe_2_N*^t^*Bu ligand was synthesized according to the literature [33]. The alkane elimination reaction between the FluSiMe_2_N*^t^*Bu ligand and 1 equivalent unit of the rare earth metal tri(trimethylsilylmethyl) complexes Ln(CH_2_SiMe_3_)_3_(THF)_n_ straightforwardly yielded the Flu-based CGC allyl-type rare earth metal monoalkyl complexes (η^3^:η^1^-FluSiMe_2_N*^t^*Bu)Ln(CH_2_SiMe_3_)(THF)_2_ **1**–**3** (**1**: Ln = Sc, 63%; **2**: Ln = Lu, 67%; **3**: Ln = Y, 84%) with moderate to high yields in 2–3 h (Scheme 1). In comparison with the slow synthesis speed of previous similar results [η^3^:η^1^-(3,6-*^t^*Bu_2_Flu)SiR_2_N*^t^*Bu]Y(CH_2_SiMe_3_)(THF)_2_ (37% yield after 1 h and 90% yield after a few days) [27], the rapid synthesis speed of these complexes might be attributed to the lesser bulk of the FluHSiMe_2_NH*^t^*Bu ligand than that of the (3,6-*^t^*Bu_2_-FluH)SiR_2_NH*^t^*Bu ligand.

### 3.2. Structural Characterization of Flu-Based CGC Allyl-Type Rare Earth Metal Monoalkyl Complexes **1**–**3**

These complexes **1**–**3** have good solubilities in common organic solvents such as hexane, toluene and THF. In the ^1^H NMR spectra of the complexes **1**–**3** in C_7_D_8_ and C_6_D_6_, the disappearance of the proton signals attributed to the Flu−H and N−H group of the FluHSiMe_2_NH*^t^*Bu ligand suggests the generation of a dianionic chelating ligand in these complexes. Moreover, the eight Flu protons are divided into four peaks, indicating an asymmetric coordination mode of the Flu ligand around the metal center [29]. In each case, the molar ratio of the integral areas of the signals for the FluSiMe_2_N*^t^*Bu ligand, the CH_2_SiMe_3_ ligand, and THF molecules is 1:1:2. Similar to the flexible [(3,6-*^t^*Bu_2_C_13_H_6_)SiR_2_N*^t^*Bu]Y(CH_2_SiMe_3_)(THF)_2_ at room temperature [27], these complexes also have a flexible structure and the CH_2_SiMe_3_ group in these complexes can not be fixed at the NMR time scale at room temperature since the two methylene protons of the Ln-CH_2_SiMe_3_ groups show only a singlet at high field for **1** at –1.09 ppm, for **2** at –0.82 ppm, and for **3** at –1.06 ppm, respectively.

**Figure 1:**
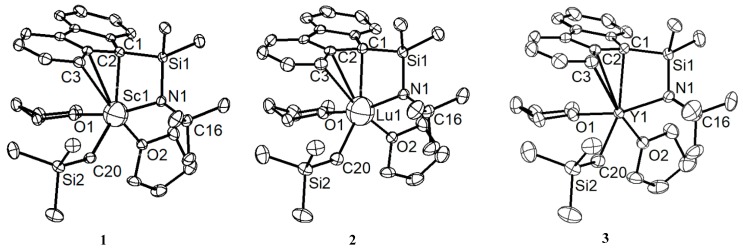
ORTEP (Oak Ridge Thermal-Ellipsoid Plot Program) drawings of complexes **1**–**3** with thermal ellipsoids with a 30% probability. Hydrogen atoms are omitted for clarity.

In a mixed toluene/hexane solution at –30 °C, single crystals of the complexes **1**–**3** were cultivated for an X-ray determination. The ORTEP (Oak Ridge Thermal-Ellipsoid Plot Program) drawings of the complexes **1**–**3** are shown in Figure 1 and the representative bond distances and angles are summarized in Table 1. The X-ray diffraction study reveals that these complexes **1**–**3** are isomorphous and isostructural. Similar to the previous Flu-based CGC allyl-type complex [η^3^:η^1^-(3,6-*^t^*Bu_2_C_13_H_6_)SiR_2_N*^t^*Bu]Y(CH_2_SiMe_3_)(THF)_2_ [27], each of them contains one dianionic FluSiMe_2_N*^t^*Bu ligand, one CH_2_SiMe_3_ group, and two coordinated THF molecules and adopts a distorted-trigonal-bipyramidal configuration. Moreover, the 9-position carbon atom (C1) and the two adjacent carbon atoms (C2, C3) of one phenyl (Ph) ring of the Flu ligand are bound to the metal center in an asymmetric η^3^-allyl mode. The distance of Ln–C1 (2.395(3)–2.566(3) Å) is shorter than those of Ln–C2 (2.730(3)–2.769(3) Å) and Ln–C3 (3.036(4)–3.067(3) Å), implying the more possible presence of –C1–C2=C3 than –C3–C2=C1 in such asymmetric η^3^-Flu ligands. By comparison, the bond distances of Ln–N1, Ln–O1, Ln–O2, Ln–C1 as well as Ln–C2 increase in the order of **1** < **2** < **3,** which are consistent with the trend of the increased ionic radius of the metal centers (Sc < Lu < Y).

### 3.3. Cis-1,4-Polymerization of Ip by the Complexes **1**–**3**/activator/AlR_3_ Ternary Systems

The complexes **1**–**3** alone, the complexes **1**–**3**/AlR_3_ binary systems, and the complexes **1**–**3**/activator binary systems were inactive in IP polymerization. In the presence of both activator and AlR_3_, however, these complexes **1**–**3** unprecedentedly exhibited high activities and regio-/stereoselectivities in IP polymerization under mild conditions as shown by ^1^H and ^13^C NMR analysis (Table 2 and supporting information). At the very beginning, the best catalytic system was investigated for IP polymerization. At first, the Y complex **3** and 2.5 equivalent units of Al*^i^*Bu_3_ were fixed for screening of activators. As an activator, trityl borate [Ph_3_C][B(C_6_F_5_)_4_] (**A**) showed low activity, approximately 4 × 10^3^ (g of polymer)/(mol_Ln_ h) and moderate *cis*-1,4-selectivity of approximately 83% in 30 min. (Table 2, entry 1), while anilinum borate [PhNHMe_2_][B(C_6_F_5_)_4_] (**B**) displayed moderate activity, approximately 18 × 10^3^ (g of polymer)/(mol_Ln_ h) and low *cis*-1,4-selectivity of approximately 65% under the same condition (Table 2, entry 2). By contrast, neutral borane B(C_6_F_5_)_3_ (**C**) was inert for the polymerization of IP even in a long polymerization time (Table 2, entry 3). In order to prepare PIPs with high *cis*-1,4-selectivities, the borate **A** was chosen as an optimum activator in the following IP polymerization. In the presence of borate **A** and 10 equivalent units of Al*^i^*Bu_3_, high activity approximately 1.1 × 10^6^ (g of polymer)/(mol_Ln_ h) and high *cis*-1,4-selectivity of approximately 90% were obtained in IP polymerization catalyzed by the Sc complex **1** only in 2 min, affording main *cis*-1,4-PIP with high molecular weight and moderate molecular weight distribution (*M*_n_ = 700 kg/mol, *M*_w_/*M*_n_ = 2.26) (Table 2, entry 4). In comparison, the Lu complex **2** and the Y complex **3** had moderate activities (1.6 × 10^4^–1.7 × 10^4^ (g of polymer)/(mol_Ln_ h)) and similar or slightly lower *cis*-1,4-selectivities (86%–90%) under the same conditions to prepare main *cis*-1,4-PIPs with lower molecular weights and broader molecular weight distributions (*M*_n_: 100–300 kg/mol, *M*_w_/*M*_n_: 3.17–3.26) (Table 2, entries 5–6). Therefore, the Sc complex **1** served as an optimized catalyst in the following polymerization of IP. 

**Table 2:**
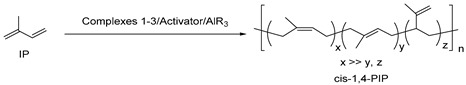
*Cis*-1,4-polymerization of isoprene by complexes **1**–**3**/activator/AlR_3_ ternary systems.*^a^*

entry	Cat.	A*^b^*	AlR_3_				t (h)	T (°C)	Y (%)	A*^c^*	Microstructure (%)*^d^*			*M*_n_^*e*^ (10^5^)	*M*_w_/*M*_n_*^e^*	*T*_g_^*f*^ (°C)
				[Al]/[Ln]	[IP]/[Ln]	Sol.					*c*-1,4	*t*-1,4	3,4			
1	3	A	Al*^i^*Bu_3_	2.5	500	Tol	0.5	25	6	4	83	0	17	1	3.23	−55
2	3	B	Al*^i^*Bu_3_	2.5	500	Tol	0.5	25	26	18	65	23	12	7	2.31	−52
3	3	C	Al*^i^*Bu_3_	2.5	500	Tol	48	25	-	-	-	-	-	-	-	-
4	1	A	Al*^i^*Bu_3_	10	500	Tol	0.03	25	100	1135	90	1	9	7	2.26	−56
5	2	A	Al*^i^*Bu_3_	10	500	Tol	1	25	51	17	90	0	10	3	3.17	−57
6	3	A	Al*^i^*Bu_3_	10	500	Tol	1	25	47	16	86	0	14	1	3.26	−55
7	1	A	AlMe_3_	10	500	Tol	2	25	76	13	94	1	5	2	2.55	−62
8	1	A	AlEt_3_	10	500	Tol	0.08	25	99	421	92	0	8	10	1.78	−59
9	1	A	AlMe_3_	10	500	PhCl	18	25	14	0.3	91	4	5	2	2.58	−57
10	1	A	AlMe_3_	10	500	PhCl_2_	2	25	100	17	94	1	5	5	1.79	−58
11	1	A	AlMe_3_	5	500	PhCl_2_	2	25	100	17	94	1	5	6	2.33	−60
12	1	A	AlMe_3_	20	500	PhCl_2_	2	25	76	13	94	1	5	4	2.67	−58
13	1	A	AlMe_3_	10	500	PhCl_2_	5	–10	50	3	96	0	4	10	1.80	−66
14	1	A	AlMe_3_	10	500	PhCl_2_	2	0	44	7	95	1	4	7	2.10	−62
15	1	A	AlMe_3_	10	500	PhCl_2_	0.5	50	100	68	93	1	6	5	2.36	−60
16	1	A	AlMe_3_	10	500	PhCl_2_	0.5	70	94	64	90	3	7	2	3.71	−55
17	1	A	AlMe_3_	10	100	PhCl_2_	1	25	13	0.9	94	2	4	4	2.23	−59
18	1	A	AlMe_3_	10	300	PhCl_2_	1	25	51	10	95	1	4	6	2.05	−62
19	1	A	AlMe_3_	10	800	PhCl_2_	0.5	25	100	109	94	3	3	5	2.10	−58
20	Sc*^g^*	A	AlMe_3_	10	500	PhCl_2_	2	25	21	4	93	4	3	0.9	2.18	−60
21	Sc*^g^*	A*^h^*	AlMe_3_	10	500	PhCl_2_	2	25	89	15	94	2	4	4	3.70	−60

*^a^* Conditions unless specified otherwise: 10 μmol of Ln complex, 10 μmol of activator, 5 mL of solvent. *^b^* Activator: **A** = [Ph_3_C][B(C_6_F_5_)_4_]; **B** = [PhNHMe_2_][B(C_6_F_5_)_4_]; **C** = B(C_6_F_5_)_3_. *^c^* Activity in 10^3^ g of polymer/(mol_Ln_ h). *^d^* Determined by ^1^H and ^13^C NMR spectrum: *c*-1,4: *cis*-1,4-selectivity; *t*-1,4: *trans*-1,4-selectivity; 3,4: 3,4-selectivity. *^e^* Determined by GPC in THF at 40 °C against polystyrene standard. *^f^* Measured by DSC. *^g^* Sc = Sc(CH_2_SiMe_3_)_3_(THF)_2_. *^h^* 20 μmol of activator.

Then, the influence of alkyl aluminum on the catalytic performance of the Sc complex **1**/**A** system was studied (Table 2, entries 4, 7–8). In comparison with PIP obtained by Al*^i^*Bu_3_ in entry 4, PIP obtained by AlEt_3_ had lower yield in long polymerization time (99% in 5 min), but higher molecular weight (*M*_n_ = 1000 kg/mol), narrower molecular weight distribution (*M*_w_/*M*_n_ = 1.78), and higher *cis*-1,4-selectivity (92%) (Table 2, entry 8). While PIP obtained by AlMe_3_ had the highest *cis*-1,4-selectivity, up to 94%, its yield, molecular weight, and molecular weight distribution were lower than those obtained in the above two cases (Table 2, entry 7). Therefore, the complex **1**/**A**/AlMe_3_ ternary system was identified as the optimum catalytic system for IP polymerization and was used for choosing the optimized polymerization conditions. Solvent, [Al]:[**1**] molar ratio, polymerization temperature, and [IP]:[**1**] molar ratio had an effect on yield, regio-/stereoselectivity, molecular weight, and molecular weight distribution of the resulting PIPs (Table 2, entries 7, 9–19). When the IP polymerization catalyzed by the Sc complex **1**/**A**/AlMe_3_ ternary system was carried out in different solvents such as Tol, PhCl, and PhCl_2_, the catalytic activity obtained in PhCl_2_ (1.7 × 10^4^ (g of polymer)/(mol_Ln_ h)) was higher than those obtained in Tol and PhCl (0.3 × 10^3^–1.3 × 10^4^ (g of polymer)/(mol_Ln_ h)), while the *cis*-1,4-selectivities of PIPs obtained in Tol and PhCl_2_ (94%) were higher than that obtained in PhCl (91%). When the [Al]:[**1**] molar ratio was decreased to 5:1, the catalytic activity and the PIP’s *cis*-1,4-selectivity and molecular weight did not change practically (Table 2, entries 10–11). In contrast, with the increasing [Al]:[**1**] molar ratio to 20:1, the catalytic activity and the PIP’s molecular weight slightly decreased, while the PIP’s *cis*-1,4-selectivity was still retained (Table 2, entries 10, 12). The PIPs obtained in low polymerization temperatures approximately −10 °C and 0 °C had higher *cis*-1,4-selectivities (95%–96%), higher molecular weights (*M*_n_: 700–1000 kg/mol), narrower molecular weight distributions (*M*_w_/*M*_n_: 1.80–2.10), but resulted in lower yields (Table 2, entries 10, 13–14). Moreover, the complex **1**/**A**/AlMe_3_ ternary system had a certain tolerance to the high polymerization temperatures of approximately 50 and 70 °C with increasing catalytic activities but decreasing *cis*-1,4-selectivity (Table 2, entries 10, 15–16). With the increasing [IP]:[**1**] molar ratio from 100:1 to 800:1, the catalytic activities gradually increased from 0.9 × 10^3^ (g of polymer)/(mol_Ln_ h) to 1.1 × 10^5^ (g of polymer)/(mol_Ln_ h), and the *cis*-1,4-selectivities of the resulting PIPs remained almost unchanged (Table 2, entries 10, 17–19). For comparison, IP polymerization was also carried out by use of the Sc(CH_2_SiMe_3_)_3_(THF)_2_/**A**/AlMe_3_ ternary system with molar ratios of 1:1:10 and 1:2:10 (Table 2, entries 20,21). The commonality of these three catalytic systems was the similar *cis*-1,4-selectivities of the resulting PIPs, but the catalytic activities of these three catalytic systems were different in decreasing order of the Sc complex **1**/**A**/AlMe_3_ ternary system, the Sc(CH_2_SiMe_3_)_3_(THF)_2_/**A**/AlMe_3_ ternary system with a molar ratio of 1:2:10, and the Sc(CH_2_SiMe_3_)_3_(THF)_2_/**A**/AlMe_3_ ternary system with a molar ratio of 1:1:10.

All of the resulting PIPs have good solubilities in THF and CHCl_3_. The ^1^H NMR spectra of these PIPs in CDCl_3_ indicate the presence of main 1,4-PIP unit and a trace amount of 3,4-PIP unit. The ^13^C NMR spectra of these PIPs give diagnostic signals assigned as main *cis*-1,4 configuration (*δ* = 23.6, 26.6, 32.4, 125.2, and 135.4 ppm) and a small amount of 3,4-configuration (*δ* = 18.8, 26.6, 32.4, 125.2, and 135.4 ppm) with or without a trace amount of *trans*-1,4-configuration (*δ* = 16.1, 26.6, 32.4, 125.2, and 135.4 ppm) (see supporting information). The GPC curves reveal that these main *cis*-1,4-PIPs have moderate to high molecular weights in the range of 100–1000 kg/mol and bimodal molecular weight distributions (*M*_w_/*M*_n_ = 1.78–3.71) similar to natural rubber. The DSC curves of these PIPs show the glass transition temperature (*T*_g_) in the range of –52 to –66 °C, consistent with the thermoplasticity of the *C*PIP (see Supporting Information).

### 3.4. Cis-1,4-Polymerization of MY by the Complexes **1**–**3**/Activator/AlR_3_ Ternary Systems

Similarly, the complexes **1**–**3**/activator/AlR_3_ ternary systems also served as highly efficient and regio-/stereoselective catalyst for the *cis*-1,4-polymerization of MY (Table 3). In pursuit of high *cis*-1,4-selectivity, the Y complex **3**/borane **C**/Al*^i^*Bu_3_ ternary system was chosen as the optimum catalytic system for MY polymerization since it showed the highest *cis*-1,4-selectivity up to 99% among these different ternary systems under similar condition in toluene (Table 3, entries 1–5). In comparison with MY polymerization in toluene, the Y complex **3**/**C**/Al*^i^*Bu_3_ ternary system demonstrated low activities, approximately 8 × 10^3^–1.1 × 10^4^ (g of polymer)/(mol_Ln_ h) and low *cis*-1,4-selectivities of approximately 95%–96% in MY polymerization in PhCl and PhCl_2_ (Table 3, entries 3,6,7). The amount of Al*^i^*Bu_3_ had an impact on the catalytic activity (Table 3, entries 3, 8–10). With an increase of the molar ratio of [Al]:[**3**] from 5:1 to 40:1, the activity first increased from 2 × 10^3^ (g of polymer)/(mol_Ln_ h) to 4.9 × 10^4^ (g of polymer)/(mol_Ln_ h), then decreased to 3.2 × 10^4^ (g of polymer)/(mol_Ln_ h) (Table 3, entries 3, 8–10). The *cis*-1,4-selectivities, molecular weights, and molecular weight distributions of the resulting PMYs remained constant or made small changes. In low polymerization temperature, approximately 0 °C, the complete *cis*-1,4-PMY (*cis*-1,4-selectivity of approximately 100%) was obtained with the highest molecular weight (*M*_n_ = 700 kg/mol) and the narrowest molecular weight distribution (*M*_w_/*M*_n_ = 1.66) (Table 3, entry 11). In high polymerization temperature, approximately 50 °C or 70 °C, the catalytic activities and the *cis*-1,4-selectivities gradually decreased, implying the instability of this catalyst in high temperatures (Table 3, entries 12–13). The concentration of MY monomer also affected the catalytic activity (Table 3, entries 3, 14–17). With a gradually increasing [MY]:[**3**] molar ratio from 250:1 to 4000:1, the activity gradually increased from 1.3 × 10^4^ (g of polymer)/(mol_Ln_ h) to 1.2 × 10^5^ (g of polymer)/(mol_Ln_ h), but at the same time the *cis*-1,4-selectivity of PMYs were retained (Table 3, entries 3, 14–17). By contrast, low activities approximately 0.2–1 (kg of polymer)/(mol_Ln_ h) and low *cis*-1,4-selectivities of approximately 95% were obtained by the Y(CH_2_SiMe_3_)_3_(THF)_2_/**C**/Al*^i^*Bu_3_ ternary system with different molar ratios as 1:1:10 and 1:2:10 under similar conditions (Table 3, entries 18,19).

**Table 3:**
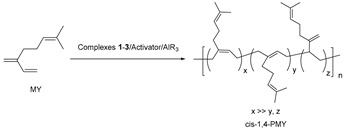
*Cis*-1,4-polymerization of myrcene by complexes **1**–**3**/activator/AlR_3_ ternary systems.*^a^*

entry	Cat.	A*^b^*				t (h)	T (°C)	Y (%)	A*^c^*	Microstructure (%)*^d^*				*M*_n_^*e*^ (10^5^)	*M*_w_/*M*_n_*^e^*	*T*_g_^*f*^ (°C)
			[Al]/[Ln]	[MY]/[Ln]	Sol.					*c*-1,4	*t*-1,4	3,4	1,2			
1	3	A	10	500	Tol	1	25	100	68	76	0	24	0	3	3.47	−60
2	3	B	10	500	Tol	1	25	100	68	80	0	20	0	3	2.56	−60
3	3	C	10	500	Tol	1	25	72	49	>99	0	0	0	4	1.93	−65
4	1	C	10	500	Tol	2	25	72	24	88	0	12	0	9	1.97	−60
5	2	C	10	500	Tol	48	25	6	0.002	93	0	7	0	2	2.51	−60
6	3	C	10	500	PhCl	8	25	99	8	95	0	5	0	4	2.24	−61
7	3	C	10	500	PhCl_2_	6	25	99	11	96	0	4	0	5	2.73	−61
8	3	C	5	500	Tol	2	25	7	2	99	0	1	0	6	2.58	−63
9	3	C	20	500	Tol	2	25	99	34	99	0	1	0	5	2.61	−63
10	3	C	40	500	Tol	2	25	94	32	98	0	2	0	3	4.26	−62
11	3	C	10	500	Tol	5	0	82	11	100	0	0	0	7	1.66	−67
12	3	C	10	500	Tol	2	50	78	27	98	0	2	0	5	3.01	−66
13	3	C	10	500	Tol	2	70	22	7	96	0	4	0	1	3.31	−60
14	3	C	10	250	Tol	2	25	78	13	99	0	1	0	6	2.71	−62
15	3	C	10	1000	Tol	2	25	75	51	100	0	0	0	5	1.75	−67
16	3	C	10	2000	Tol	2	25	61	83	99	0	1	0	7	1.96	−66
17	3	C	10	4000	Tol	3	25	65	118	99	0	1	0	9	1.76	−64
18	Y*^g^*	C	10	500	Tol	48	25	20	0.23	95	0	5	0	0.1	7.01	−60
19	Y*^g^*	C*^h^*	10	500	Tol	12	25	22	1	95	2	3	0	1	7.19	−60

*^a^* Conditions unless specified otherwise: 10 μmol of Ln complex, 10 μmol of activator, only Al*^i^*Bu_3_ as AlR_3_, 5 mL of solvent. *^b^* Activator: **A** = [Ph_3_C][B(C_6_F_5_)_4_]; **B** = [PhNHMe_2_][B(C_6_F_5_)_4_]; **C** = B(C_6_F_5_)_3_. *^c^* Activity in 10^3^ g of polymer/(mol_Ln_ h). *^d^* Determined by ^1^H and ^13^C NMR spectrum: *c*-1,4: *cis*-1,4-selectivity; *t*-1,4: *trans*-1,4-selectivity; 3,4: 3,4-selectivity; 1,2: 1,2-selectivity. *^e^* Determined by GPC in THF at 40 °C against polystyrene standard. *^f^* Measured by DSC. *^g^* Y = Y(CH_2_SiMe_3_)_3_(THF)_2_. *^h^* 20 μmol of activator.

All of the PMYs obtained by the complexes **1**–**3**/activator/Al*^i^*Bu_3_ systems were also soluble in THF and CHCl_3_. The ^1^H NMR spectra in CDCl_3_ demonstrate that these PMYs contained mainly 1,4-microstructure and a trace amount of 3,4-microstructure (see supporting information). The ^13^C NMR spectra display that these PMYs had mainly *cis*-1,4 configuration (*δ* = 17.86, 25.84, 26.96, 27.14, 30.79, 37.09, 124.63, 124.82, 131.34, and 139.16 ppm) and trace amount of 3,4-configuration (*δ* = 17.81, 25.81, 26.61, 32.32, 37.08, 47.55, 109.28, 124.63, 131.34, and 151.77 ppm) (see supporting information). By GPC analysis, these *cis-*1,4-PMYs had high molecular weights in the range of 100–900 kg/mol and bimodal molecular weight distributions (*M*_w_/*M*_n_ = 1.66–4.26). The glass transition temperature (*T*_g_) in the range of –60 °C to –67 °C was obtained for these *cis*-1,4-PMYs by DSC.

### 3.5. Syndiotactic Polymerization of ST by the Complexes **1**–**3**/Activator/AlR_3_ Ternary Systems

The complexes **1**–**3**/activator/AlR_3_ ternary systems could also promote the syndiotactic polymerization of ST (Table 4). By contrast, the Sc complex **1/B**/Al*^i^*Bu_3_ ternary system was the best catalytic system for ST polymerization since such a catalyst exhibited the highest catalytic activity, approximately 2.8 × 10^3^ (g of polymer)/(mol_Ln_ h) and the highest syndiotacticity up to 99% in PhCl_2_, affording SPST with the highest molecular weight and moderate molecular weight distribution (*M*_n_ = 900 kg/mol, *M*_w_/*M*_n_ = 2.38) (Table 4, entries 1–6). The solvent had a significant effect on syndiotacticity. When the ST polymerization was carried out in PhCl_2_ or Tol, the syndiotactic PSTs (SPSTs) with high syndiotacticities (*rrrr* up to 99%) were obtained (Table 4, entries 2, 9). While the PSTs with moderate syndiotacticities (*rrrr* in the range of 61% to 65%) were prepared in ST polymerization in PhCl and C_2_H_2_Cl_4_ (Table 4, entries 7,8,10). Different from the polymerization of conjugated dienes, the amount of Al*^i^*Bu_3_, polymerization temperature, and the concentration of ST monomer only had influence on catalytic activity instead of syndiotacticity of the Sc complex **1**/**B**/Al*^i^*Bu_3_ ternary system (Table 4, entries 2, 11–18). With the increasing molar ratio of [Al]/[Ln] from 5:1 to 15:1, the activity first increased from 0.2 × 10^3^ (g of polymer)/(mol_Ln_ h) to 2.8 × 10^3^ (g of polymer)/(mol_Ln_ h) then decreased to 1.8 × 10^3^ (g of polymer)/(mol_Ln_ h). When the polymerization temperature rose from 25 °C to 90 °C, the activity slightly dropped from 2.8 × 10^3^ (g of polymer)/(mol_Ln_ h) to 2.6 × 10^3^ (g of polymer)/(mol_Ln_ h), suggesting that such catalyst is very stable in high polymerization temperatures (Table 4, entries 2, 13–15). Moreover, the activity went up from 0.7 × 10^3^ (g of polymer)/(mol_Ln_ h) to 2.8 × 10^3^ (g of polymer)/(mol_Ln_ h) then declined to 2.1 × 10^3^ (g of polymer)/(mol_Ln_ h) with the gradually increasing [St]:[**1**] molar ratio from 200:1 to 700:1 (Table 4, entries 2, 16–18). In the above cases, the resulting SPSTs always had complete syndiotacticities up to 99%. In comparison, the Sc(CH_2_SiMe_3_)_3_(THF)_2_/**B**/Al*^i^*Bu_3_ ternary system with the molar ratio of 1:1:10 produced PST with complete syndioselectivity (*rrrr* > 99%) similar to the Sc complex **1**/**B**/Al*^i^*Bu_3_ ternary system, while the Sc(CH_2_SiMe_3_)_3_(THF)_2_/**B**/Al*^i^*Bu_3_ ternary system with the molar ratio of 1:1:10 only afforded PST with moderate syndioselectivity (*rrrr* = 78%) under similar conditions (Table 4, entries 19–20).

**Table 4:**
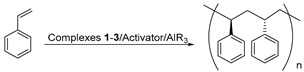
Syndiotactic polymerization of styrene by complexes **1**–**3**/activator/AlR_3_ ternary systems.*^a^*

							t	T	Y		*rrrr^d^*	*M*_n_^*e*^		*T*_m_^*f*^
entry	Cat.	A*^b^*	AlR_3_	[Al]/[Ln]	[ST]/[Ln]	Sol.	(h)	(°C)	(%)	A*^c^*	(%)	(10^5^)	*M*_w_/*M*_n_^*e*^	(°C)
1	1	A	Al*^i^*Bu_3_	10	500	PhCl_2_	12	25	18	781	>99	7	2.05	272
2	1	B	Al*^i^*Bu_3_	10	500	PhCl_2_	6	25	32	2777	>99	9	2.38	271
3	1	C	Al*^i^*Bu_3_	10	500	PhCl_2_	24	25	7	152	>99	3	2.36	274
4	2	B	Al*^i^*Bu_3_	10	500	PhCl_2_	24	25	6	130	54	n.d.	n.d.	260
5	3	B	Al*^i^*Bu_3_	10	500	PhCl_2_	24	25	23	499	>99	9	2.21	271
7	1	B	AlEt3	10	500	PhCl_2_	24	25	10.4	225.66	>99	0.07	6.42	
6	1	B	AlEt_3_	10	500	PhCl_2_	24	25	10	217	>99	0.1	6.42	271
7	1	A	Al*^i^*Bu_3_	10	500	PhCl	24	25	26	564	63	n.d.	n.d.	265
8	1	B	Al*^i^*Bu_3_	10	500	PhCl	24	25	17	369	65	n.d.	n.d.	267
9	1	B	Al*^i^*Bu_3_	10	500	Tol	20	25	43	1120	>99	7	2.27	270
10	1	B	Al*^i^*Bu_3_	10	500	C_2_H_2_Cl_4_	48	25	17	184	61	n.d.	n.d.	265
11	1	B	Al*^i^*Bu_3_	5	500	PhCl_2_	12	25	5	217	>99	n.d.	n.d.	272
12	1	B	Al*^i^*Bu_3_	15	500	PhCl_2_	12	25	42	1822	>99	6	2.09	271
13	1	B	Al*^i^*Bu_3_	10	500	PhCl_2_	12	50	60	2604	>99	5	2.00	273
14	1	B	Al*^i^*Bu_3_	10	500	PhCl_2_	12	70	59	2561	>99	4	1.98	272
15	1	B	Al*^i^*Bu_3_	10	500	PhCl_2_	12	90	61	2647	>99	0.1	18.78	271
16	1	B	Al*^i^*Bu_3_	10	200	PhCl_2_	12	25	41	712	>99	5	2.02	275
17	1	B	Al*^i^*Bu_3_	10	400	PhCl_2_	12	25	35	1215	>99	8	2.02	273
18	1	B	Al*^i^*Bu_3_	10	700	PhCl_2_	12	25	35	2126	>99	13	1.49	271
19	Sc*^g^*	B	Al*^i^*Bu_3_	10	500	PhCl_2_	48	25	7	76	78	n.d.	n.d.	268
20	Sc*^g^*	B*^h^*	Al*^i^*Bu_3_	10	500	PhCl_2_	12	25	9	390	>99	n.d.	n.d.	272

*^a^* Conditions unless specified otherwise: 10 μmol of Ln complex, 10 μmol of activator, 5 mL of solvent. *^b^* Activator: **A** = [Ph_3_C][B(C_6_F_5_)_4_]; **B** = [PhNHMe_2_][B(C_6_F_5_)_4_]; **C** = B(C_6_F_5_)_3_. *^c^* Activity in (g of polymer)/(mol_Ln_ h). *^d^* Determined by ^1^H and ^13^C NMR spectrum. *^e^* Determined by GPC in 1,2,4-trichlorobenzene at 150 °C against polystyrene standard. *^f^* Measured by DSC. *^g^* Sc = Sc(CH_2_SiMe_3_)_3_(THF)_2_. *^h^* 20 μmol of activator.

The resulting SPSTs are soluble in PhCl_2_ and C_2_H_2_Cl_4_ in high temperatures. The ^13^C NMR spectra in CDCl_3_ indicate that these SPSTs have complete syndiotactic-microstructure with only a singlet at 145.35 ppm (see Supporting Information). GPC curves display that these SPSTs have moderate to high molecular weights in the range of 10–900 kg/mol and bimodal molecular weight distributions (*M*_w_/*M*_n_ = 1.98–18.78). As demonstrated by DSC, the melting points (*T*_m_) of these SPSTs are in the range of 260 °C to 275 °C (see Supporting Information).

### 3.6. Polymerization Mechanism Study

**Chart 2:**
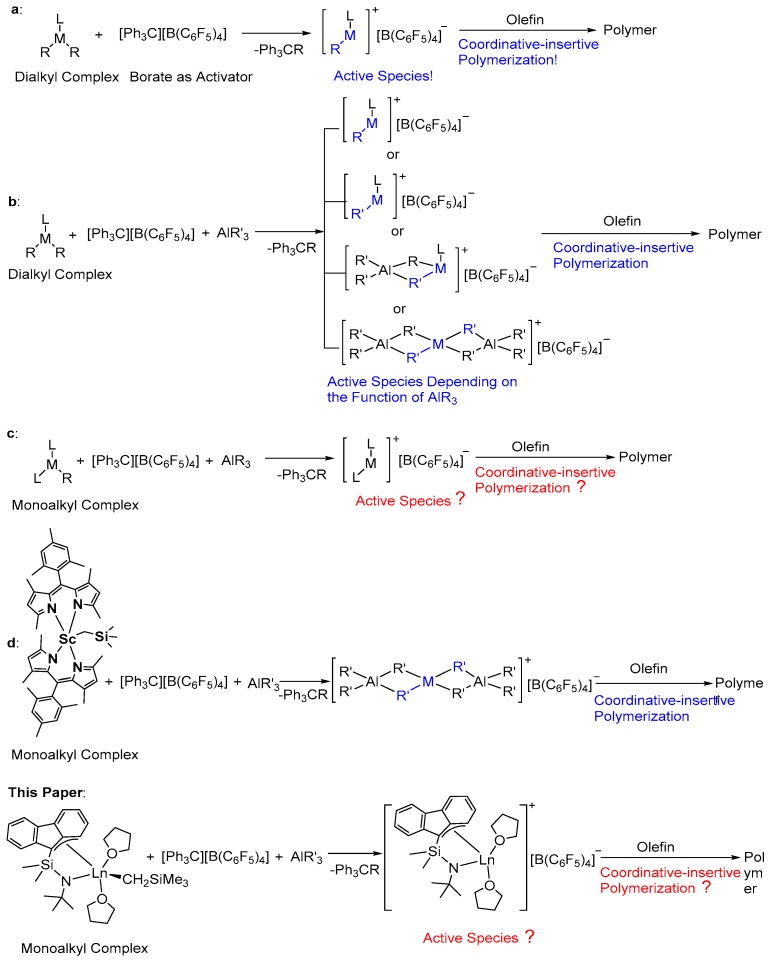
Coordination–insertion polymerization of olefin by using the different rare earth metal catalysts.

**Figure 2:**
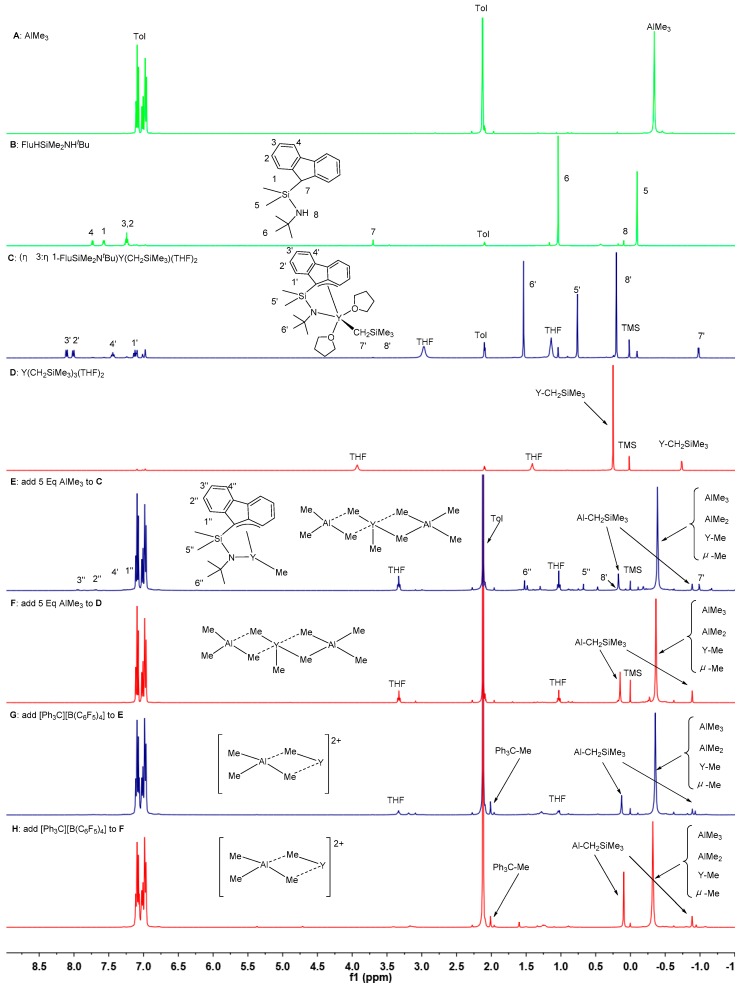
In situ ^1^H NMR spectra of active species by using the Y complex **3**/[Ph_3_C][B(C_6_F_5_)_4_]/AlMe_3_ ternary system in *d*-C_7_H_8_ at 25 °C.

In general, the catalytically active species in the coordination–insertion polymerization of olefin is usually generated from the rare earth metal dialkyl/dihalides complex and activator with or without AlR_3_ (Chart 2a,b) [8]. In such catalytic systems, an activator usually eliminates one alkyl group from the metal center to afford cationic species containing a metal–alkyl (Ln–R) bond for the coordination and insertion of olefin monomer to finally give polyolefin with different regio-/stereoselectivity and microstructure. Unlike an activator, AlR_3_ can perform a lot of functions in olefin polymerization, such as scavenging impurities, transforming (alkylating and reducing) the cationic species, and/or acting as a chain transfer agent [34,35,36]. Later, AlR_3_, especially AlMe_3_, was found to react with the rare earth metal trialkyl/dialkyl complex to form a heterobimetallic tetraalkylaluminate complex as a catalyst precursor. Activated by an activator, the corresponding cationic heterobimetallic tetraalkylaluminate complex was formed as a truly active species in the olefin polymerization (Chart 2b) [37,38,39]. Recently, AlR_3_ was found to remove coordinated solvent molecules such as THF or pyridine from the metal center or transfer the anionic chelating ligand from the rare earth metal center (Chart 2b) [40,41,42,43]. But in comparison, the catalytically active species in the coordination–insertion polymerization of olefins by use of rare earth metal monoalkyl complex/activator/AlR_3_ ternary system is difficult to calculate and understand (Chart 2c). According to the conventional synthesis method **a**, the resulting cationic species does not have the alkyl group, which inhibits coordination and insertion of olefin monomer into the Ln–R bond. As a result, the high regio-/stereoselective polymerization of olefins can’t occur. Therefore, some special reaction must be happened during the formation of cationic active species. More recently, we found that in the *cis*-1,4-polymerization of isoprene catalyzed by the dipyrromethene ligated scandium monoalkyl complex/activator/AlR_3_ ternary system, both of two anionic dipyrromethene chelating ligands transferred from the Sc center to the Al center. This was observable by using naked eyes, UV irradiation, fluorescence spectrum, and in situ ^1^H NMR spectrum, and affords a catalyst precursor heterobimetallic tetraalkylaluminate complex (Chart 2d) [40]. Then, one alkyl group of such catalyst precursor was removed by an activator to form the cationic heterobimetallic tetraalkylaluminate complex as a truly active species in *cis*-1,4-polymerization of IP (Chart 2d). In this paper, these Flu-based CGC allyl-type rare earth metal monoalkyl complexes/activator/AlR_3_ ternary system also exhibited high regio-/stereoselectivities and/or high activities in the polymerization of olefins such as IP, MY, and ST. The catalytically active species of such ternary systems in the coordination–insertion polymerization of olefins also aroused our interest. Therefore, the polymerization initiation processes by the Y complex **3**/activator/AlR_3_ ternary systems under different [Ln]/[AlR_3_] molar ratios were monitored by use of the in situ ^1^H NMR spectrum in *d*-toluene or *d*-THF at 25 °C (Figure 2 and supporting information).

The analysis of the in situ ^1^H NMR spectra of the active species generated from the Y complex **3**/[Ph_3_C][B(C_6_F_5_)_4_]/AlMe_3_ ternary system in *d*-toluene at 25 °C was taken as an example (Figure 2). Firstly, the reaction between the Y complex **3** and 5 equivalent units of AlMe_3_ was carried out in a J. Young valve NMR tube for 5 min consistent with polymerization procedure (Figure 2E). The in situ ^1^H NMR spectrum demonstrated that the peaks assigned to the dianionic FluSiMe_2_N*^t^*Bu ligand became weak and moved to the high field. Moreover, the position of peaks assigned to the coordinated THF molecules and AlMe_3_ had obvious changes. It was very interesting that this ^1^H NMR spectrum very much looked like the in situ ^1^H NMR spectrum of the reaction of Y(CH_2_SiMe_3_)_3_(THF)_2_ and 5 equivalent units of AlMe_3_ after 5 min in *d*-toluene at 25 °C, in which the heterobimetallic tetramethylaluminate complex Y(Me)[(*μ*-Me)_2_Al(Me)_2_]_2_ was formed with a broad signal at –0.3 ppm for all of the methyl groups (slightly different with AlMe_3_) in addition with the byproduct Al(CH_2_SiMe_3_)_3_ (Figure 2F). These results implied that the Y complex **3** had decomposed during this reaction. In view that no free FluHSiMe_2_NH*^t^*Bu ligand was observed from the above in situ ^1^H NMR spectrum, the dianionic FluSiMe_2_N*^t^*Bu ligand should precipitate from the polymerization solvent. Then 1 equivalent unit of activator [Ph_3_C][B(C_6_F_5_)_4_] was added to the NMR tube in order to generate cationic species. Almost no peaks assigned to the dianionic FluSiMe_2_N*^t^*Bu ligand were found in the in situ ^1^H NMR spectrum, identifying the precipitation of an insoluble complex containing the dianionic FluSiMe_2_N*^t^*Bu ligand (Figure 2G). Similarly, this ^1^H NMR spectrum and the in situ ^1^H NMR spectrum of the reaction of Y(Me)[(*μ*-Me)_2_Al(Me)_2_]_2_ with 2 equivalent units of [Ph_3_C][B(C_6_F_5_)_4_] (Figure 2H) were nearly identical. The chemical shift of the main peak at –0.3 ppm was similar to that of the main peak of the cationic heterobimetallic tetramethylaluminate complex [(Me)_2_Al(*μ*-Me)_2_Y]^2+^[B(C_6_F_5_)_4_]^2−^. Meanwhile, a new peak assigned as byproduct Ph_3_CCH_3_ also appeared at 2.0 ppm. Such results identified the formation of cationic species [(Me)_2_Al(*μ*-Me)_2_Y]^2+^[B(C_6_F_5_)_4_]^2−^ from the Y complex **3**/[Ph_3_C][B(C_6_F_5_)_4_]/AlMe_3_ ternary system. The similar results were also obtained from the in situ ^1^H NMR spectra of the Y complex **3**/activator/AlR_3_ ternary systems in *d*-THF at 25 °C (Supporting Information). Based on these facts, it is guessed that AlMe_3_ firstly removes two coordinated THF molecules from the Y center of complex **3** to give (FluSiMe_2_N*^t^*Bu)Y(CH_2_SiMe_3_) as an intermediate. Then the alkyl exchange between the Y center of the above intermediate and the Al center of AlMe_3_ forms a new Y complex (FluSiMe_2_N*^t^*Bu)YMe and a byproduct Al complex AlMe_2_(CH_2_SiMe_3_). Similar to the previous (DPM)_2_ScR/activator/AlR_3_ ternary system [40], the dianionic FluSiMe_2_N*^t^*Bu ligand of (FluSiMe_2_N*^t^*Bu)LnMe immediately transfers from the Y center to the Al center to produce the heterobimetallic tetramethylaluminate complex (Me)_2_Al(*μ*-Me)_2_Y(Me)(*μ*-Me)_2_Al(Me)_2_ as a catalyst precursor and the insoluble Y-Aluminum salt solid [(Me)_2_Al(*μ*-Me)_2_Y(*μ*-Me)_2_Al(Me)_2_][Al(FluSiMe_2_N*^t^*Bu)_2_] as a byproduct. Such a rapid exchange of the FluSiMe_2_N*^t^*Bu ligand from the Y center to the Al center is also observed in the in situ ^1^H NMR spectra when AlEt_3_ or Al*^i^*Bu_3_ was used as AlR_3_ (see Supporting Information). Although the transference of monodentate or bidentate ligand from the Y center to the Al center to form the heterobimetallic tetramethylaluminate complex LY[(*μ*-Me)_2_AlMe_2_]_n_ have been reported previously by Anwander [41], Hou [42], and Kempe [43], this is the first discussion of the transference of dianionic CGC allyl-type chelating ligand. Later, in the presence of 2 equivalent units of activator [Ph_3_C][B(C_6_F_5_)_4_], a divalent cationic heterobimetallic tetramethylaluminate species [(Me)_2_Al(*μ*-Me)_2_Y]^2+^[B(C_6_F_5_)_4_]^2−^ was obtained, in combination with Ph_3_CCH_3_ as a byproduct.

**Scheme 2:**
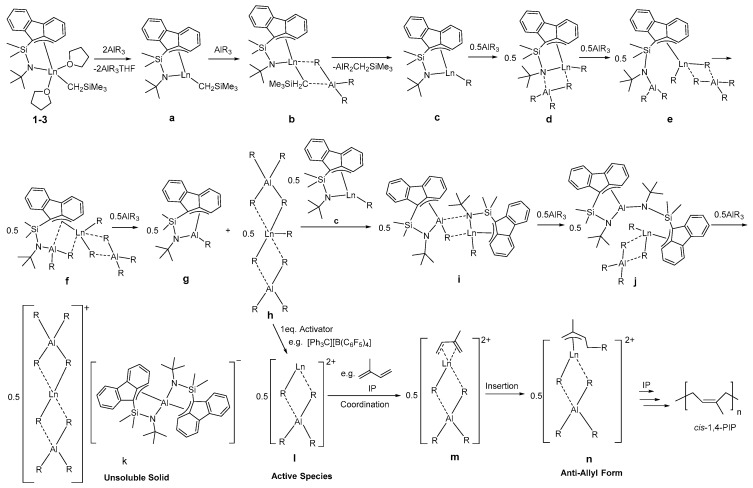
Possible mechanism process for the coordination–insertion polymerization of olefin by Flu-based CGC allyl-type rare-earth metal complexes **1**–**3**/activator/AlR_3_ ternary systems.

Taking above results into account, a plausible coordination–insertion mechanism is proposed for the regio-/stereoselective polymerization of olefins catalyzed by the Flu-based CGC allyl-type rare earth monoalkyl complexes (η^3^:η^1^-FluSiMe_2_N*^t^*Bu)Ln(CH_2_SiMe_3_)(THF)_2_ (**1**–**3**)/activator/AlR_3_ ternary systems in Scheme 2. At first, 2 equivalent units of AlR_3_ abstracts two coordinated THF molecules from the rare earth metal center of these complexes **1**–**3** to produce intermediate (η^3^:η^1^-FluSiMe_2_N*^t^*Bu)Ln(CH_2_SiMe_3_) (**a**). Then the exchange of the CH_2_SiMe_3_ group of **a** and alkyl group of AlR_3_ gives a new rare earth metal alkyl complex (η^3^:η^1^-FluSiMe_2_N*^t^*Bu)LnR (**c**) with the release of AlR_2_(CH_2_SiMe_3_). The continued ligand exchange between metal center of 0.5 equivalent units of the above intermediate **c** and Al center of AlR_3_ forms 0.5 equivalent units of a heterobimetallic tetraalkylaluminate complex (R)_2_Al(*μ*-R)_2_Ln(R)(*μ*-R)_2_Al(R)_2_ (**h**) as catalyst precursor and 0.5 equivalent units of (η^3^:η^1^-FluSiMe_2_N*^t^*Bu)AlR (**g**). Then the 0.5 equivalent units of **g** continues to react with the other 0.5 equivalent units of **c** to finally give an insoluble Y–Aluminum salt solid [(R)_2_Al(*μ*-R)_2_Ln(*μ*-R)_2_Al(R)_2_]^+^[Al(η^3^:η^1^-FluSiMe_2_N*^t^*Bu)_2_]^−^ (**k**) as a byproduct. Then two alkyl groups of 0.5 equivalent units of the catalyst precursor **h** are removed from metal center by 1 equivalent unit of activator to finally generate a divalent cationic species {Ln[(*μ*-R)_2_AlR_2_]^2+^} (**l**) as catalytically active species in the regio-/stereoselective polymerization of olefins. Based on the actual circumstance of syndiotactic polymerization of ST by the Y complex **3**/**B**/Al*^i^*Bu_3_ ternary system and the Sc(CH_2_SiMe_3_)_3_(THF)_2_/**B**/Al*^i^*Bu_3_ ternary system with different molar ratios as 1:1:20 and 1:1:10 in Table 4, such a divalent cationic active species is quite reasonable. The less steric hindrance around the metal center of this cationic species permits the coordination and insertion of IP/MY monomers in *cis*-1,4 mode to form the *anti*-allyl form intermediate, which finally affords the *cis*-1,4-PIPs/PMYs with *cis*-1,4-selectivities up to 96% or 100%. Such high *cis*-1,4-selectivity in the coordination–insertion polymerization of conjugated dienes is in agreement with the high *cis*-1,4-selectivity obtained by the heterobimetallic tetraalkylaluminate active species {LLn[(*μ*-Me)_2_AlMe_2_]^2+^} in the isoprene polymerization [40,41]. Similarly, such a cationic species also promotes the backbiting of the last phenyl group in PST chains with a metal center. As a result, the SPSTs are obtained by such Flu-based CGC allyl-type rare earth monoalkyl complexes **1**–**3**/activator/AlR_3_ ternary systems.

## 4. Conclusions

In summary, three Flu-based CGC allyl-type rare earth metal monoalkyl complexes **1**–**3** have been easily synthesized in moderate to high yields and structural characterization by ^1^H and ^13^C NMR spectrum, elemental analyses as well as X-ray diffraction. In the presence of cocatalyst activator borate [Ph_3_C][B(C_6_F_5_)_4_] (**A**) and AlMe_3_, these complexes **1**–**3** exhibit moderate activities from 0.3 × 10^3^ (g of polymer)/(mol_Ln_ h) to 1.1 × 10^6^ (g of polymer)/(mol_Ln_ h) and high *cis*-1,4-selectivities up to 96% in IP polymerization in PhCl_2_, yielding the main *cis*-1,4-PIPs with high molecular weights (*M*_n_ up to 1000 kg/mol) and bimodal molecular weight distributions (*M*_w_/*M*_n_ = 1.78–3.71). Activated by the cocatalyst borane B(C_6_F_5_)_3_ (**C**) and Al*^i^*Bu_3_, these complexes **1**–**3** display high activities up to 1.2 × 10^5^ (g of polymer)/(mol_Ln_ h) and high *cis*-1,4-selectivities up to 100% in MY polymerization in Tol, producing the *cis*-1,4-PMYs with high molecular weights (*M*_n_ up to 900 kg/mol) and bimodal molecular weight distributions (*M*_w_/*M*_n_ = 1.66–3.47). Moreover, these complexes **1**–**3**/[PhNHMe_2_][B(C_6_F_5_)_4_] (**B**)/Al*^i^*Bu_3_ ternary systems also promote the syndiotactic polymerization of ST with moderate activities up to 2.8 × 10^3^ (g of polymer)/(mol_Ln_ h) in PhCl_2_ to give SPSTs with high molecular weights (*M*_n_ up to 1300 kg/mol) and bimodal molecular weight distributions (*M*_w_/*M*_n_ = 1.49–18.78). In comparison with no, or very low activity and regio-/stereoselectivity of the previous CGC allyl type rare earth metal complexes, such results demonstrate that the transfer of the chelating ligand from the rare earth metal center to the Al center to form the heterobimetallic tetraalkylaluminate complex plays a key role on the excellent catalytic performance of these CGC allyl-type rare earth metal monoalkyl complexes in olefin polymerization. These findings will benefit the design of highly efficient and regio-/stereoselective rare earth metal catalysts as well as the precise synthesis of natural rubber. Further studies will be focused on the research of excellent rare earth metal catalysts for the polymerization of olefins.

## Figures and Tables

**Table 1:** Selected bond distances (Å) and angles (deg) of complexes **1**–**3**.

	1	2	3
Ln−N1	2.065(3)	2.180(3)	2.213(3)
Ln−C1	2.395(3)	2.504(4)	2.566(3)
Ln−C2	2.730(3)	2.746(4)	2.769(3)
Ln−C3	3.067(3)	3.044(4)	3.036(4)
Ln−C20	2.226(4)	2.338(4)	2.392(4)
N1−Ln−C20	109.34(13)	111.36(14)	112.29(13)
N1−Ln−C1	75.05(11)	71.80(13)	70.57(11)
C20−Ln−C1	144.95(13)	148.23(15)	149.02(13)
N1−Ln−C2	91.58(11)	88.71(12)	87.82(11)
C20−Ln−C2	113.15(12)	117.13(14)	118.36(13)
C1−Ln−C2	32.52(11)	31.92(13)	31.58(11)
N1−Ln−C3	86.93(11)	85.21(13)	84.56(11)
C20−Ln−C3	89.52(11)	93.24(14)	94.39(12)
C1−Ln−C3	55.57(11)	55.06(13)	54.68(11)
C2−Ln−C3	27.22(10)	27.32(12)	27.47(11)

## Supplementary Materials

The following are available online at https://www.mdpi.com/2073-4360/11/5/836/s1.
