# Supplementary material for: Unprecedentedly High Activity and/or High Regio-/Stereoselectivity of Fluorenyl-Based CGC Allyl-Type η3:η1-tert-Butyl(dimethylfluorenylsilyl)amido Ligated Rare Earth Metal Monoalkyl Complexes in Olefin Polymerization
Source: Polymers (Basel). 2019 May 8;11(5):836. doi: 10.3390/polym11050836 (PMC6572029; doi:10.3390/polym11050836)
Supplement: Supplementary file 1 [file polymers-11-00836-s001.pdf]

Polymers **2019**

Supplementary Materials: Unprecedentedly High Activity and/or High Regio-/Stereoselectivity of Fluorenyl-Based CGC Allyl-Type η³:η¹-*tert*-Butyl(dimethylfluorenylsilyl)amido Ligated Rare Earth Metal Monoalkyl Complexes in Olefin Polymerization

Ge Guo [1], Xiaolu Wu [1], Xiangqian Yan [1], Li Yan [2], Shaowen Zhang [1,*] Nannan Qiu [3,*] and Xiaofang Li [1,*]

Synthesis of FluHSiMe₂NH^{t}Bu Ligand

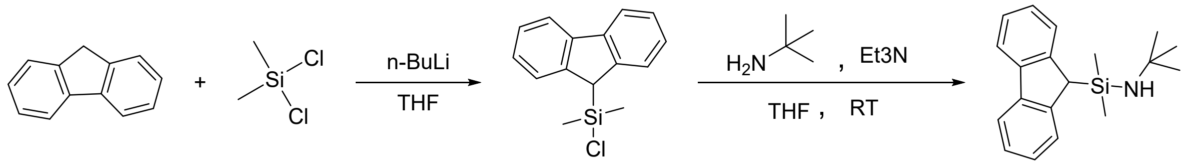

L1-a L1

FluHSiMe₂NH^{t}Bu was synthesized following literature procedure [1]:

Synthesis of intermediate product L1-a: In a glove box, Fluorene (3.33 g, 20.0 mmol) was dissolved in an appropriate amount of dry THF in a reaction flask, then placed in the glove box refrigerator for 15 min. *n*-BuLi (8.35 ml, 2.4 mol/L) was added dropwise into the cold reaction system, and the reaction mixture was stirred at room temperature for 1 h, then transferred to a constant pressure dropping funnel for use. Under nitrogen atmosphere, at –10 °C, the reaction solution was added dropwise to dichlorodimethylsilane (10.33 g, 80.0 mmol), and the mixture was stirred at room temperature for 2 h. The reaction mixture was evaporated to dryness, then washed with a large portion of *n*-hexane, filtered and evaporated to give a pale yellow solid (3.19 g, 62%). ^{1}H NMR (400 MHz, $CDCl_3$) δ 7.89 (d, J = 7.5 Hz, 2H), 7.69 (d, J = 7.5 Hz, 2H), 7.41 (d, J = 7.4 Hz, 2H), 7.36 (t, J = 7.1 Hz, 2H), 4.12 (s, 1H), 0.20 (s, 6H).

Synthesis of FluHSiMe₂NH^{t}Bu Ligand L1: Compound L1-a (2.59 g, 10.0 mmol) was dissolved in 30 ml of dry THF under a nitrogen atmosphere. After the solution was cooled to 0 °C, *t*-butylamine (0.74 g, 10.0 mmol) and triethylamine (3.04 g, 30.0 mmol) were added dropwise slowly. After the completion of the dropwise addition, the reaction solution was stirred for 5 h at room temperature. The reaction mixture was evaporated to dryness, then washed with a large portion of *n*-hexane, filtered and evaporated to give a yellow oily substance. The light green oily ligand (2.48 g, 84%) is obtained from the yellow oily substance by vacuum distillation at 150 °C. ^{1}H NMR (400 MHz, $CDCl_3$) δ 7.89 (d, J = 7.3 Hz, 2H), 7.68 (d, J = 7.4 Hz, 2H), 7.36 (dt, J = 18.7, 7.2 Hz, 4H), 3.96 (s, 1H), 1.25 (s, 9H), 0.72 (s, 1H), –0.04 (s, 6H). ^{13}C NMR (100 MHz, $CDCl_3$) δ 145.85 (s), 140.77 (s), 125.90 (s), 125.16 (s), 124.52 (s), 119.88 (s), 49.72 (s), 45.04 (s), 33.98 (s), –0.73 (s).

^{1}H and ^{13}C NMR Spectra

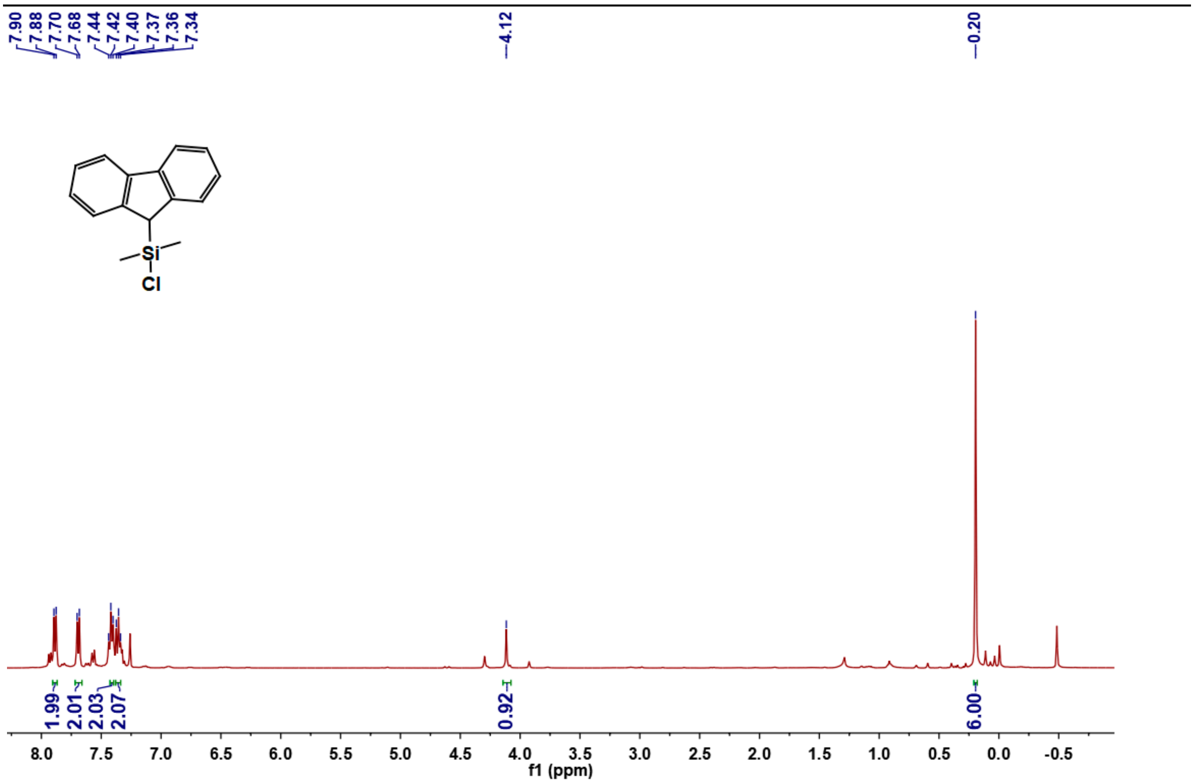

Figure S1. ^{1}H NMR spectra of L1-a.

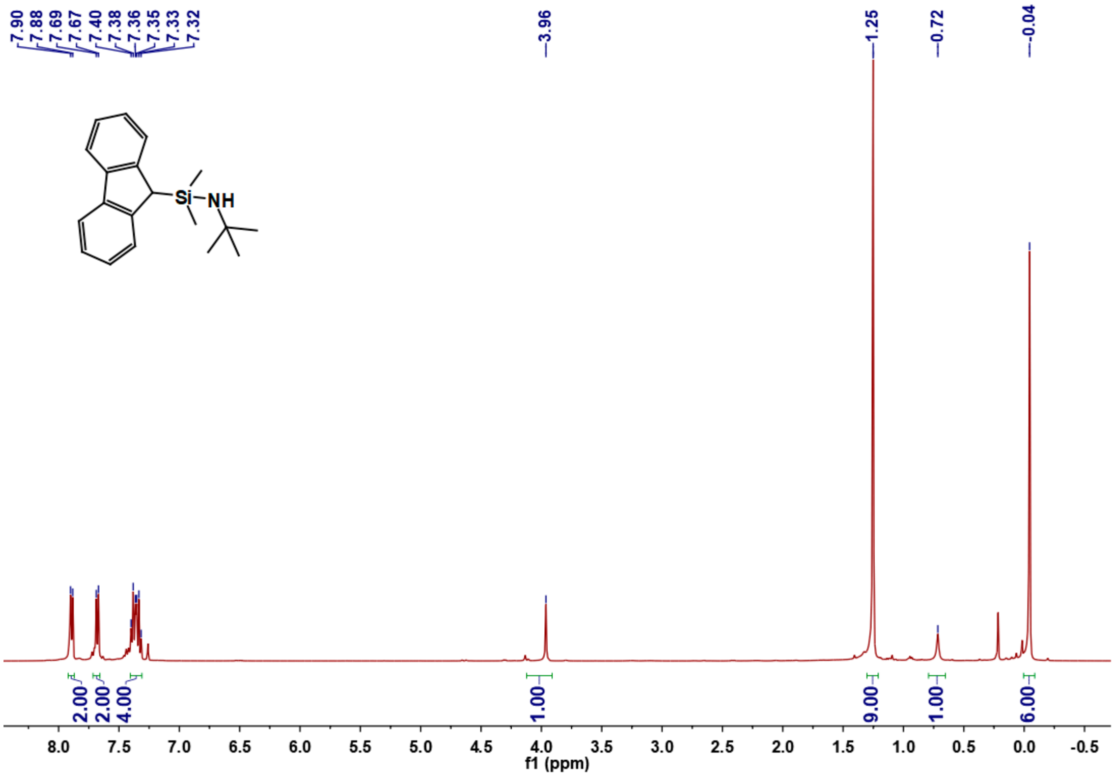

Figure S2. ^{1}H NMR spectra of L1.

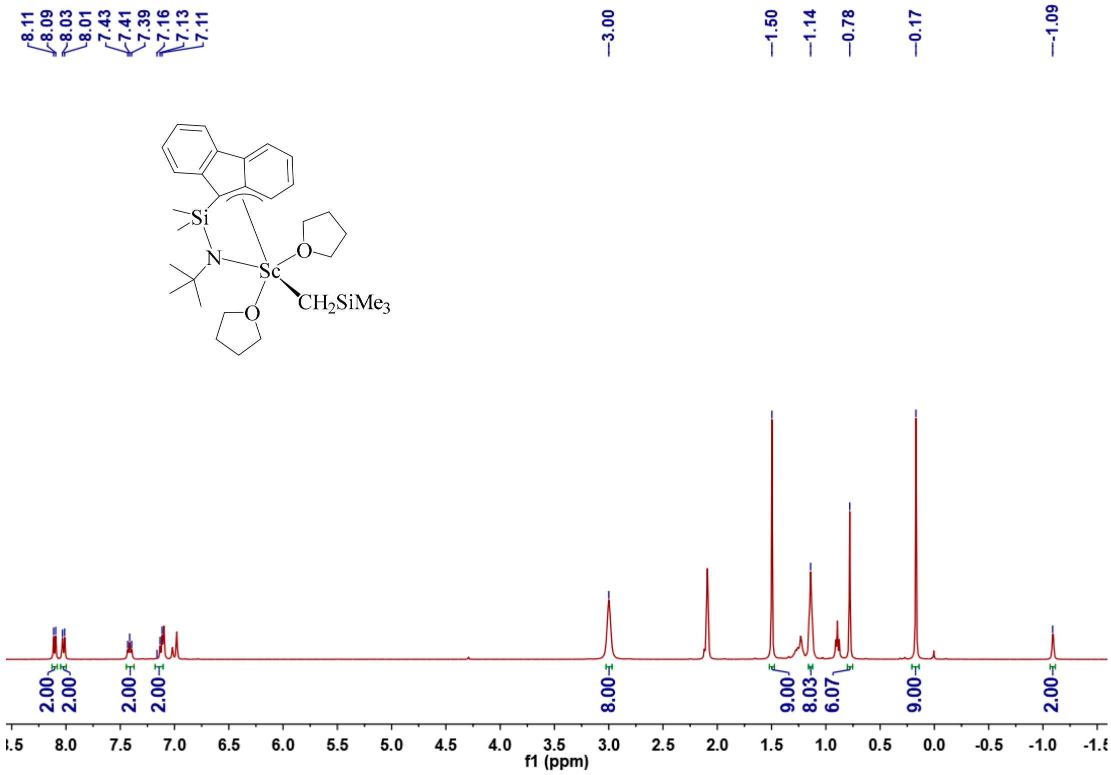

Figure S3. ^{1}H NMR spectra of complex **1.**

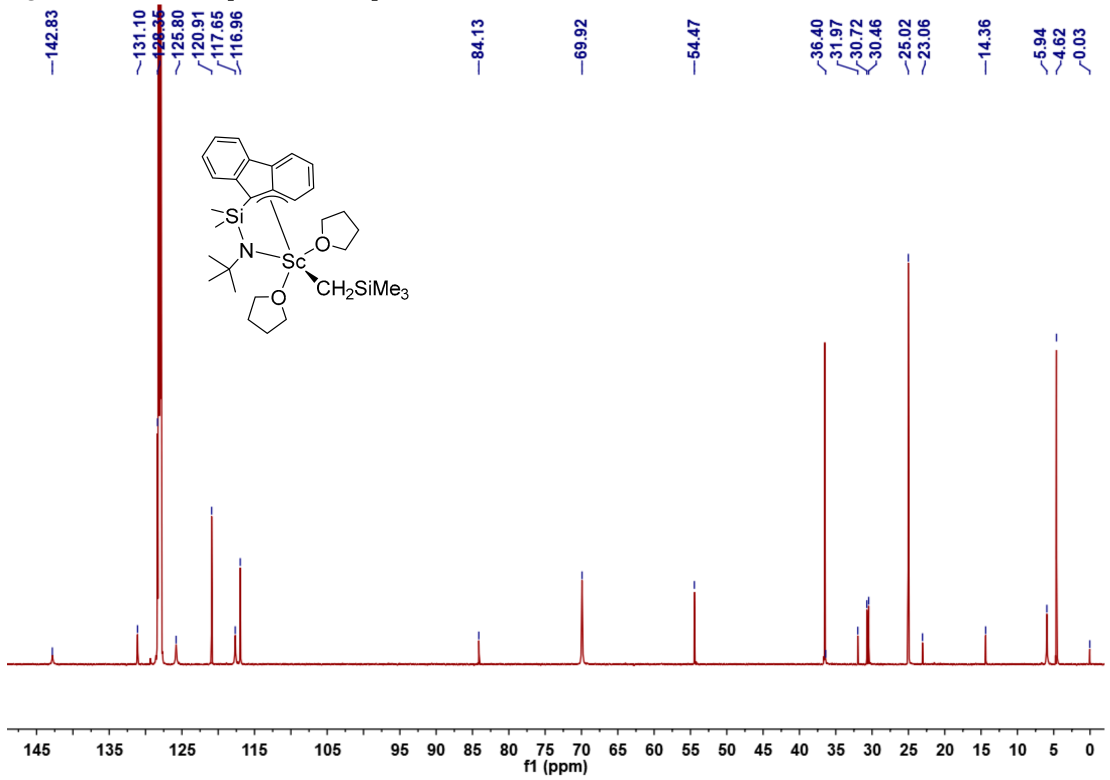

Figure S4. ^{13}C NMR spectra of complex **1.**

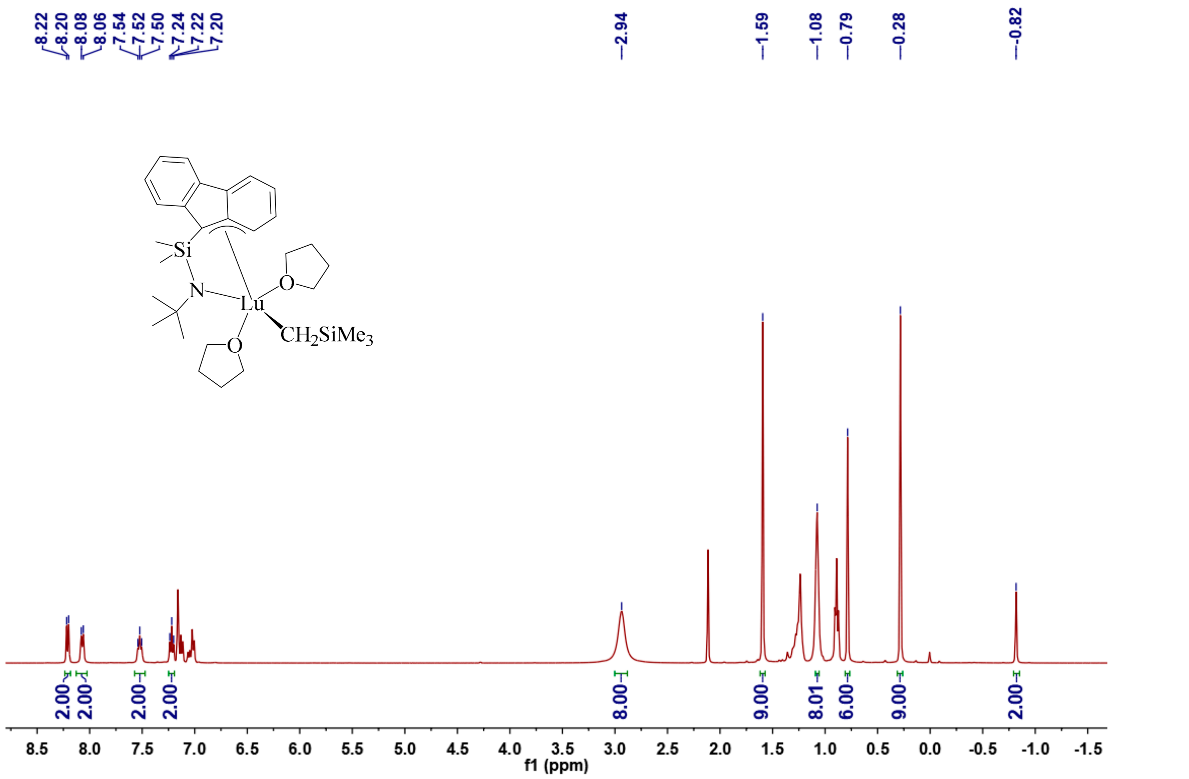

Figure S5. ^{1}H NMR spectra of complex **2.**

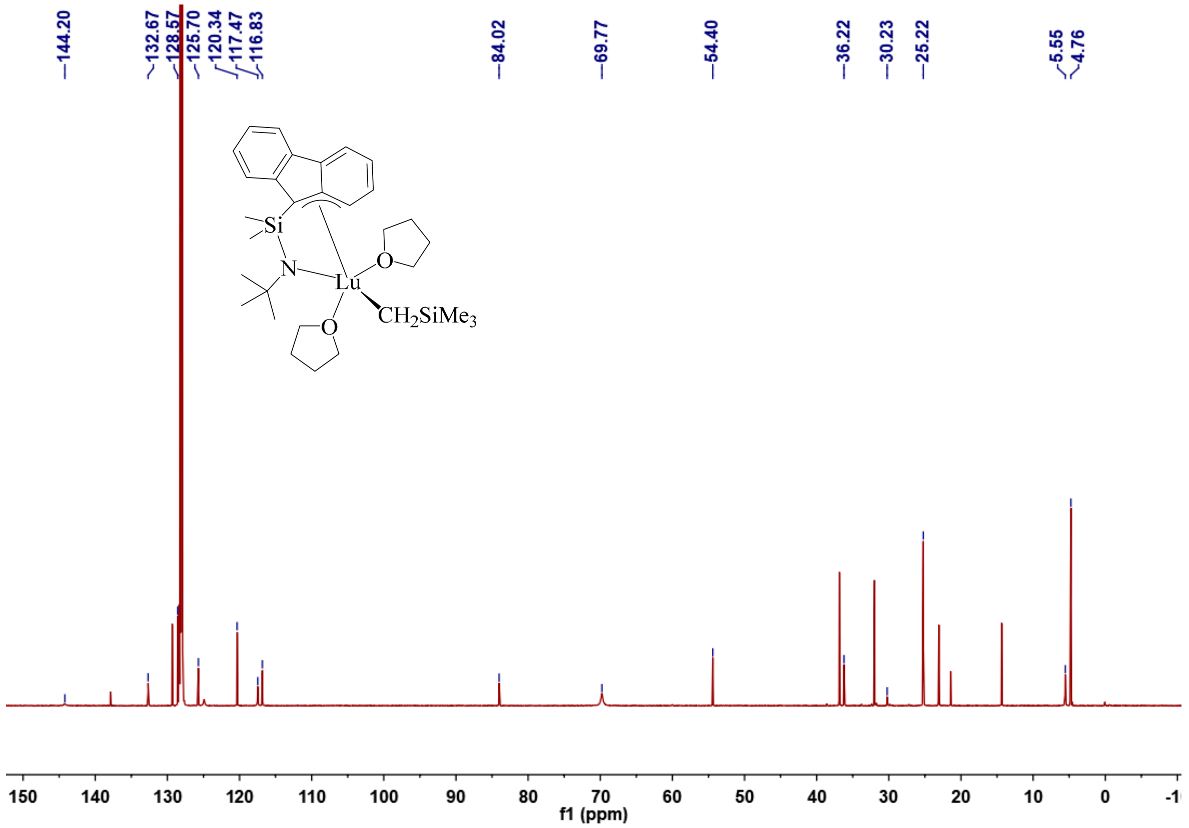

Figure S6. ^{13}C NMR spectra of complex **2.**

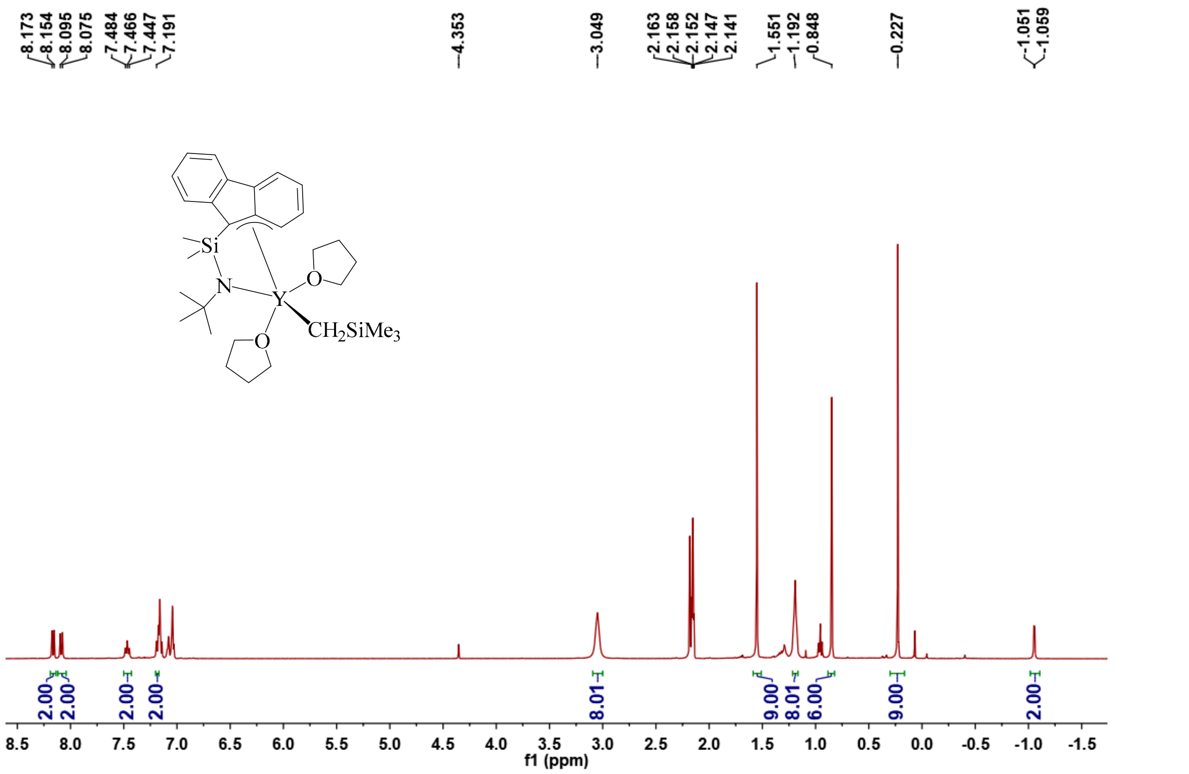

Figure S7. ^{1}H NMR spectra of complex **3.**

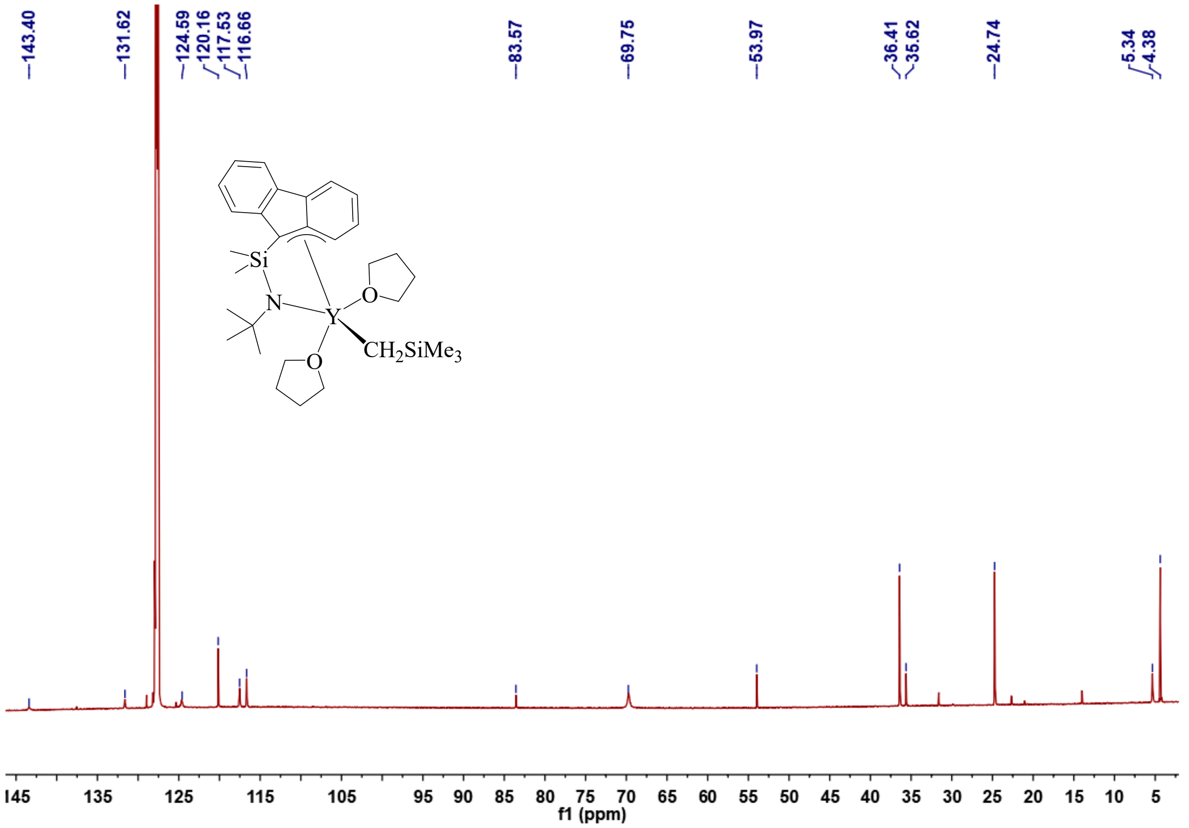

Figure S8. ^{13}C NMR spectra of complex **3.**

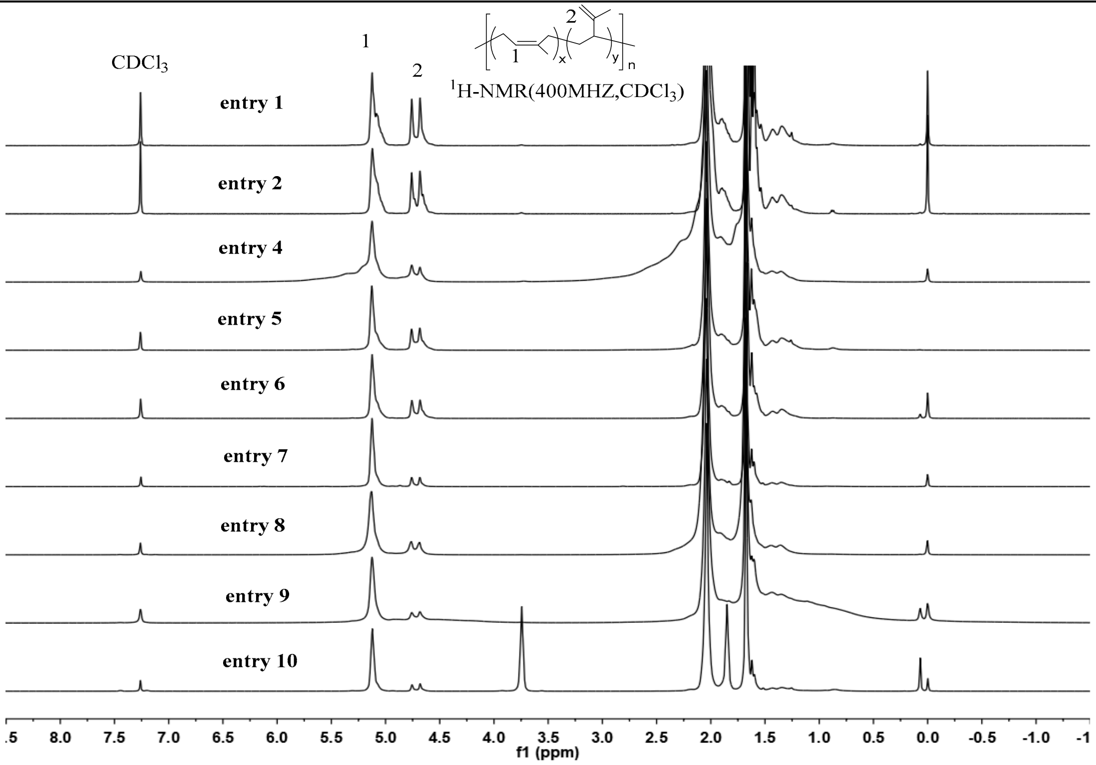

Figure S9. ^{1}H NMR spectra of PIPs in Table 2.

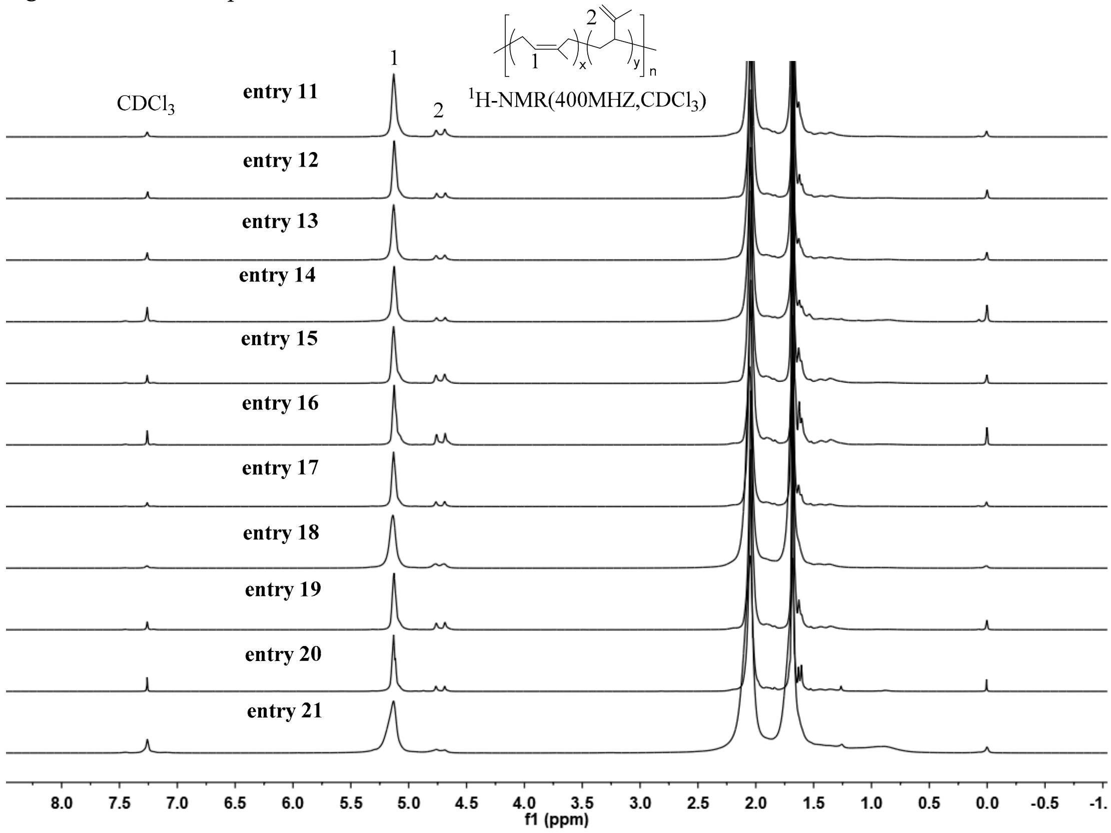

Figure S10. ^{1}H NMR spectra of PIPs in Table 2.

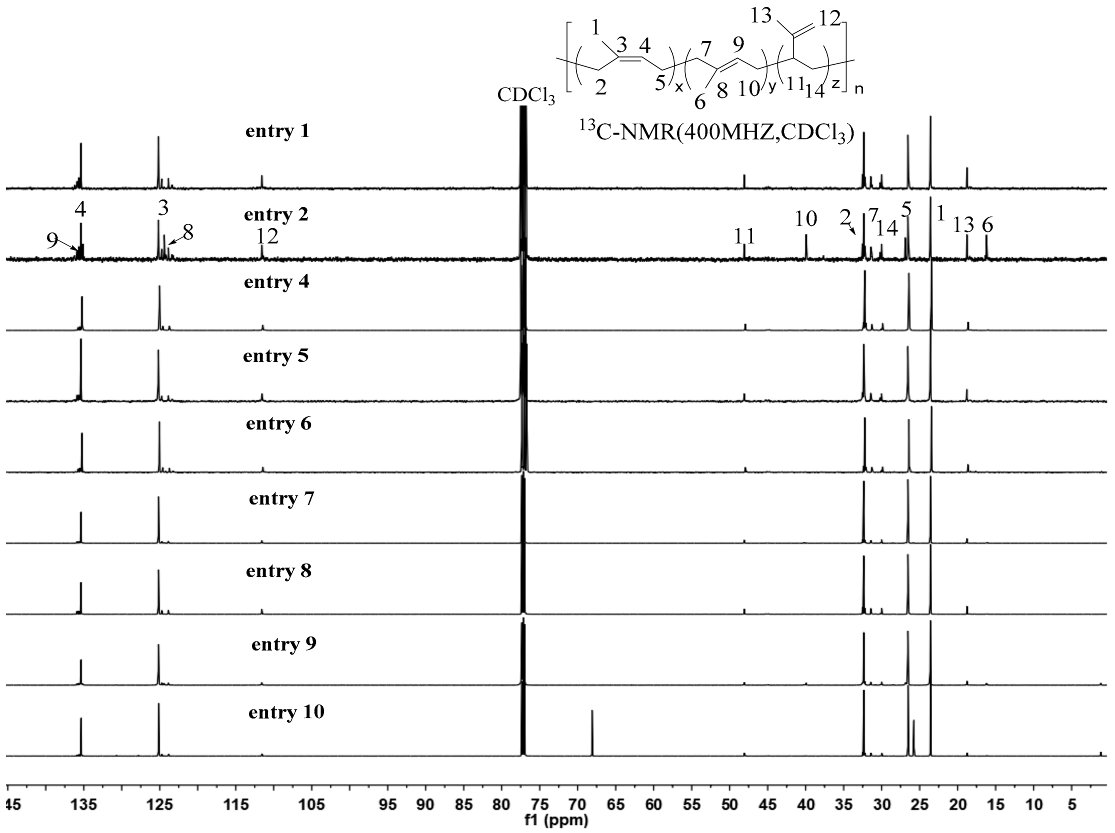

Figure S11. ^{13}C NMR spectra of PIPs in Table 2.

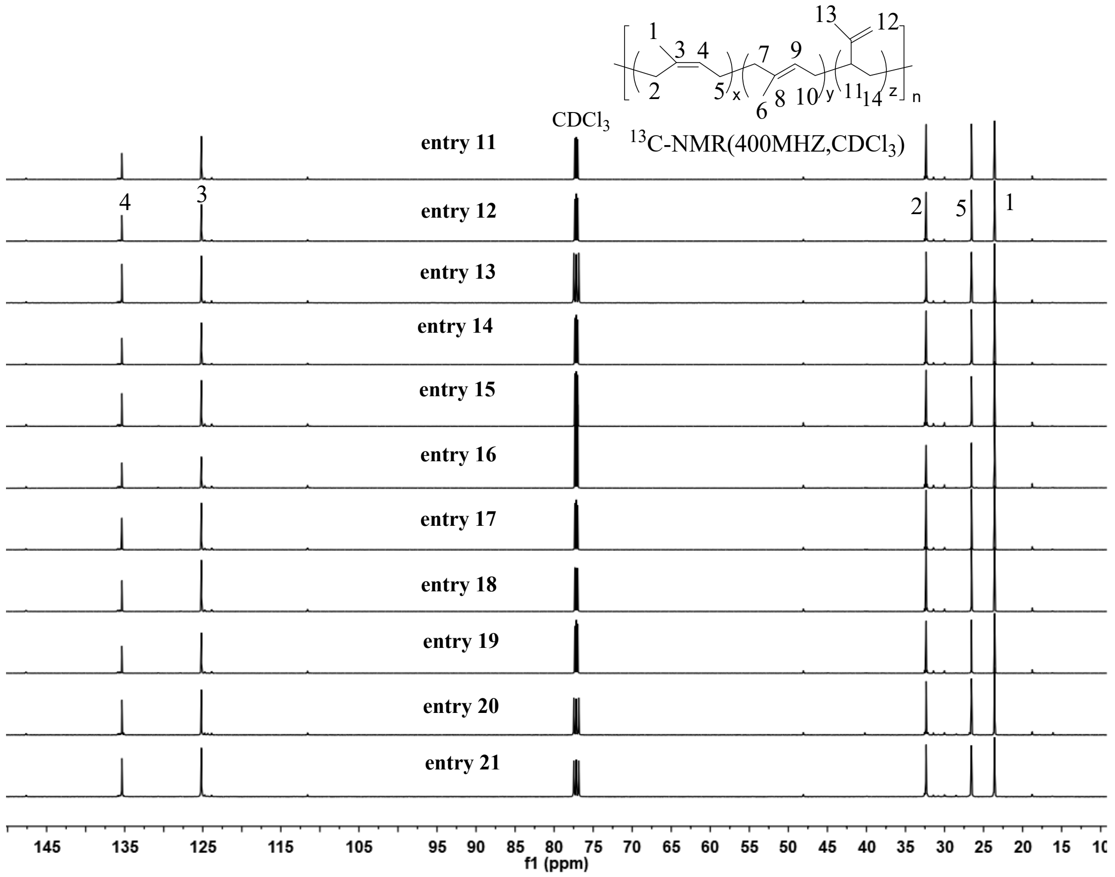

Figure S12. ^{13}C NMR spectra of PIPs in Table 2.

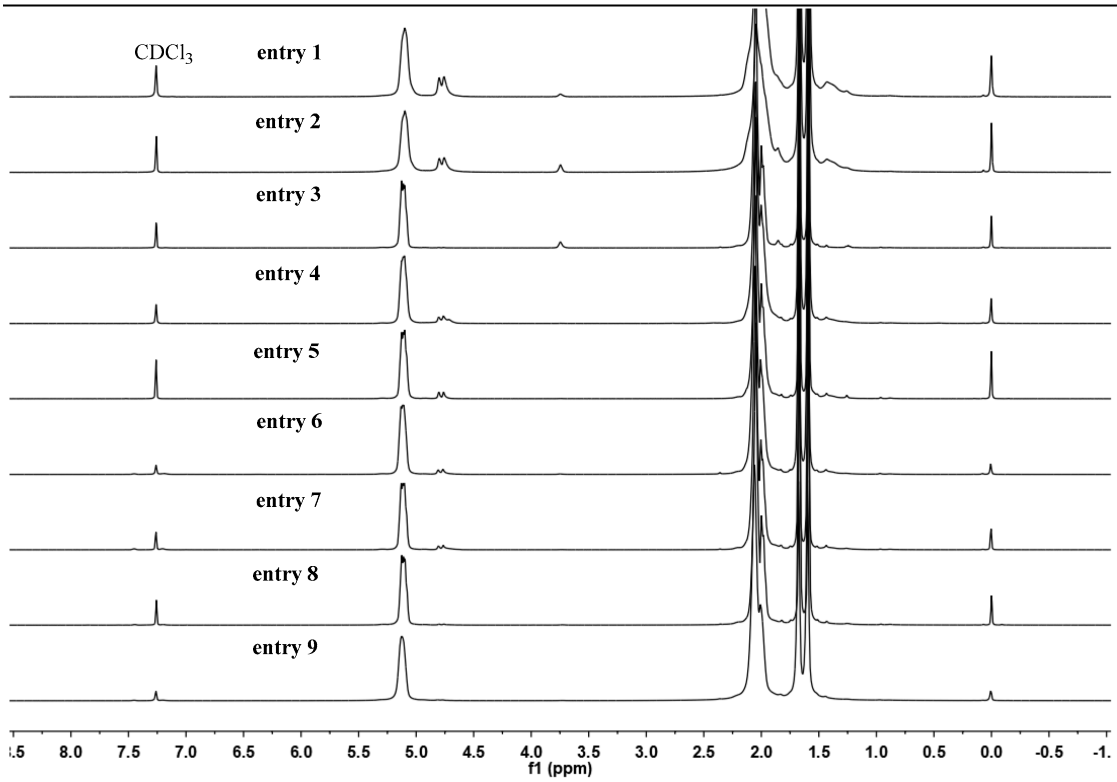

Figure S13. ^{1}H NMR spectra of PMYs in Table 3.

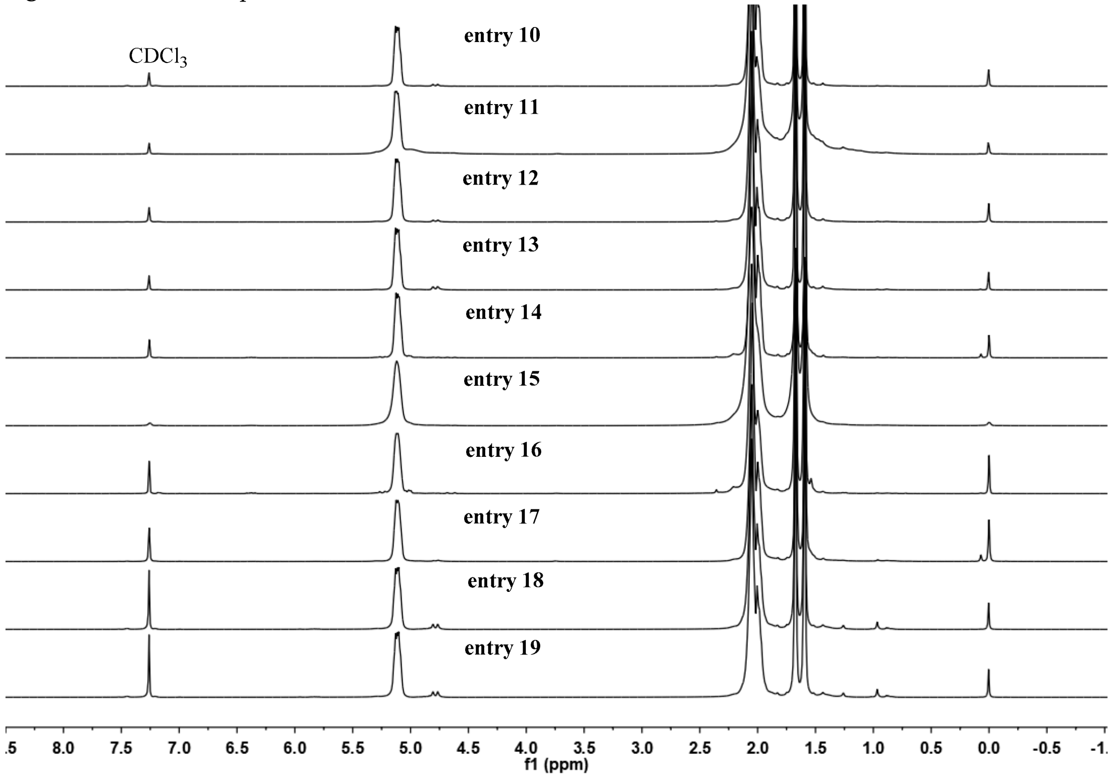

Figure S14. ^{1}H NMR spectra of PMYs in Table 3.

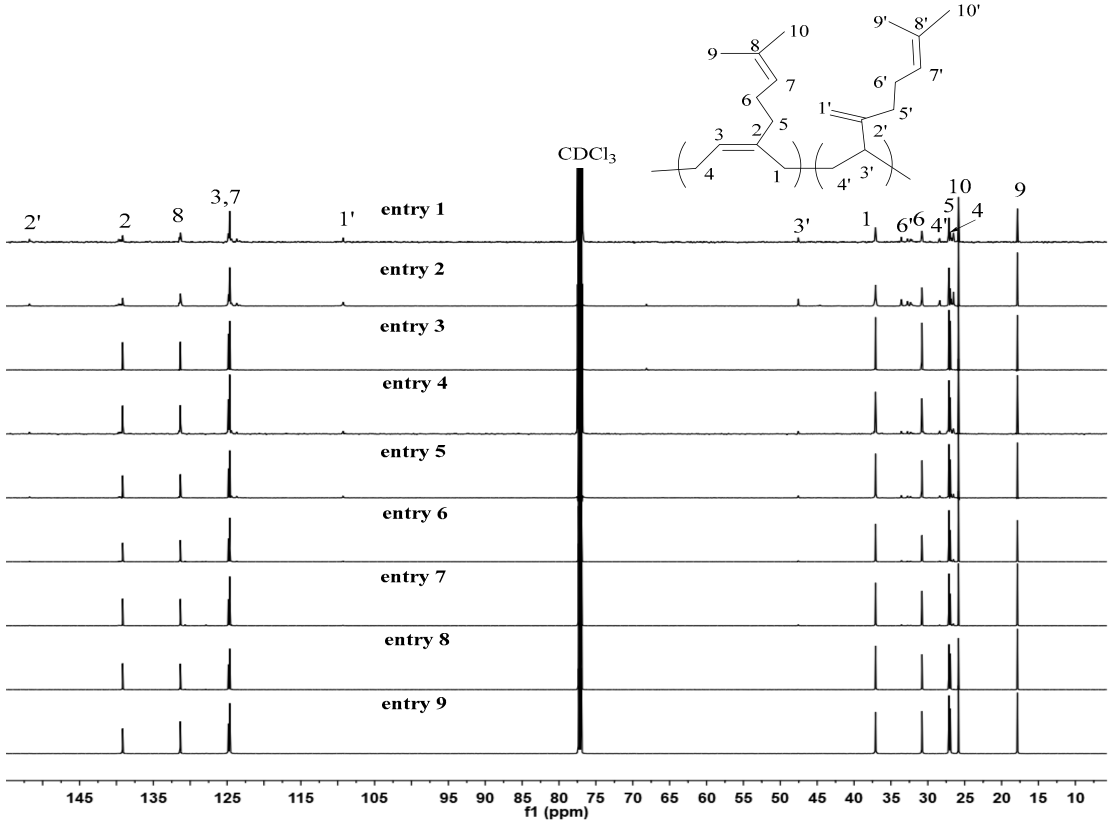

Figure S15. ^{13}C NMR spectra of PMYs in Table 3.

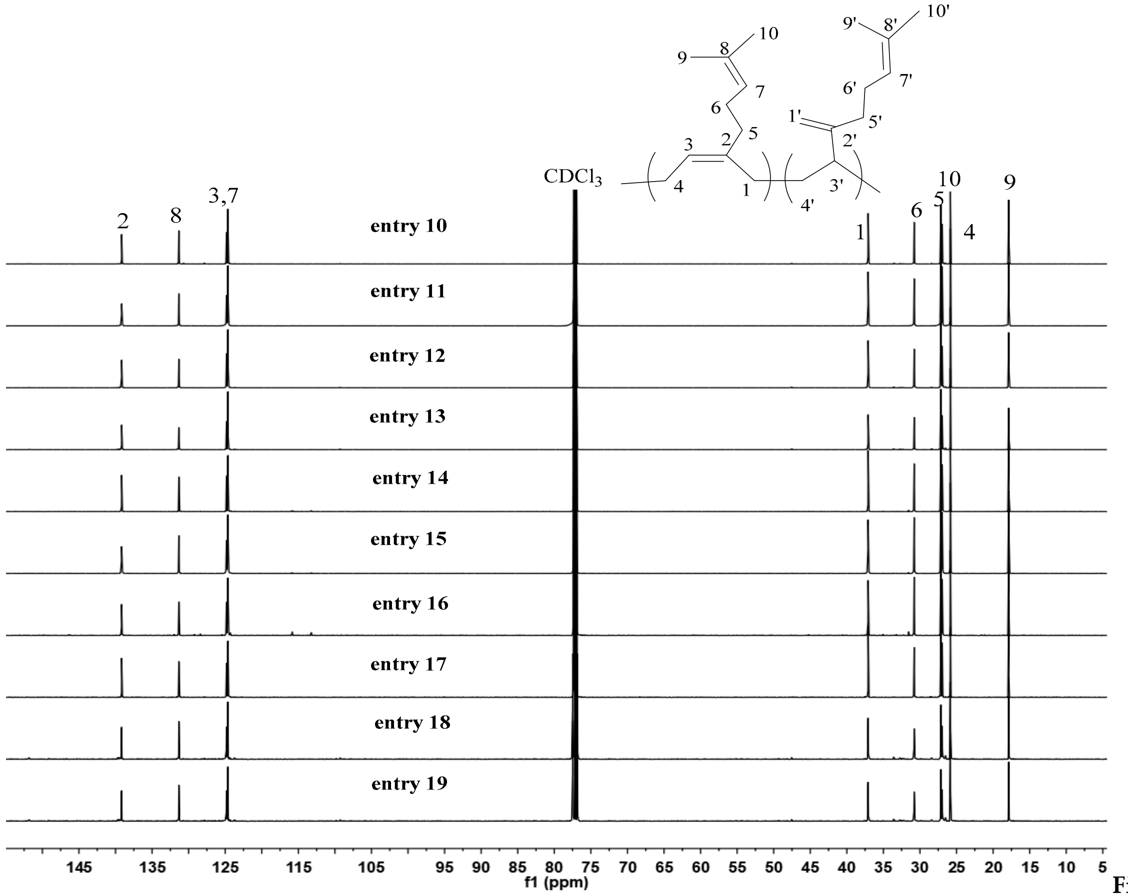

Figure S16. ^{13}C NMR spectra of PMYs in Table 3.

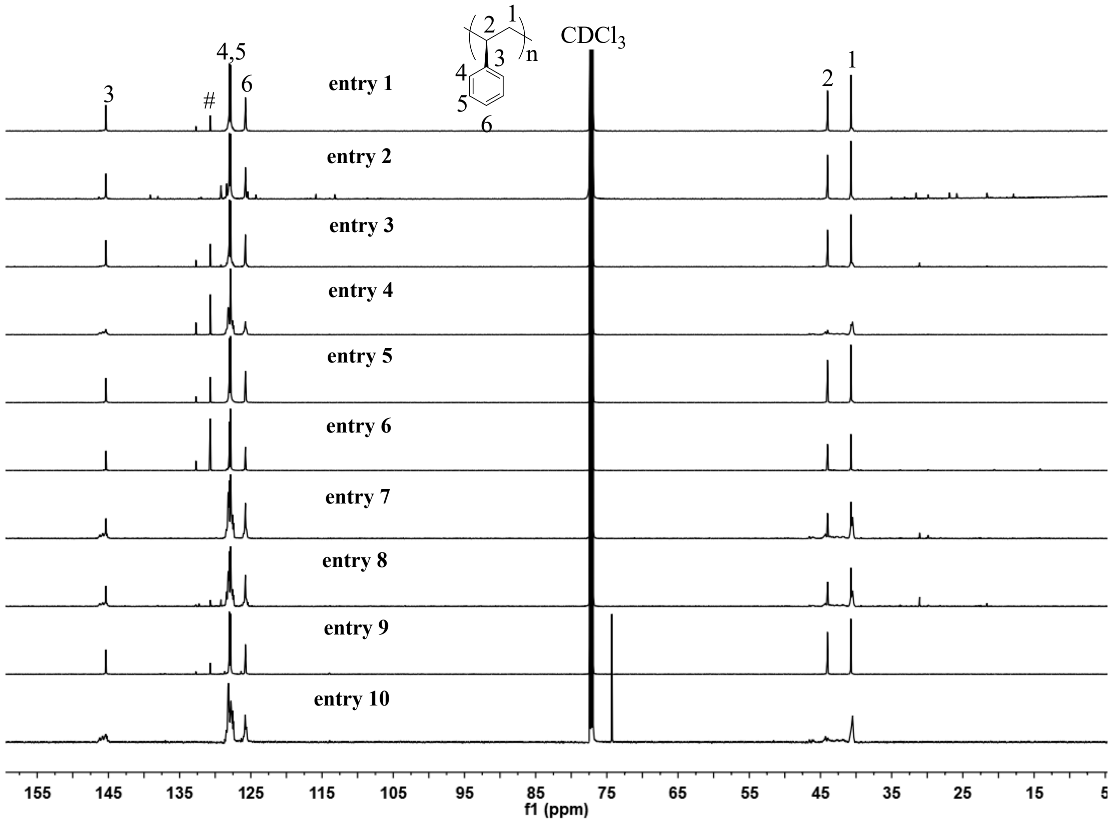

Figure S17. ^{13}C NMR spectra of PSTs in Table 4.

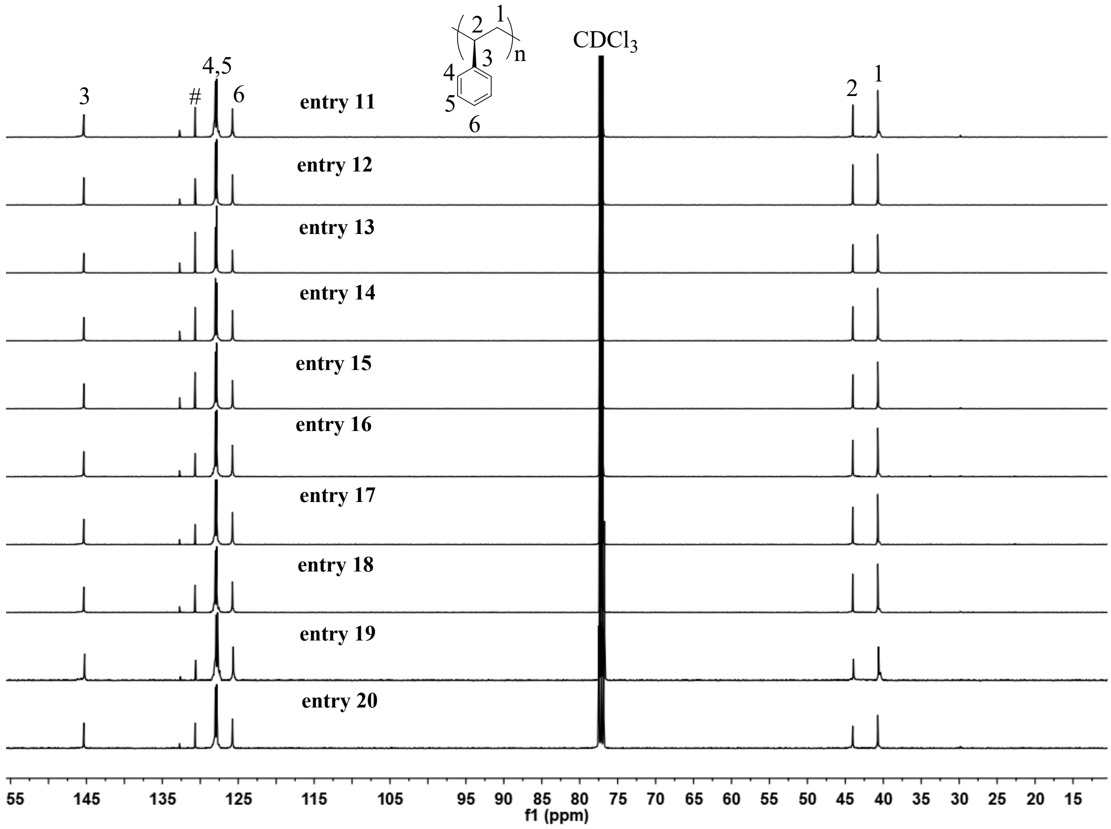

Figure S18. ^{13}C NMR spectra of PSTs in Table 4.

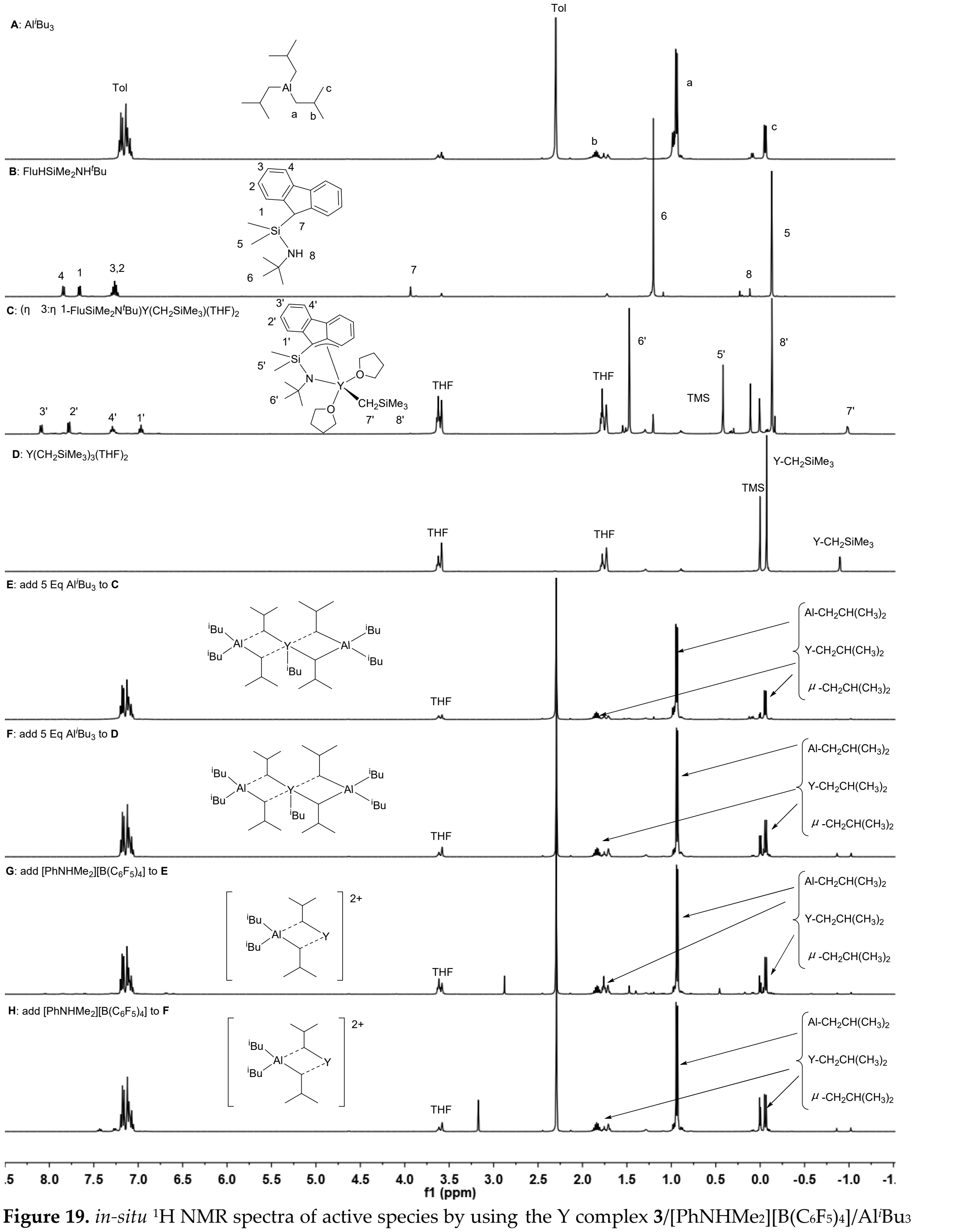

Figure 19. *in-situ* ^{1}H NMR spectra of active species by using the Y complex **3**/[PhNHMe$_2$][B(C$_6$F$_5$)$_4$]/Al^{i}Bu$_3$ ternary system in THF-*d8* at 25 °C.

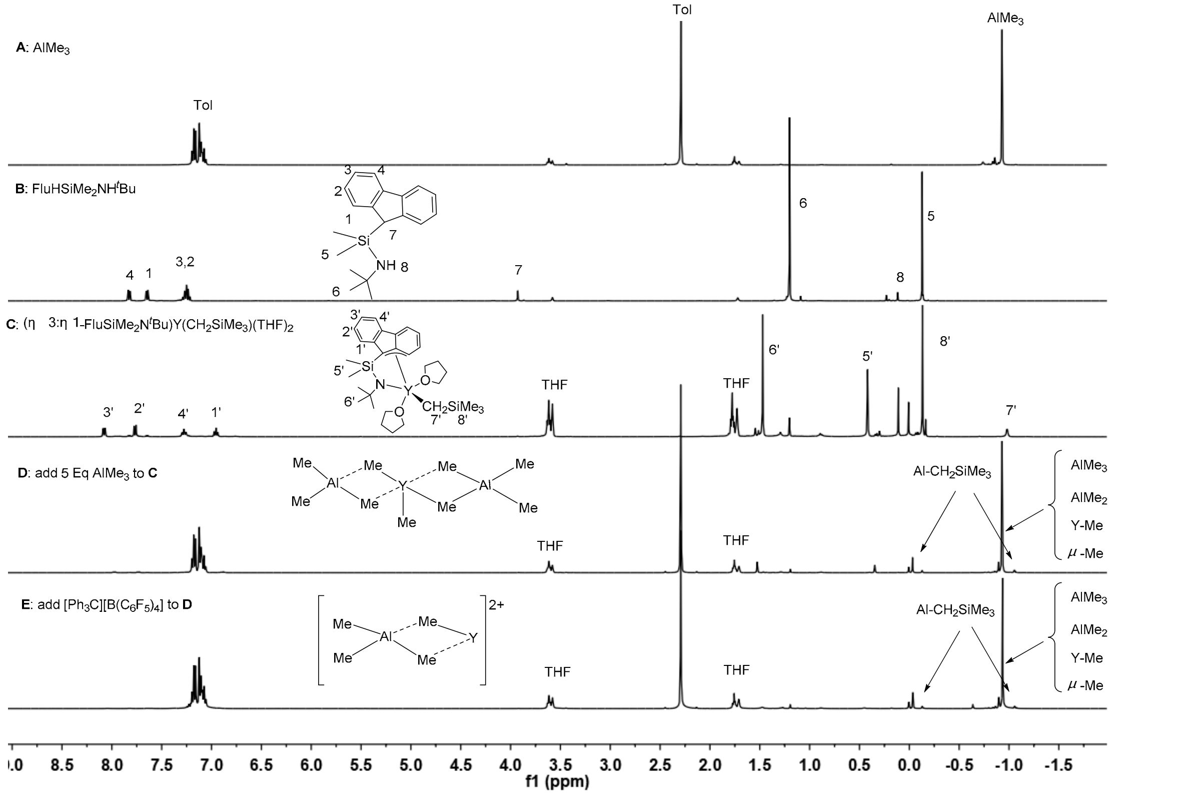

Figure 20. *in-situ* ^{1}H NMR spectra of active species by using the Y complex **3**/[Ph$_3$C][B(C$_6$F$_5$)$_4$]/AlMe$_3$ ternary system in THF-*d8* at 25 °C.

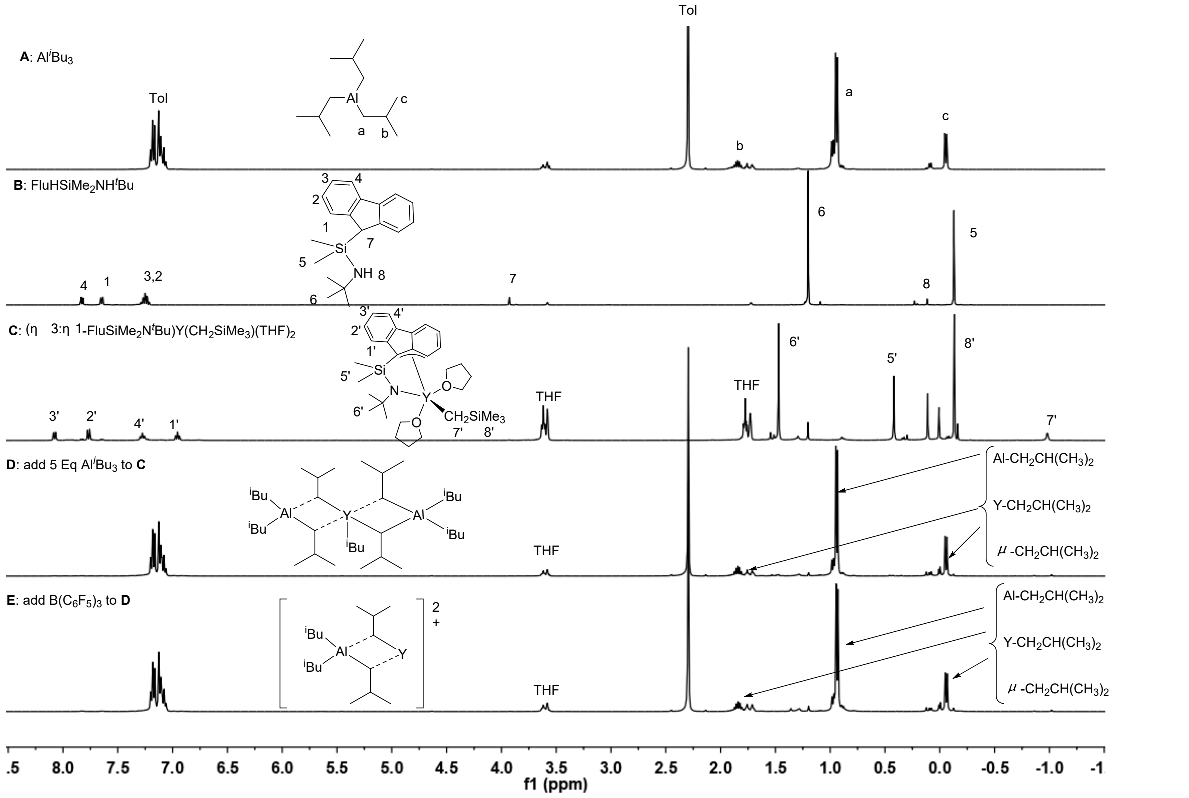

Figure 21. *in-situ* ^{1}H NMR spectra of active species by using the Y complex **3**/B(C$_6$F$_5$)$_3$/Al^{i}Bu$_3$ ternary system in THF-*d8* at 25 °C.

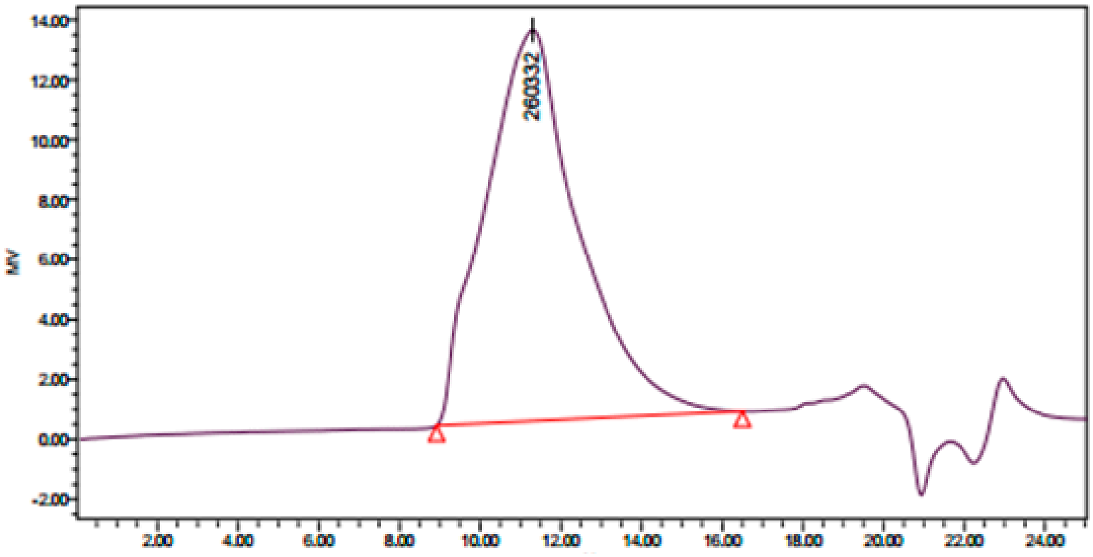

GPC Results

	Dist Name	Elution Volume (ml)	Retention Time (min)	Adjusted RT (min)	Mn	Mw	MP	Mz	Mz+1	Mz/Mw
1		11.308	11.308	11.308	141774	457571	260332	1134678	1850706	2.479787

Figure S22. GPC profiles of the PIPs by the **3**/$Al^{i}Bu_3$/$[Ph_3C][B(C_6F_5)_4]$ systems in Table 2, entry 1.

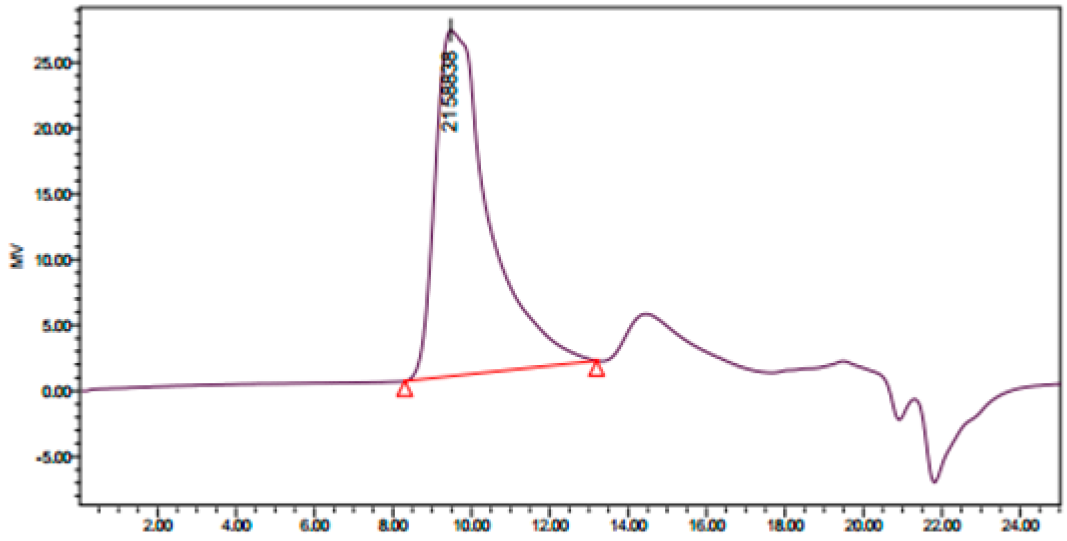

GPC Results

	Dist Name	Elution Volume (ml)	Retention Time (min)	Adjusted RT (min)	Mn	Mw	MP	Mz	Mz+1	Mz/Mw
1		9.476	9.476	9.476	720363	1666298	2158838	2666201	3598692	1.600075

Figure S23. GPC profiles of the PIPs by the **3**/$Al^{i}Bu_3$/$[PhNHMe_2][B(C_6F_5)_4]$ systems in Table 2, entry 2.

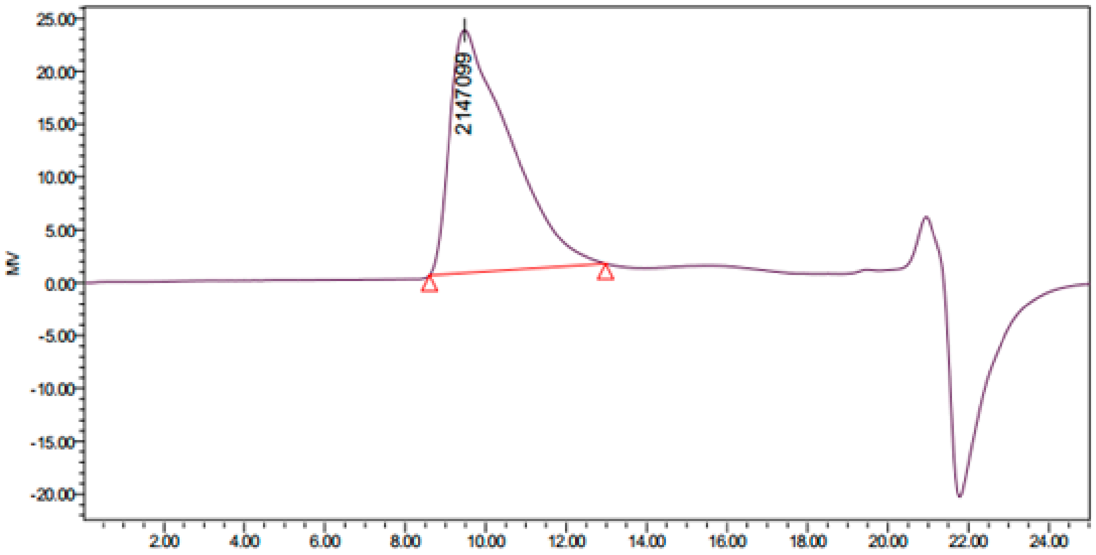

GPC Results

	Dist Name	Elution Volume (ml)	Retention Time (min)	Adjusted RT (min)	Mn	Mw	MP	Mz	Mz+1	Mz/Mw
1		9.480	9.480	9.480	654398	1476846	2147099	2422693	3191936	1.640451

Figure S24. GPC profiles of the PIPs by the **1**/Al^{i}Bu$_3$/[Ph$_3$C][B(C$_6$F$_5$)$_4$] systems in Table 2, entry 4.

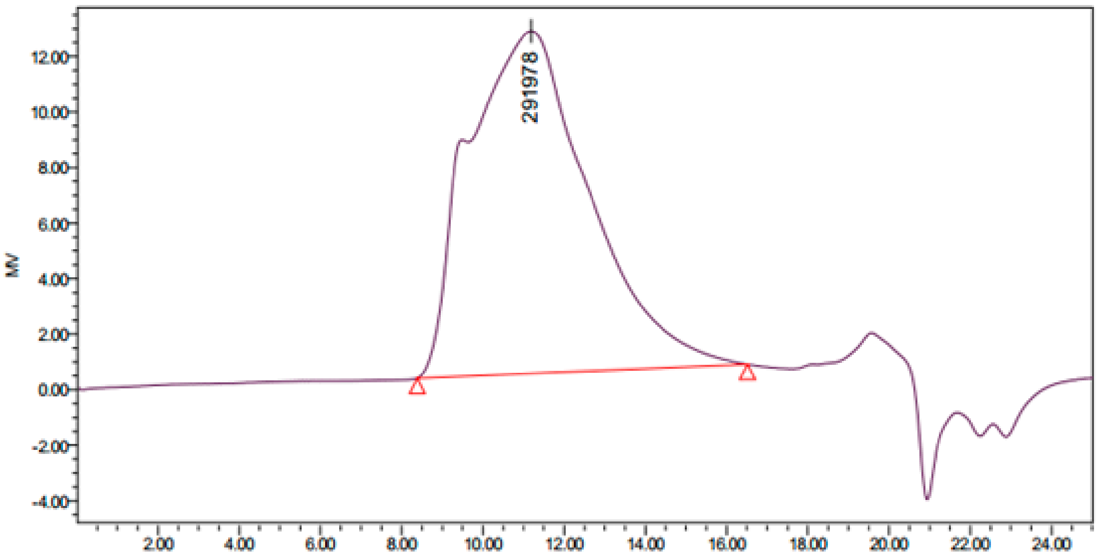

GPC Results

	Dist Name	Elution Volume (ml)	Retention Time (min)	Adjusted RT (min)	Mn	Mw	MP	Mz	Mz+1	Mz/Mw
1		11.190	11.190	11.190	136596	695644	291978	2116919	3535482	3.043106

Figure S25. GPC profiles of the PIPs by the **2**/Al^{i}Bu$_3$/[Ph$_3$C][B(C$_6$F$_5$)$_4$] systems in Table 2, entry 5.

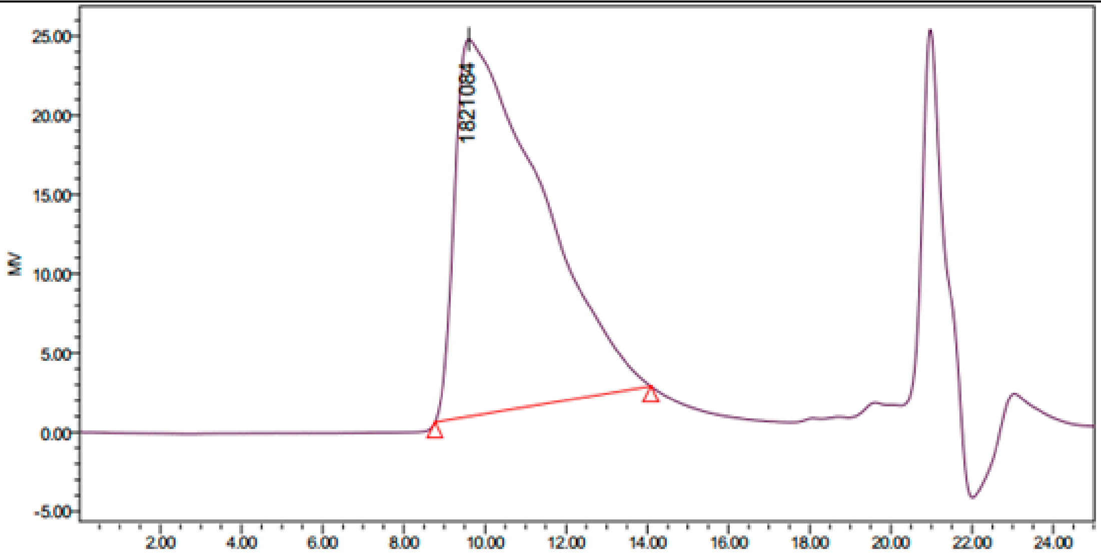

GPC Results

	Dist Name	Elution Volume (ml)	Retention Time (min)	Adjusted RT (min)	Mn	Mw	MP	Mz	Mz+1	Mz/Mw
1		9.602	9.602	9.602	292665	927236	1821084	1796851	2446744	1.937858

Figure S26. GPC profiles of the PIPs by the **3**/Al^{i}Bu$_3$/[Ph$_3$C][B(C$_6$F$_5$)$_4$] systems in Table 2, entry 6.

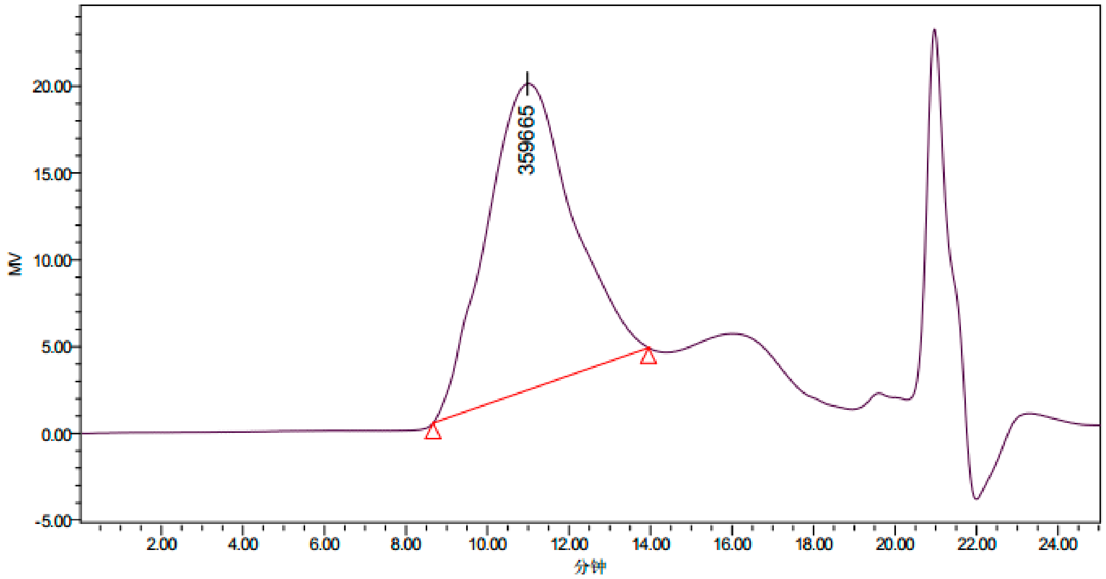

GPC Results

	Dist Name	Elution Volume (ml)	Retention Time (min)	Adjusted RT (min)	Mn	Mw	MP	Mz	Mz+1	Mz/Mw
1		10.983	10.983	10.983	226201	576342	359665	1407297	2494628	2.441776

Figure S27. GPC profiles of the PIPs by the **1**/AlMe$_3$/[Ph$_3$C][B(C$_6$F$_5$)$_4$] systems in Table 2, entry 7.

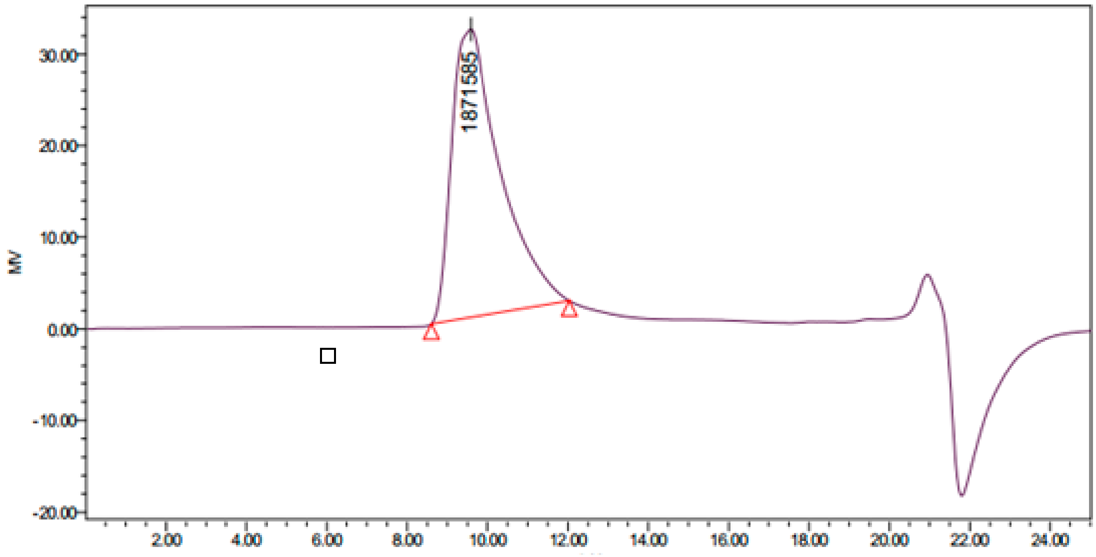

GPC Results

	Dist Name	Elution Volume (ml)	Retention Time (min)	Adjusted RT (min)	Mn	Mw	MP	Mz	Mz+1	Mz/Mw
1		9.582	9.582	9.582	976621	1729378	1871585	2510750	3176221	1.451822

Figure S28. GPC profiles of the PIPs by the **1**/$AlEt_3$/$[Ph_3C][B(C_6F_5)_4]$ systems in Table 2, entry 8.

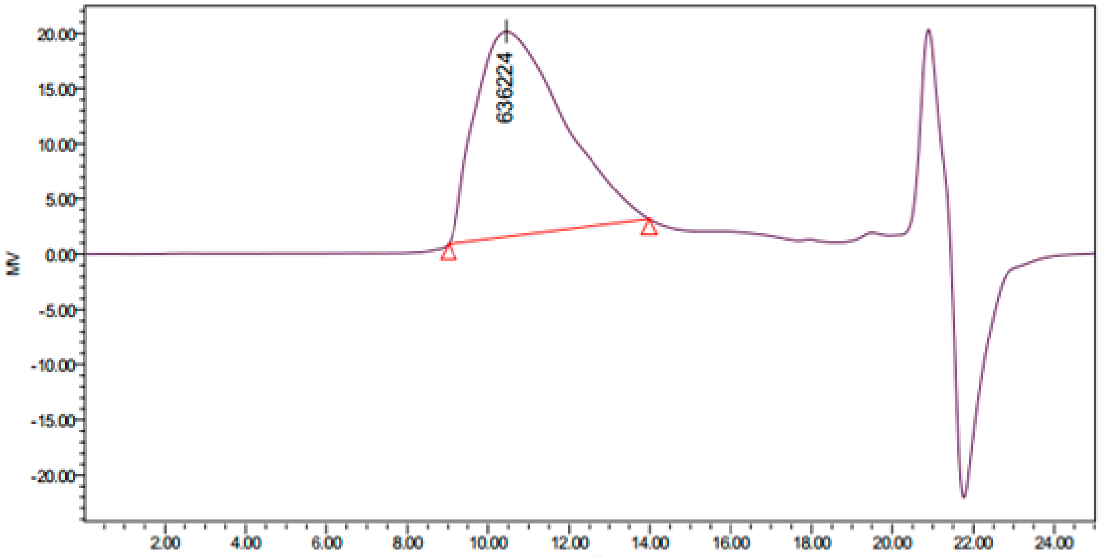

GPC Results

	Dist Name	Elution Volume (ml)	Retention Time (min)	Adjusted RT (min)	Mn	Mw	MP	Mz	Mz+1	Mz/Mw
1		10.455	10.455	10.455	244163	629393	636224	1220056	1746801	1.938465

Figure S29. GPC profiles of the PIPs by the **1**/$AlMe_3$/$[Ph_3C][B(C_6F_5)_4]$ systems in Table 2, entry 9.

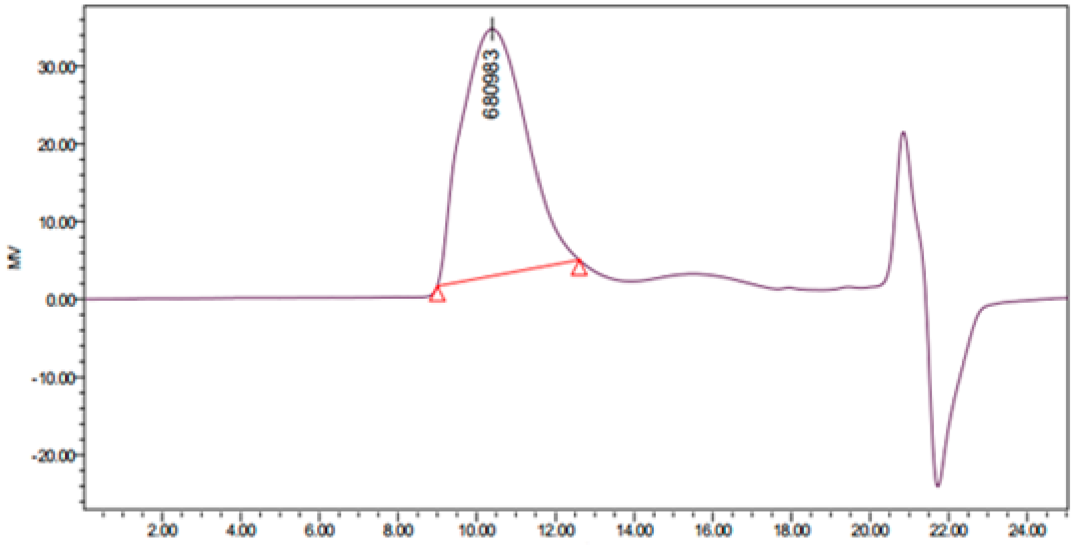

GPC Results

	Dist Name	Elution Volume (ml)	Retention Time (min)	Adjusted RT (min)	Mn	Mw	MP	Mz	Mz+1	Mz/Mw
1		10.396	10.396	10.396	477057	854239	680983	1388073	1899254	1.624923

Figure S30. GPC profiles of the PIPs by the **1**/$AlMe_3$/$[Ph_3C][B(C_6F_5)_4]$ systems in Table 2, entry 10.

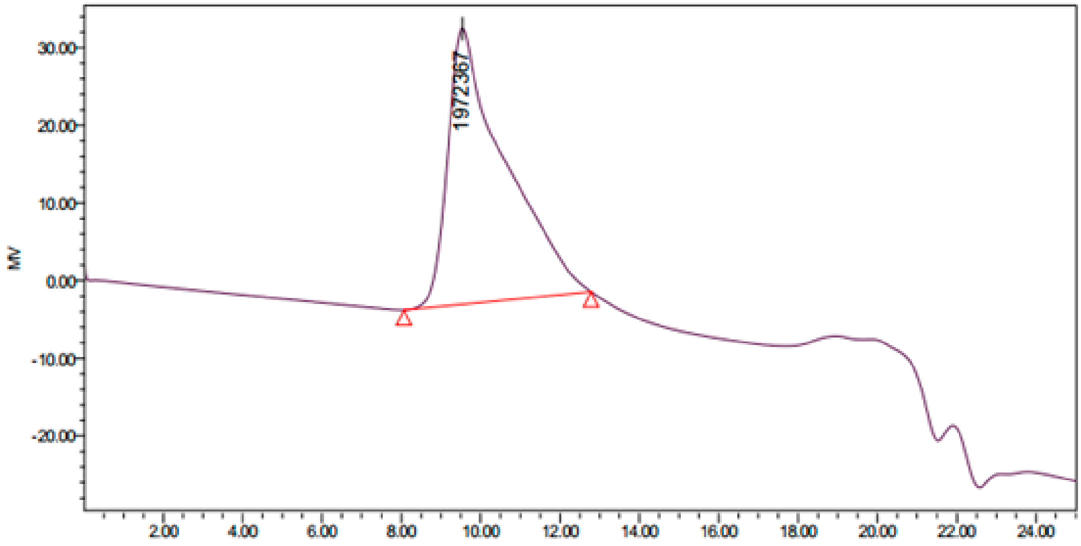

GPC Results

	Dist Name	Elution Volume (ml)	Retention Time (min)	Adjusted RT (min)	Mn	Mw	MP	Mz	Mz+1	Mz/Mw
1		9.543	9.543	9.543	585844	1397445	1972367	2446919	3513702	1.750995

Figure S31. GPC profiles of the PIPs by the **1**/$AlMe_3$/$[Ph_3C][B(C_6F_5)_4]$ systems in Table 2, entry 11.

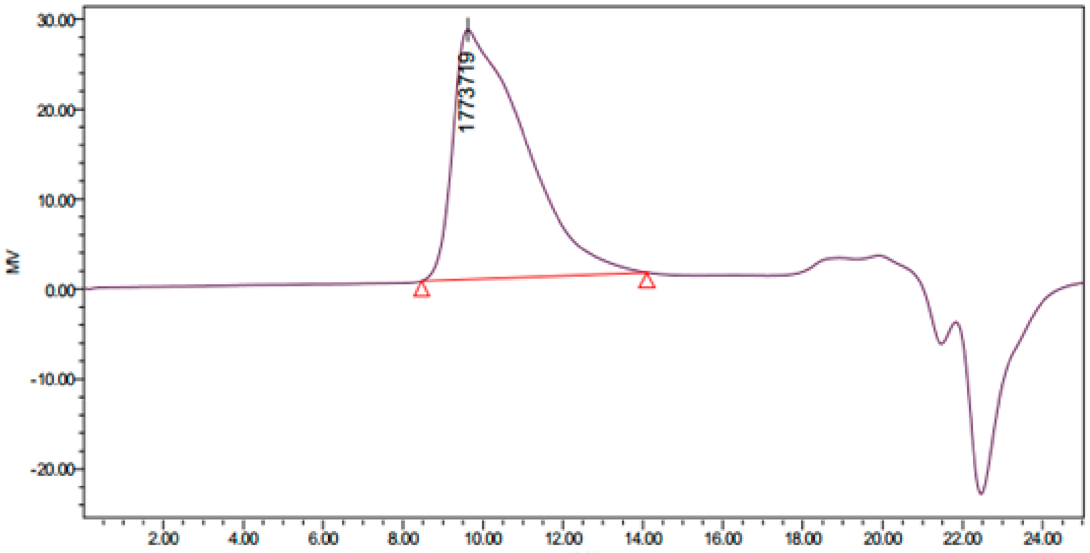

GPC Results

	Dist Name	Elution Volume (ml)	Retention Time (min)	Adjusted RT (min)	Mn	Mw	MP	Mz	Mz+1	Mz/Mw
1		9.622	9.622	9.622	415911	111017(	1773719	2028511	2908006	1.827206

Figure S32. GPC profiles of the PIPs by the **1**/$AlMe_3$/$[Ph_3C][B(C_6F_5)_4]$ systems in Table 2, entry 12.

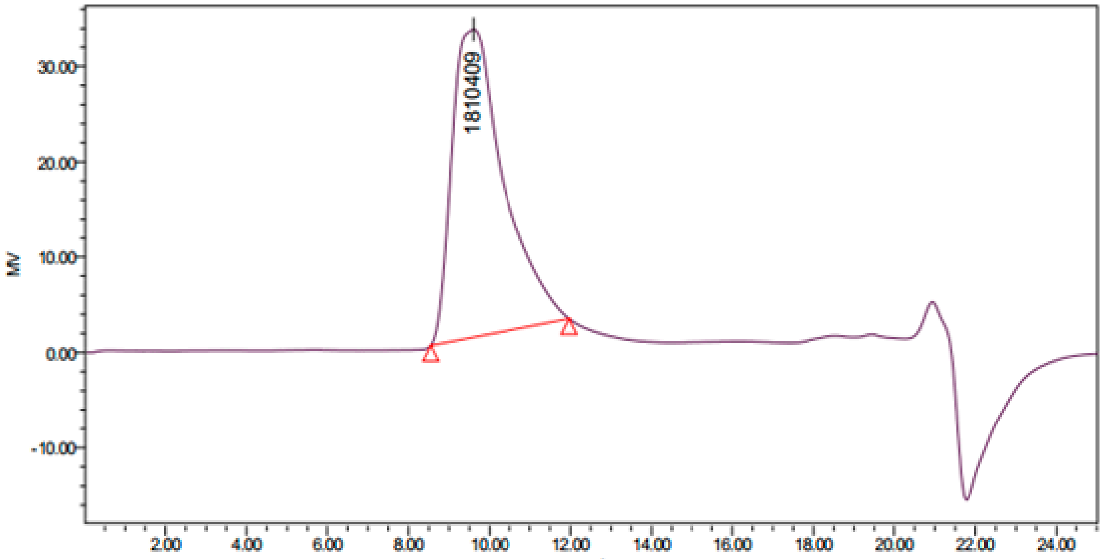

GPC Results

	Dist Name	Elution Volume (ml)	Retention Time (min)	Adjusted RT (min)	Mn	Mw	MP	Mz	Mz+1	Mz/Mw
1		9.607	9.607	9.607	1001618	1800236	1810409	2667690	3430635	1.481856

Figure S33. GPC profiles of the PIPs by the **1**/$AlMe_3$/$[Ph_3C][B(C_6F_5)_4]$ systems in Table 2, entry 13.

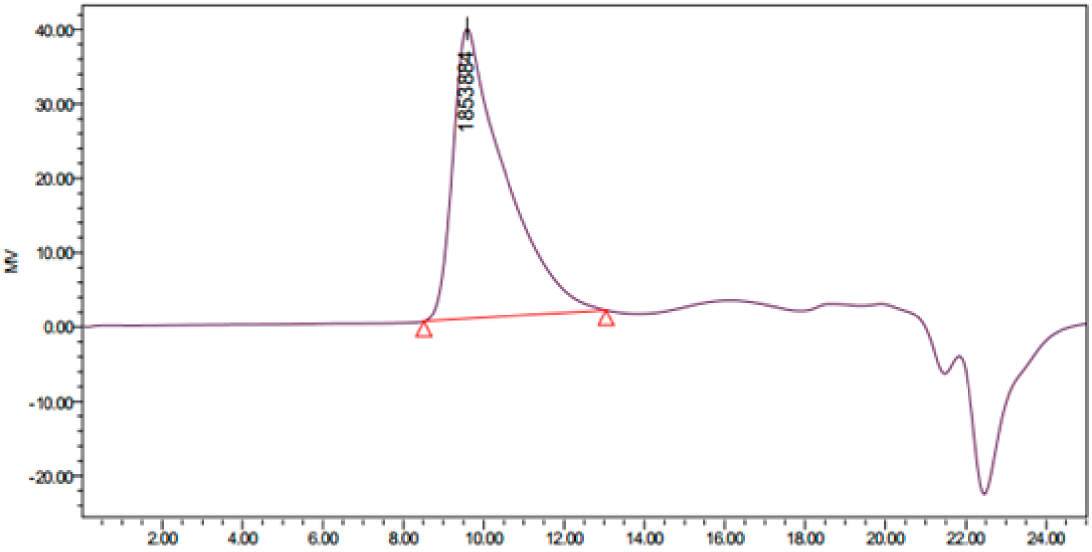

GPC Results

	Dist Name	Elution Volume (ml)	Retention Time (min)	Adjusted RT (min)	Mn	Mw	MP	Mz	Mz+1	Mz/Mw
1		9.589	9.589	9.589	648776	1362499	1853884	2137755	2831547	1.568996

Figure S34. GPC profiles of the PIPs by the **1**/$AlMe_3$/$[Ph_3C][B(C_6F_5)_4]$ systems in Table 2, entry 14.

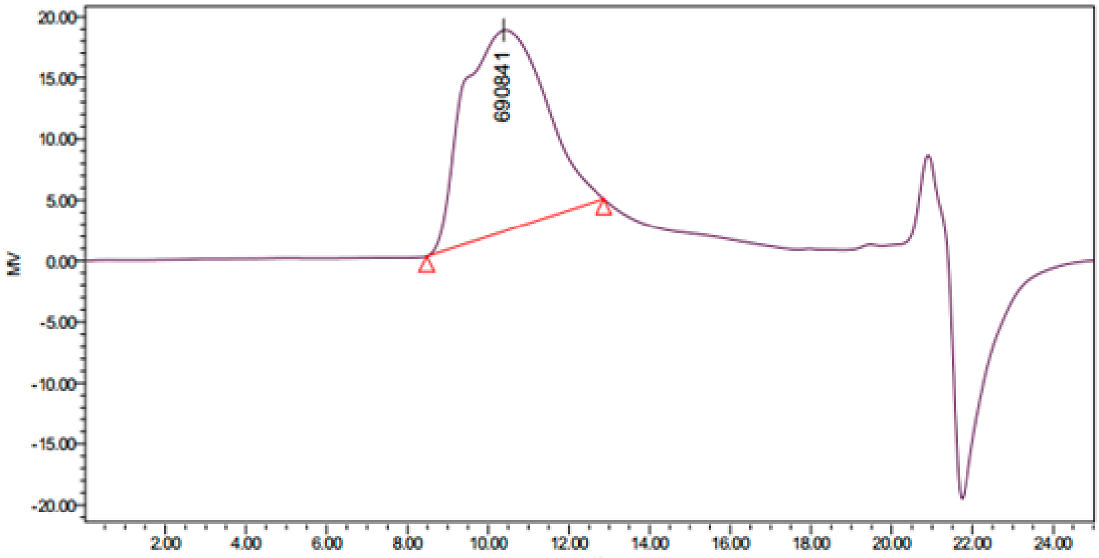

GPC Results

	Dist Name	Elution Volume (ml)	Retention Time (min)	Adjusted RT (min)	Mn	Mw	MP	Mz	Mz+1	Mz/Mw
1		10.383	10.383	10.383	447622	1054386	690841	2069251	3038548	1.962517

Figure S35. GPC profiles of the PIPs by the **1**/$AlMe_3$/$[Ph_3C][B(C_6F_5)_4]$ systems in Table 2, entry 15.

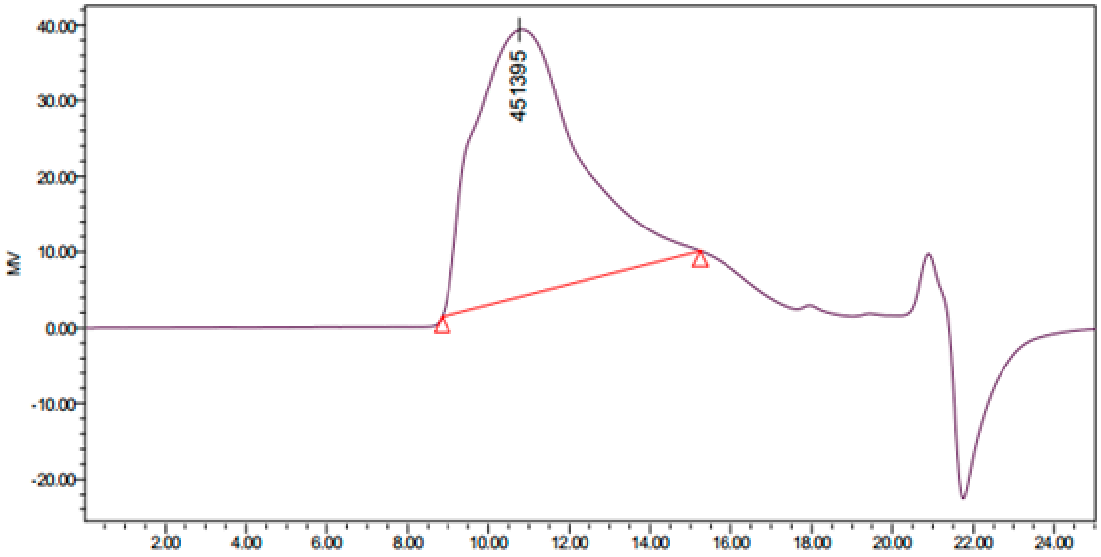

GPC Results

	Dist Name	Elution Volume (ml)	Retention Time (min)	Adjusted RT (min)	Mn	Mw	MP	Mz	Mz+1	Mz/Mw
1		10.767	10.767	10.767	174262	647066	451395	1511543	2263791	2.335997

Figure S36. GPC profiles of the PIPs by the **1**/$AlMe_3$/$[Ph_3C][B(C_6F_5)_4]$ systems in Table 2, entry 16.

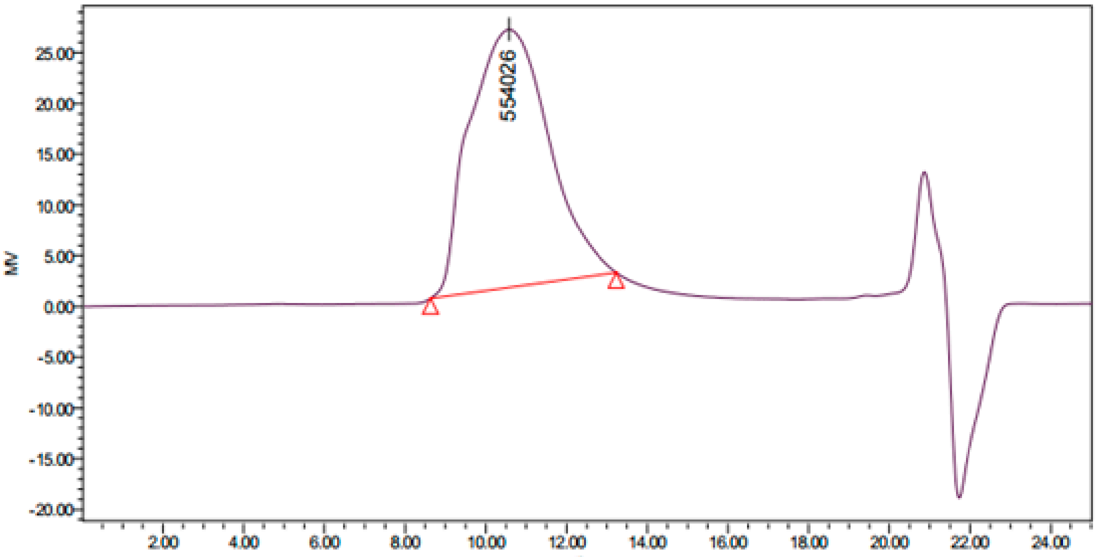

GPC Results

	Dist Name	Elution Volume (ml)	Retention Time (min)	Adjusted RT (min)	Mn	Mw	MP	Mz	Mz+1	Mz/Mw
1		10.579	10.579	10.579	370820	825520	554026	1591248	2379745	1.927571

Figure S37. GPC profiles of the PIPs by the **1**/$AlMe_3$/$[Ph_3C][B(C_6F_5)4]$ systems in Table 2, entry 17.

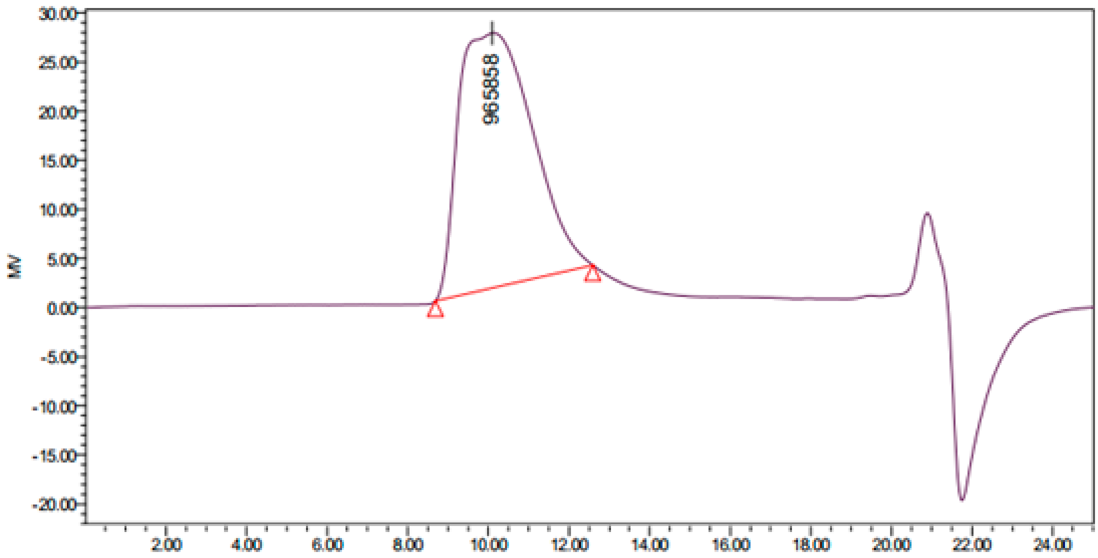

GPC Results

	Dist Name	Elution Volume (ml)	Retention Time (min)	Adjusted RT (min)	Mn	Mw	MP	Mz	Mz+1	Mz/Mw
1		10.100	10.100	10.100	576574	1180143	965858	2002085	2724914	1.696477

Figure S38. GPC profiles of the PIPs by the **1**/$AlMe_3$/$[Ph_3C][B(C_6F_5)_4]$ systems in Table 2, entry 18.

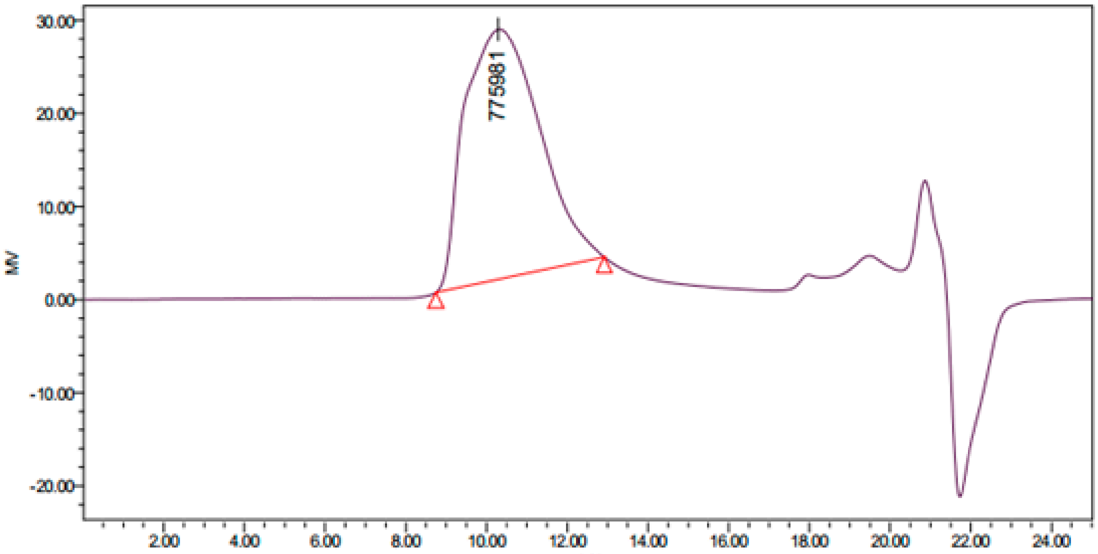

GPC Results

	Dist Name	Elution Volume (ml)	Retention Time (min)	Adjusted RT (min)	Mn	Mw	MP	Mz	Mz+1	Mz/Mw
1		10.283	10.283	10.283	451587	947755	775981	1667180	2324866	1.759083

Figure S39. GPC profiles of the PIPs by the **1**/$AlMe_3$/$[Ph_3C][B(C_6F_5)_4]$ systems in Table 2, entry 19.

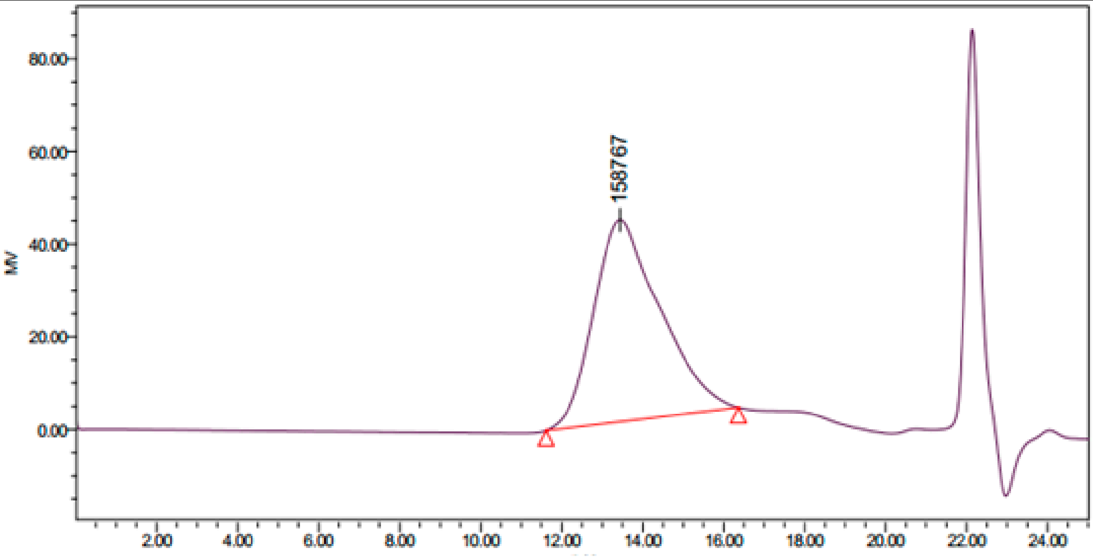

GPC Results

	Dist Name	Elution Volume (ml)	Retention Time (min)	Adjusted RT (min)	Mn	Mw	MP	Mz	Mz+1	Mz/Mw
1		13.447	13.447	13.447	85592	186454	158767	405990	772692	2.177425

Figure S40. GPC profiles of the PIPs by the $Sc(CH_2SiCH_3)_3(THF)_2/AlMe_3/[Ph_3C][B(C_6F_5)_4]$ systems in Table 2, entry 20.

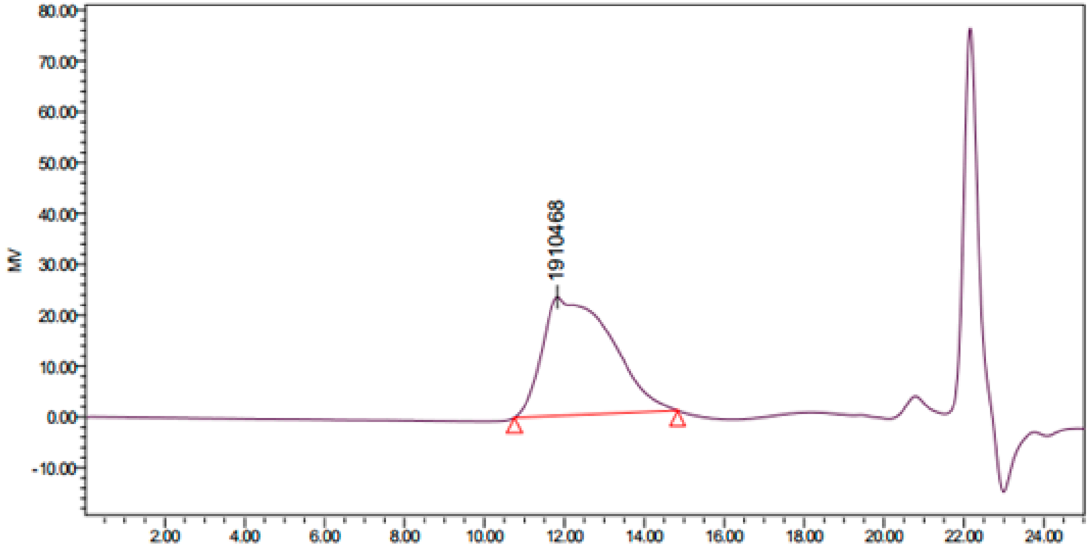

GPC Results

	Dist Name	Elution Volume (ml)	Retention Time (min)	Adjusted RT (min)	Mn	Mw	MP	Mz	Mz+1	Mz/Mw
1		11.828	11.828	11.828	370090	1377847	1910468	3773824	6692025	2.738927

Figure S41. GPC profiles of the PIPs by the $Sc(CH_2SiCH_3)_3(THF)_2/AlMe_3/[Ph_3C][B(C_6F_5)_4]$ systems in Table 2, entry 21.

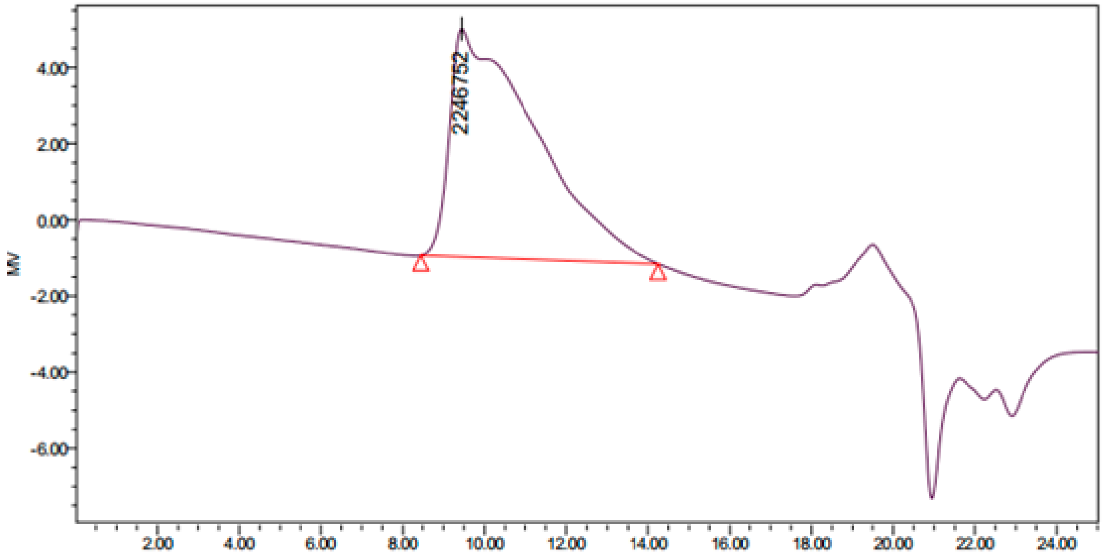

GPC Results

	Dist Name	Elution Volume (ml)	Retention Time (min)	Adjusted RT (min)	Mn	Mw	MP	Mz	Mz+1	Mz/Mw
1		9.447	9.447	9.447	307163	1066229	2246752	2182564	3118471	2.046994

Figure S42. GPC profiles of the PMYs by the **3**/Al^{i}Bu$_3$/[Ph$_3$C][B(C$_6$F$_5$)$_4$] systems in Table 3, entry 1.

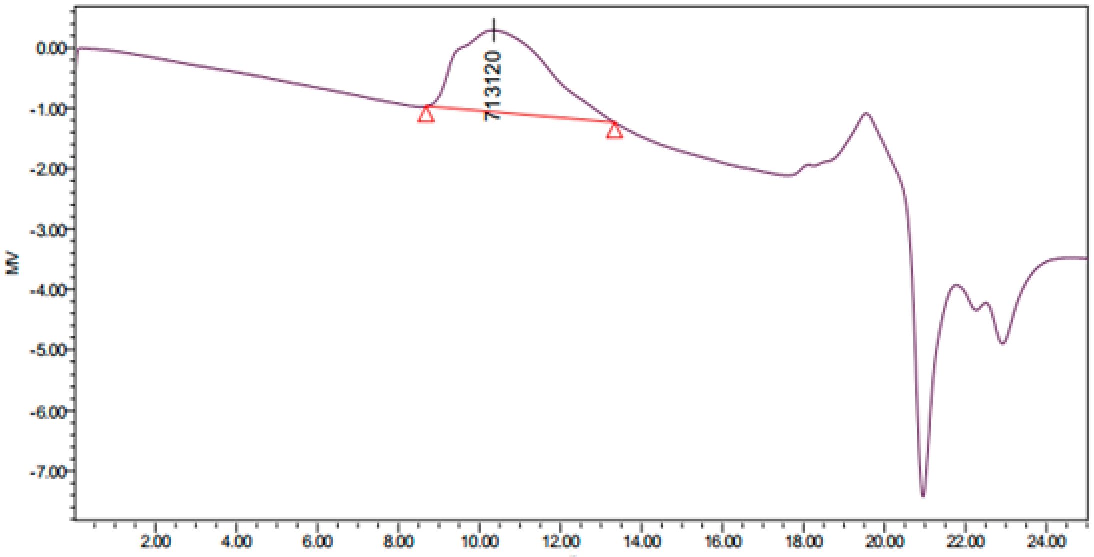

GPC Results

	Dist Name	Elution Volume (ml)	Retention Time (min)	Adjusted RT (min)	Mn	Mw	MP	Mz	Mz+1	Mz/Mw
1		10.356	10.356	10.356	327118	835991	713120	1701270	2503747	2.035035

Figure S43. GPC profiles of the PMYs by the **3**/Al^{i}Bu$_3$/[PhNHMe$_2$][B(C$_6$F$_5$)$_4$] systems in Table 3, entry 2.

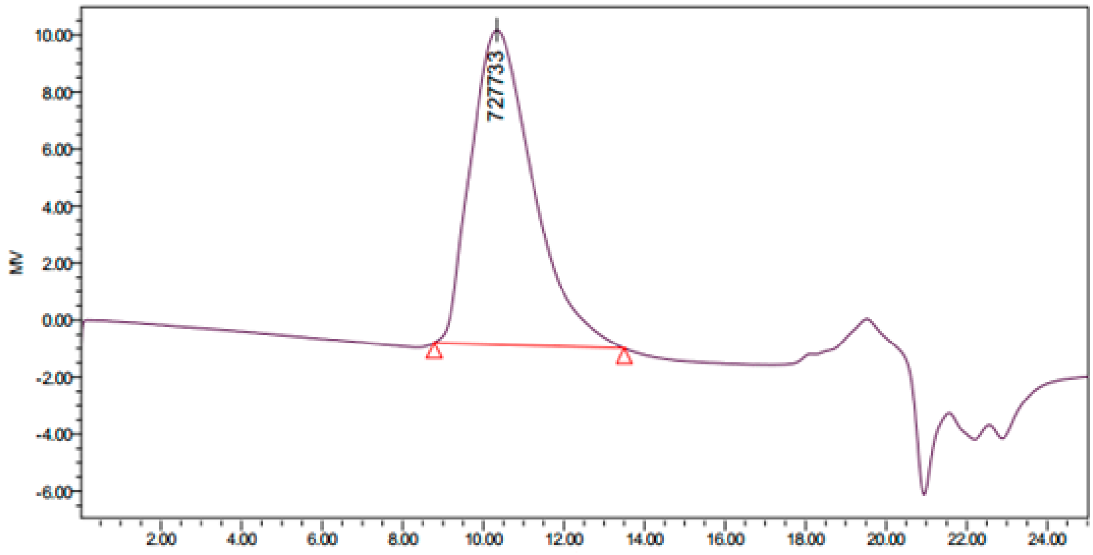

GPC Results

	Dist Name	Elution Volume (ml)	Retention Time (min)	Adjusted RT (min)	Mn	Mw	MP	Mz	Mz+1	Mz/Mw
1		10.338	10.338	10.338	403777	781288	727733	1292257	1848577	1.654008

Figure S44. GPC profiles of the PMYs by the **3**/Al^{i}Bu$_3$/B(C$_6$F$_5$)$_3$ systems in Table 3, entry 3.

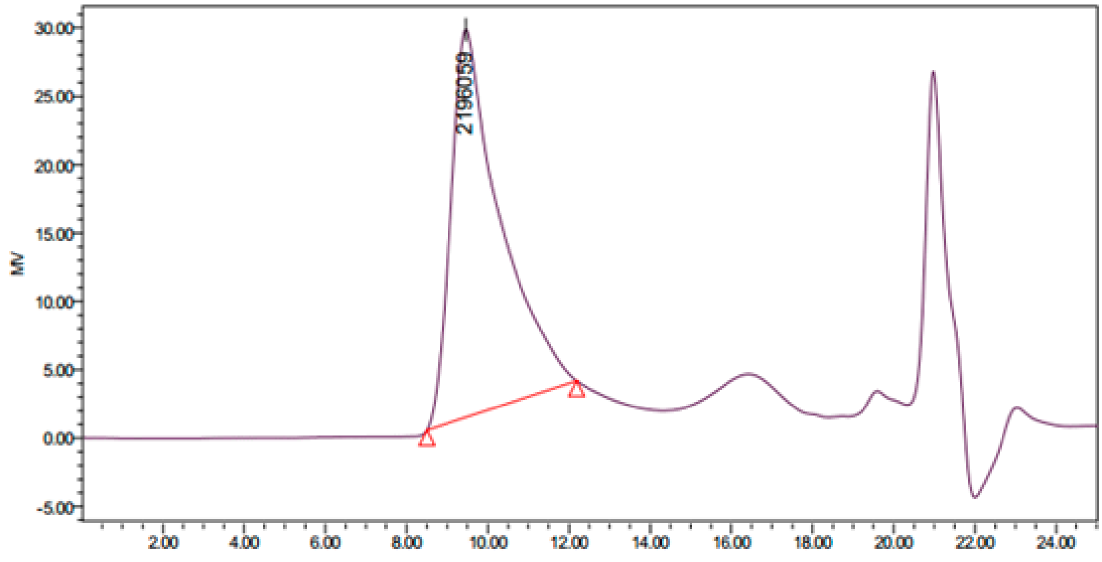

GPC Results

	Dist Name	Elution Volume (ml)	Retention Time (min)	Adjusted RT (min)	Mn	Mw	MP	Mz	Mz+1	Mz/Mw
1		9.464	9.464	9.464	883546	1743792	2196059	2692090	3536448	1.543813

Figure S45. GPC profiles of the PMYs by the **1**/Al^{i}Bu$_3$/B(C$_6$F$_5$)$_3$ systems in Table 3, entry 4.

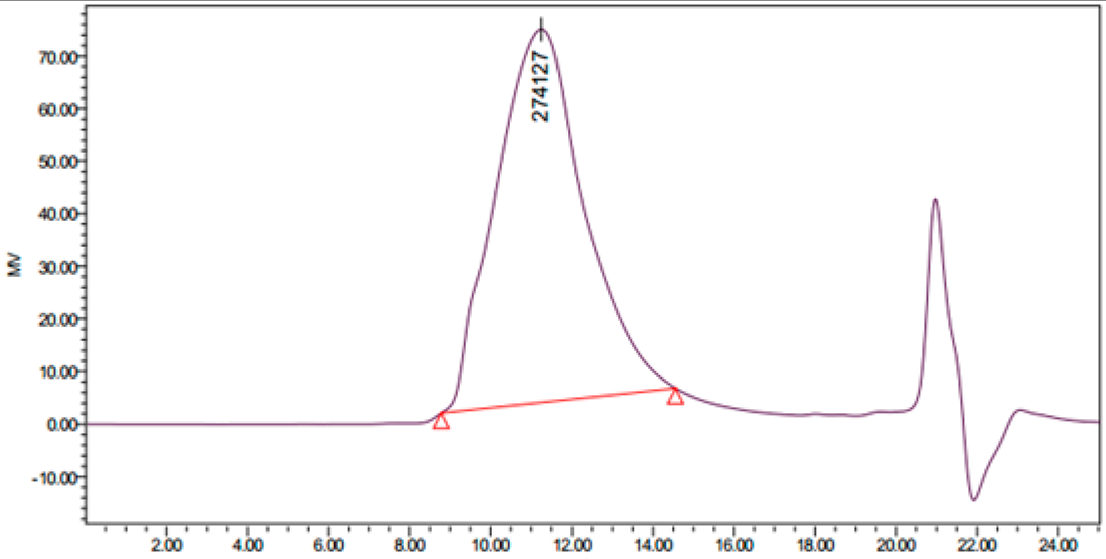

GPC Results

	Dist Name	Elution Volume (ml)	Retention Time (min)	Adjusted RT (min)	Mn	Mw	MP	Mz	Mz+1	Mz/Mw
1		11.255	11.255	11.255	184871	464521	274127	1063503	1787967	2.289462

Figure S46. GPC profiles of the PMYs by the **2**/$Al^{i}Bu_3/B(C_6F_5)_3$ systems in Table 3, entry 5.

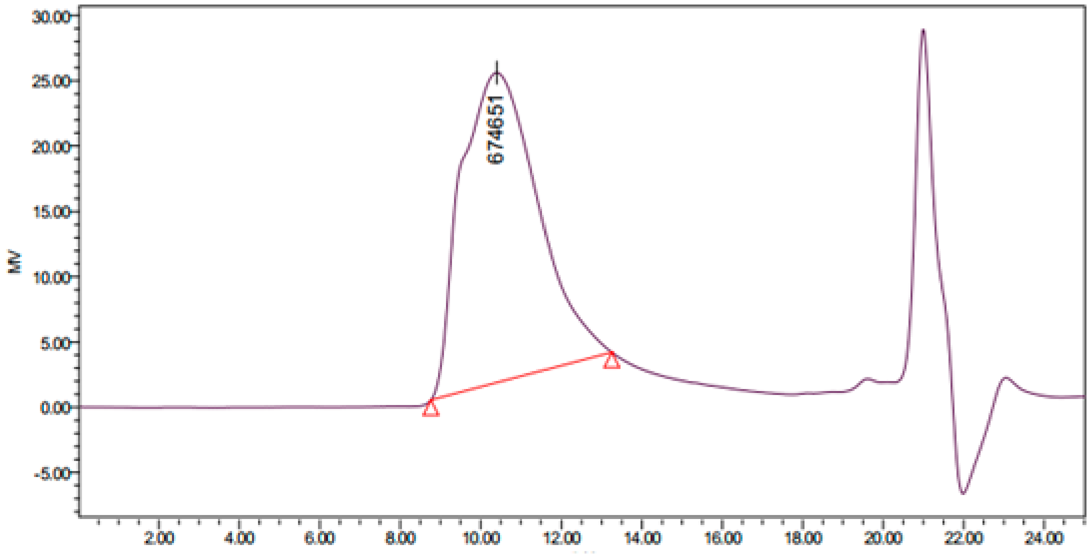

GPC Results

	Dist Name	Elution Volume (ml)	Retention Time (min)	Adjusted RT (min)	Mn	Mw	MP	Mz	Mz+1	Mz/Mw
1		10.404	10.404	10.404	397945	893107	674651	1634858	2320532	1.830529

Figure S47. GPC profiles of the PMYs by the **3**/$Al^{i}Bu_3/B(C_6F_5)_3$ systems in Table 3, entry 6.

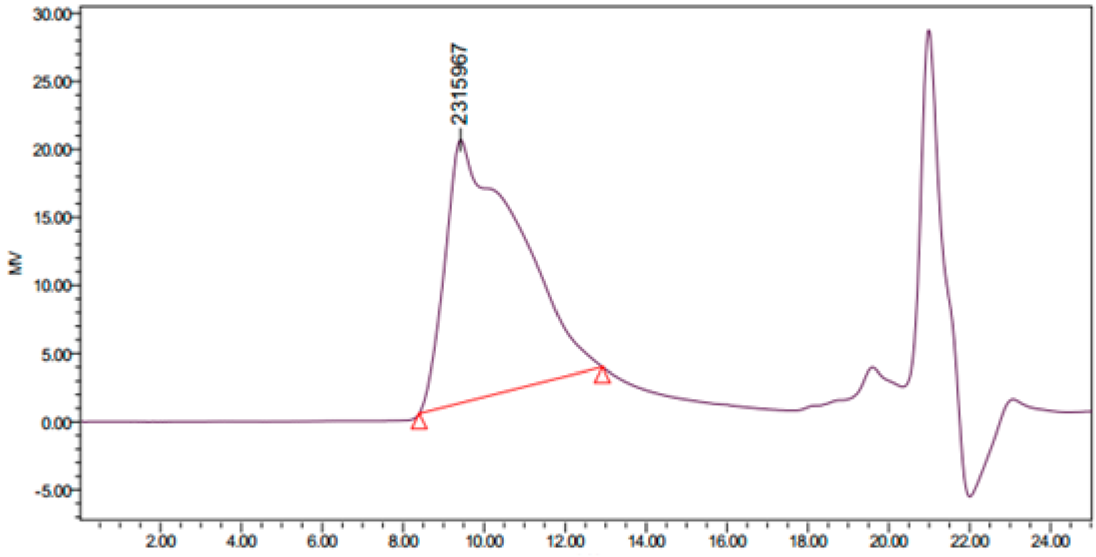

GPC Results

	Dist Name	Elution Volume (ml)	Retention Time (min)	Adjusted RT (min)	Mn	Mw	MP	Mz	Mz+1	Mz/Mw
1		9.425	9.425	9.425	535003	1458493	2315967	2877816	4169333	1.973144

Figure S48. GPC profiles of the PMYs by the **3**/Al^{i}Bu$_3$/B(C_6F_5)$_3$ systems in Table 3, entry 7.

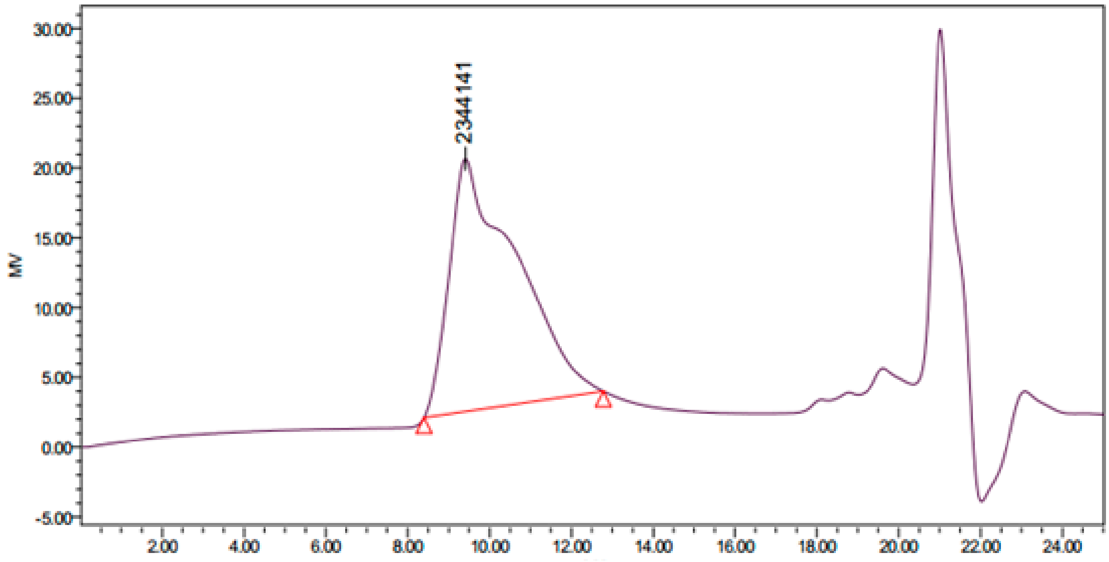

GPC Results

	Dist Name	Elution Volume (ml)	Retention Time (min)	Adjusted RT (min)	Mn	Mw	MP	Mz	Mz+1	Mz/Mw
1		9.416	9.416	9.416	628537	1622660	2344141	3050908	4330229	1.880189

Figure S49. GPC profiles of the PMYs by the **3**/Al^{i}Bu$_3$/B(C_6F_5)$_3$ systems in Table 3, entry 8.

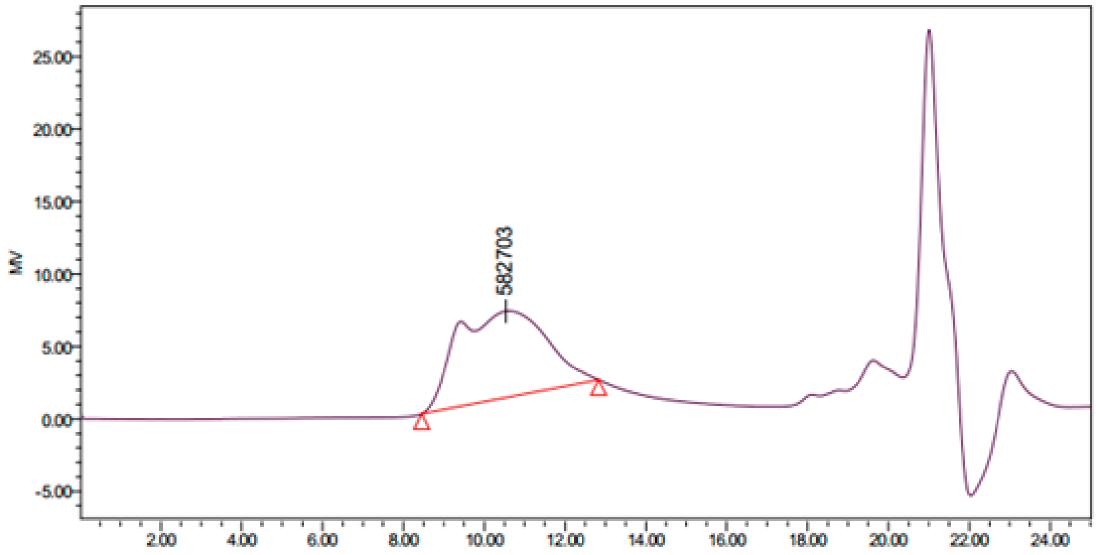

GPC Results

	Dist Name	Elution Volume (ml)	Retention Time (min)	Adjusted RT (min)	Mn	Mw	MP	Mz	Mz+1	Mz/Mw
1		10.533	10.533	10.533	447054	1166305	582703	2457113	3653218	2.106750

Figure S50. GPC profiles of the PMYs by the **3**/$Al^{i}Bu_3$/$B(C_6F_5)_3$ systems in Table 3, entry 9.

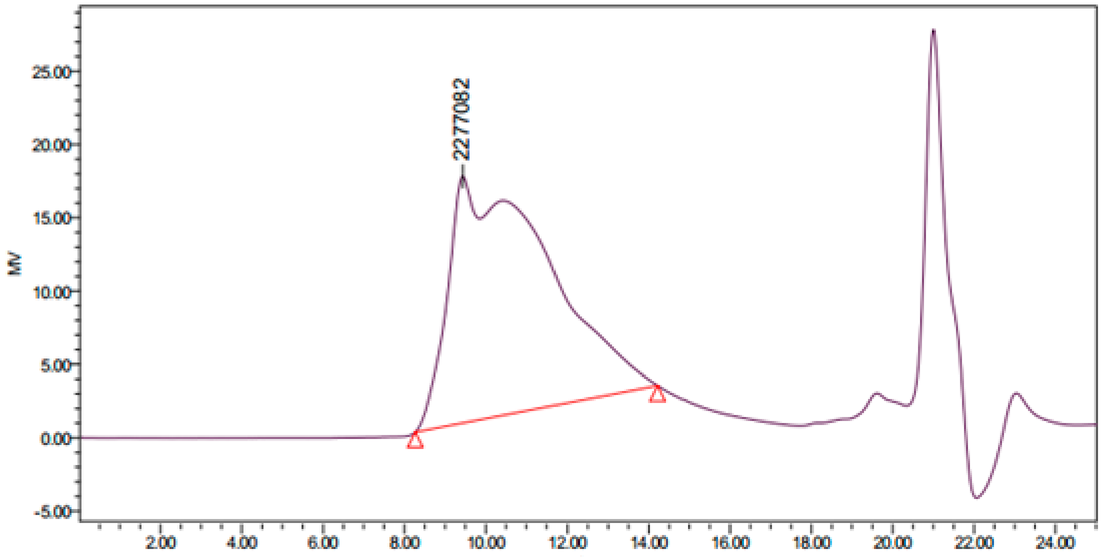

GPC Results

	Dist Name	Elution Volume (ml)	Retention Time (min)	Adjusted RT (min)	Mn	Mw	MP	Mz	Mz+1	Mz/Mw
1		9.437	9.437	9.437	273547	1164120	2277082	2973833	4759669	2.554575

Figure S51. GPC profiles of the PMYs by the **3**/$Al^{i}Bu_3$/$B(C_6F_5)_3$ systems in Table 3, entry 10.

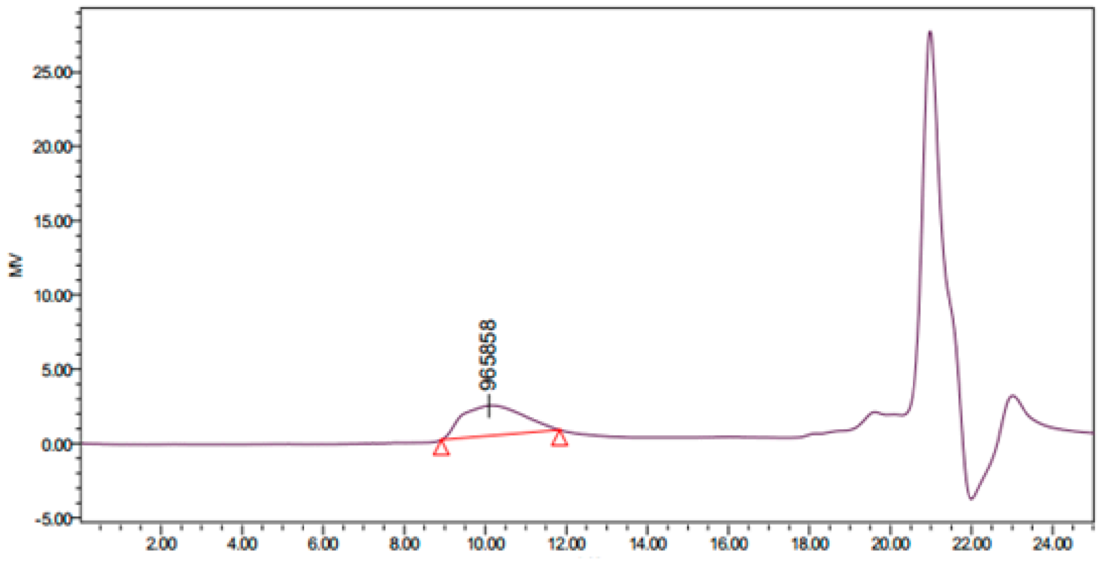

GPC Results

	Dist Name	Elution Volume (ml)	Retention Time (min)	Adjusted RT (min)	Mn	Mw	MP	Mz	Mz+1	Mz/Mw
1		10.100	10.100	10.100	679221	1124459	965858	1699893	2218535	1.511743

Figure S51. GPC profiles of the PMYs by the **3**/Al^{i}Bu$_3$/B(C_6F_5)$_3$ systems in Table 3, entry 11.

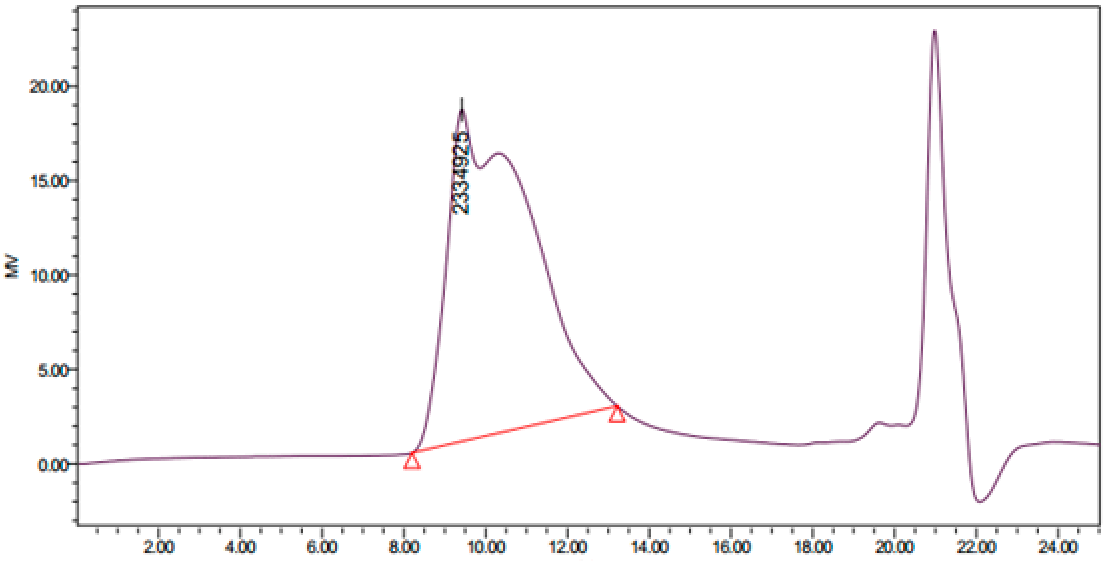

GPC Results

	Dist Name	Elution Volume (ml)	Retention Time (min)	Adjusted RT (min)	Mn	Mw	MP	Mz	Mz+1	Mz/Mw
1		9.419	9.419	9.419	459488	1381953	2334925	3014553	4661482	2.181372

Figure S53. GPC profiles of the PMYs by the **3**/Al^{i}Bu$_3$/B(C_6F_5)$_3$ systems in Table 3, entry 12.

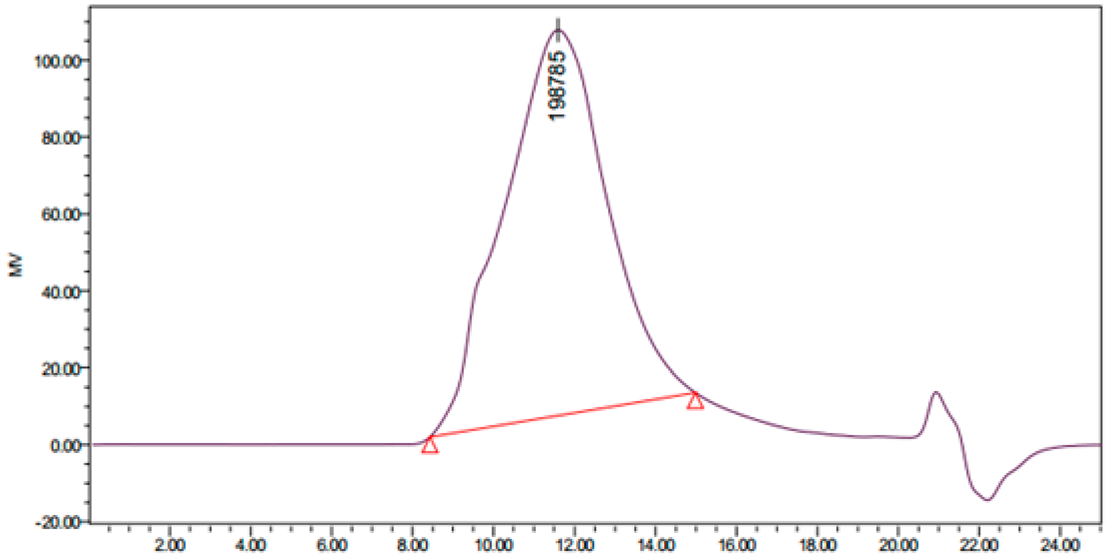

GPC Results

	Dist Name	Elution Volume (ml)	Retention Time (min)	Adjusted RT (min)	Mn	Mw	MP	Mz	Mz+1	Mz/Mw
1		11.595	11.595	11.595	140680	465443	198785	1621659	3363332	3.484122

Figure S54. GPC profiles of the PMYs by the **3**/Al^{i}Bu$_3$/B(C_6F_5)$_3$ systems in Table 3, entry 13.

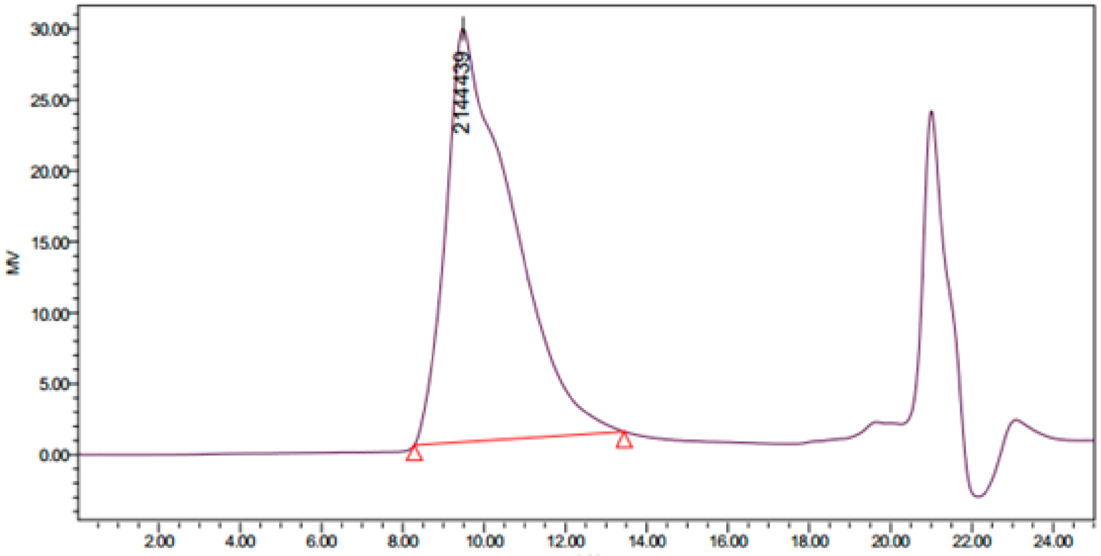

GPC Results

	Dist Name	Elution Volume (ml)	Retention Time (min)	Adjusted RT (min)	Mn	Mw	MP	Mz	Mz+1	Mz/Mw
1		9.481	9.481	9.481	595297	1611784	2144439	3143194	4761487	1.950134

Figure S55. GPC profiles of the PMYs by the **3**/Al^{i}Bu$_3$/B(C_6F_5)$_3$ systems in Table 3, entry 14.

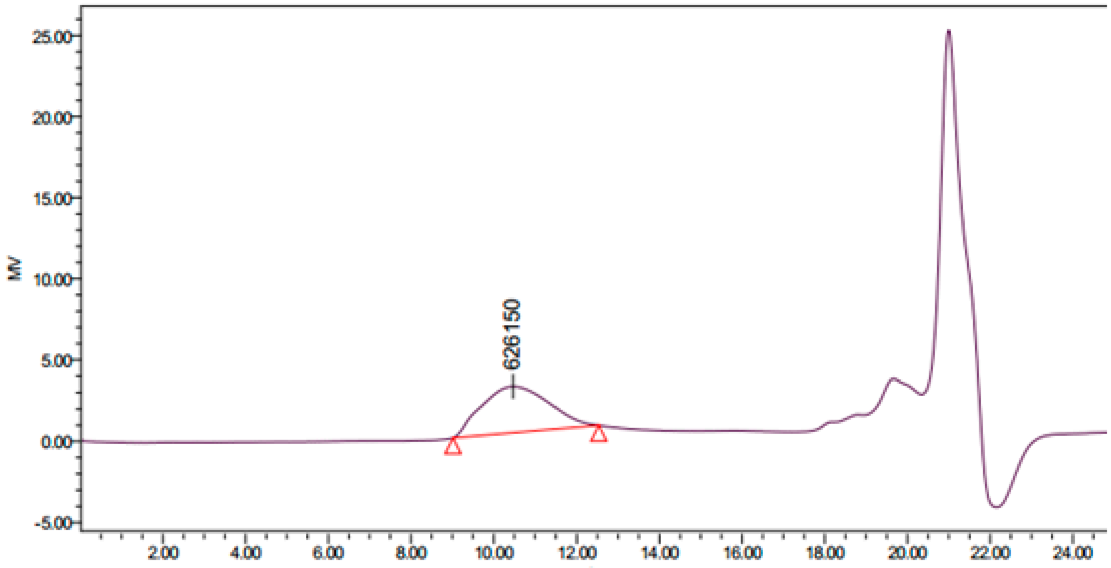

GPC Results

	Dist Name	Elution Volume (ml)	Retention Time (min)	Adjusted RT (min)	Mn	Mw	MP	Mz	Mz+1	Mz/Mw
1		10.469	10.469	10.469	452569	791735	626150	1288577	1783413	1.627536

Figure S57. GPC profiles of the PMYs by the **3**/Al^{i}Bu$_3$/B(C_6F_5)$_3$ systems in Table 3, entry 15.

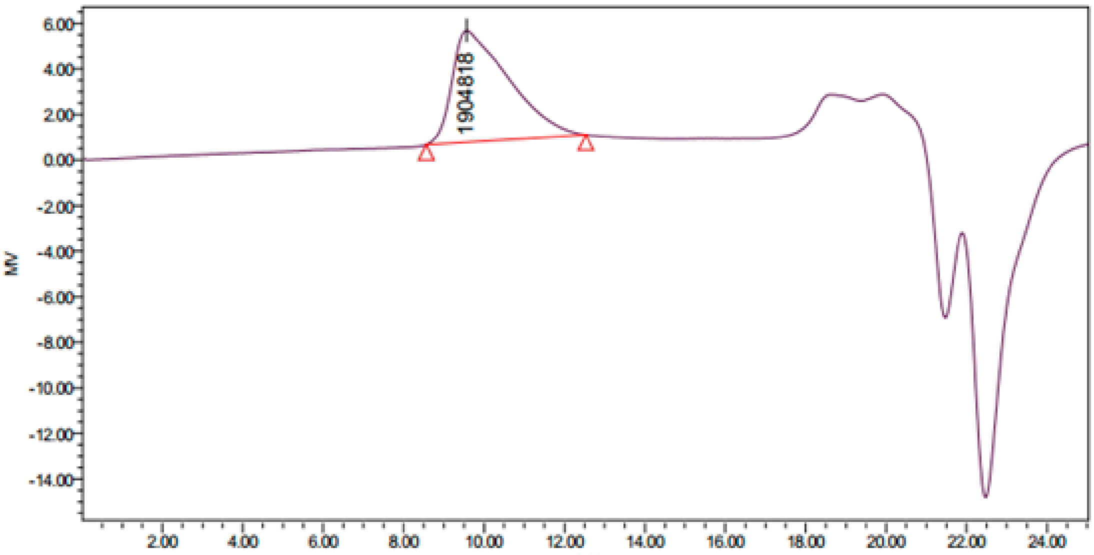

GPC Results

	Dist Name	Elution Volume (ml)	Retention Time (min)	Adjusted RT (min)	Mn	Mw	MP	Mz	Mz+1	Mz/Mw
1		9.569	9.569	9.569	701953	1375515	1904818	2184051	2943875	1.587806

Figure S57. GPC profiles of the PMYs by the **3**/Al^{i}Bu$_3$/B(C_6F_5)$_3$ systems in Table 3, entry 16.

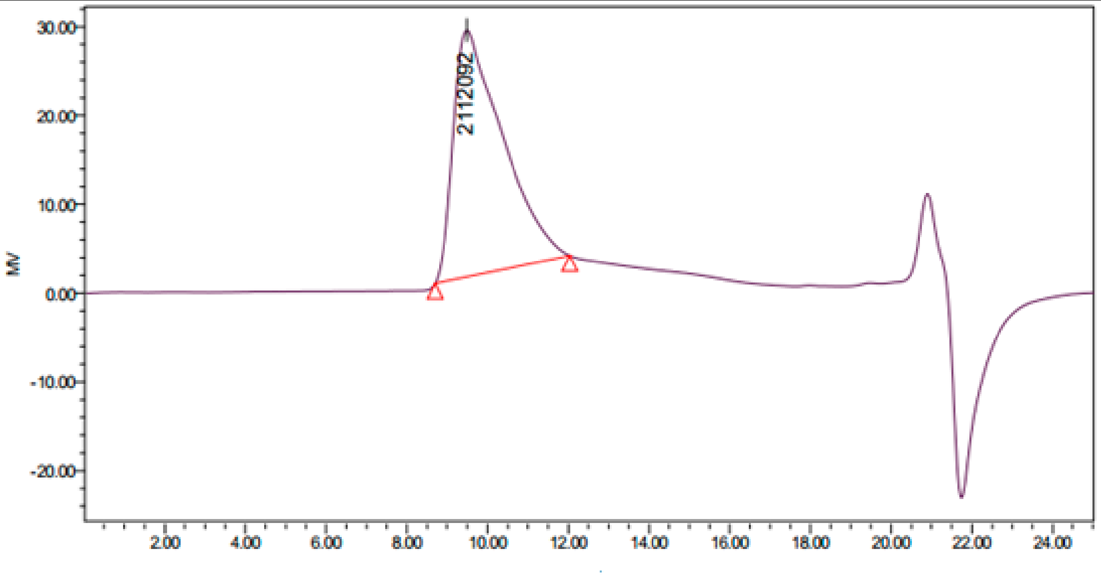

GPC Results

	Dist Name	Elution Volume (ml)	Retention Time (min)	Adjusted RT (min)	Mn	Mw	MP	Mz	Mz+1	Mz/Mw
1		9.492	9.492	9.492	895345	1575353	2112092	2298577	2899308	1.459087

Figure S58. GPC profiles of the PMYs by the **3**/Al^{i}Bu$_3$/B(C_6F_5)$_3$ systems in Table 3, entry 17.

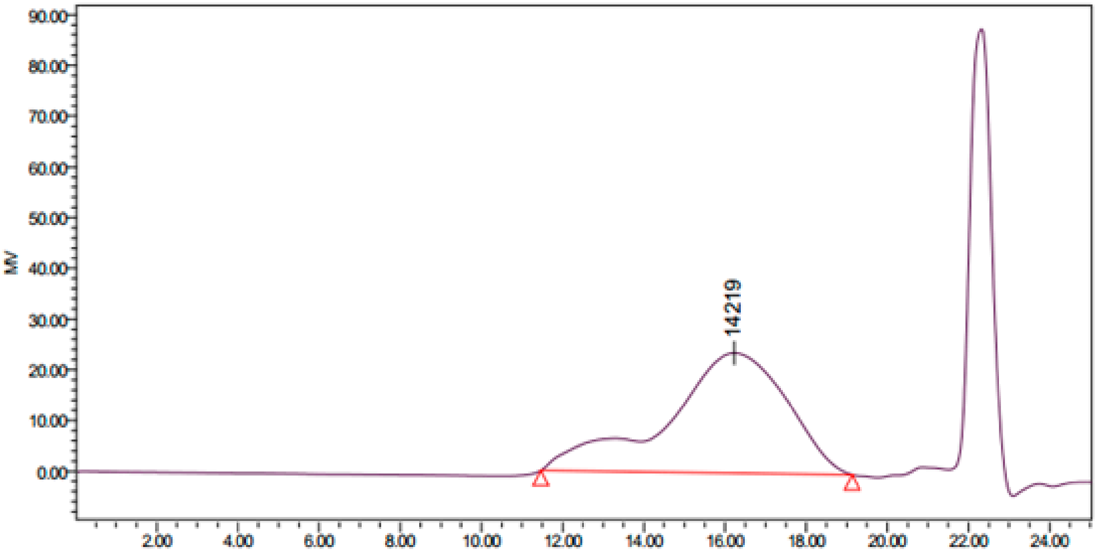

GPC Results

	Dist Name	Elution Volume (ml)	Retention Time (min)	Adjusted RT (min)	Mn	Mw	MP	Mz	Mz+1	Mz/Mw	Mz+1/Mw
1		16.229	16.229	16.229	12950	90761	14219	903780	1706874	9.957751	18.806161

Figure S59. GPC profiles of the PMYs by the Y(CH_2SiCH_3)$_3$(THF)$_2$/Al^{i}Bu$_3$/B(C_6F_5)$_3$ systems in Table 3, entry 18.

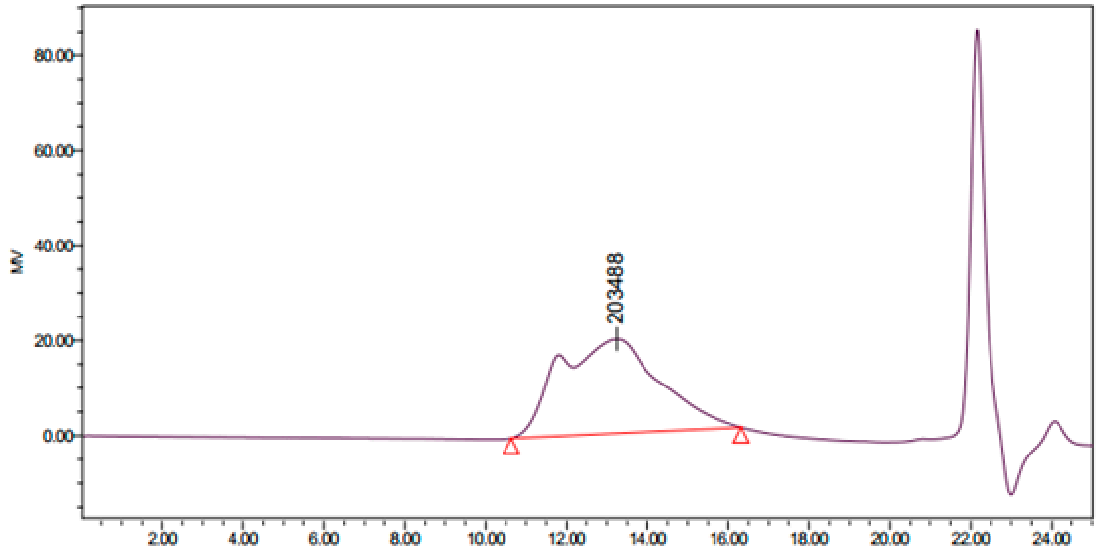

GPC Results

	Dist Name	Elution Volume (ml)	Retention Time (min)	Adjusted RT (min)	Mn	Mw	MP	Mz	Mz+1	Mz/Mw
1		13.249	13.249	13.249	125364	901479	203488	4017285	8017647	4.456326

Figure S60. GPC profiles of the PMYs by the $Y(CH_2SiCH_3)_3(THF)_2/Al^iBu_3/B(C_6F_5)_3$ systems in Table 3, entry 19.

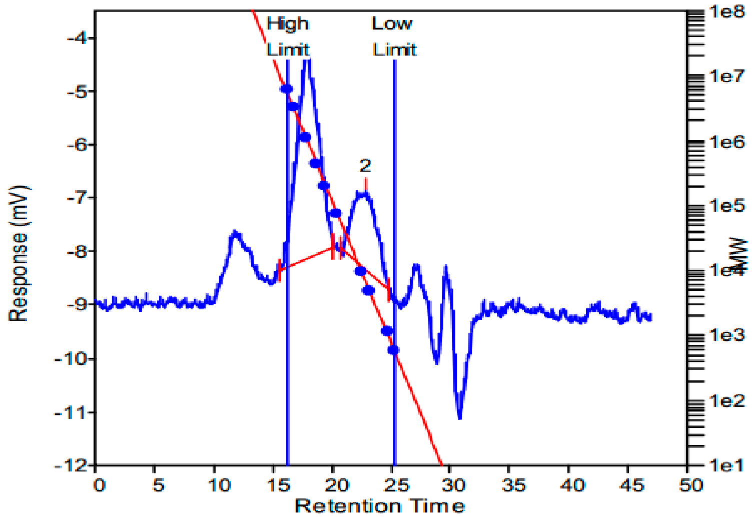

Peak No	Mp	Mn	Mw	Mz	Mz+1	Mv	PD
1	1046218	711423	1457620	2735502	4236678	1298313	2.04888
2	7336	5378	10279	17203	23431	9291	1.91131

Figure S61. GPC profiles of the PSTs by the **1**/Al^iBu_3/$[PhNHMe_2][B(C_6F_5)_4]$ systems in Table 4, entry 1.

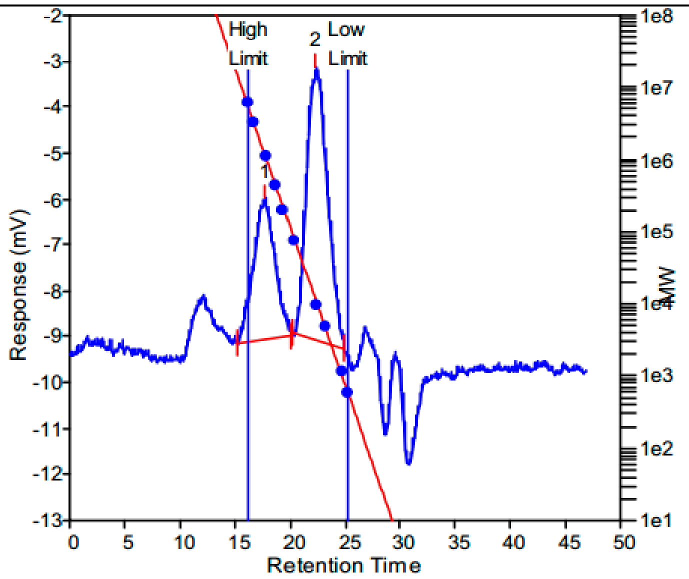

Peak No	Mp	Mn	Mw	Mz	Mz+1	Mv	PD
1	1176894	883316	2105060	4270577	6677476	1839908	2.38313
2	11946	7227	14879	25143	35179	13422	2.05881

Figure S62. GPC profiles of the PSTs by the **1**/Al^{i}Bu$_3$/[PhNHMe$_2$][B(C$_6$F$_5$)$_4$] systems in Table 4, entry 2.

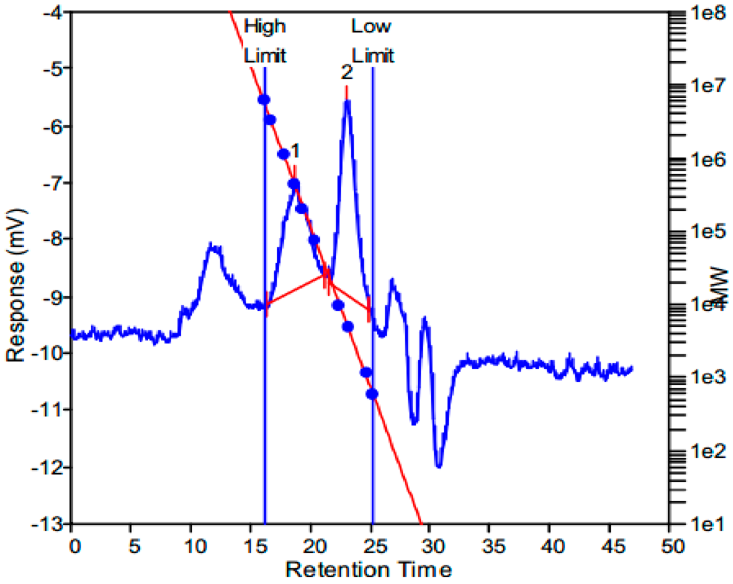

Peak No	Mp	Mn	Mw	Mz	Mz+1	Mv	PD
1	421994	288994	680987	1306923	1930394	598543	2.35641
2	5797	4378	6463	8801	11050	6103	1.47624

Figure S63. GPC profiles of the PSTs by the **1**/Al^{i}Bu$_3$/[PhNHMe$_2$][B(C$_6$F$_5$)$_4$] systems in Table 4, entry 3.

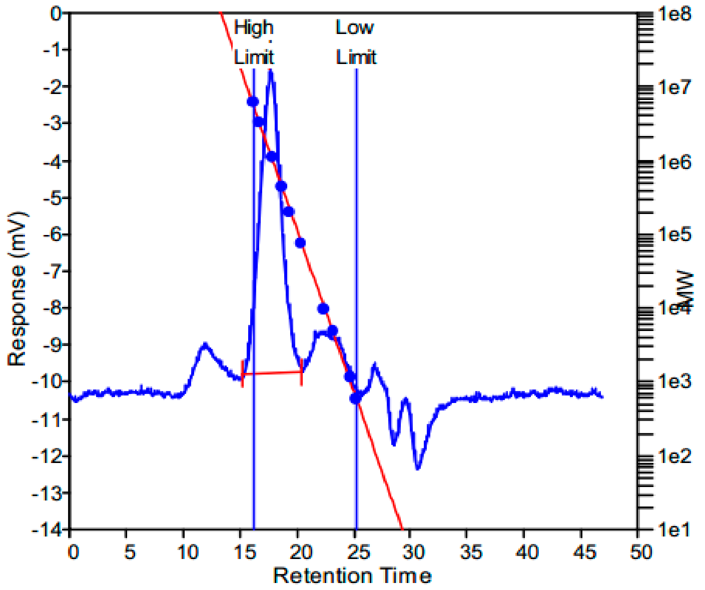

Peak No	Mp	Mn	Mw	Mz	Mz+1	Mv	PD
1	1392383	878005	1941932	3669536	5622787	1722642	2.21176

Figure S64. GPC profiles of the PSTs by the **1**/Al^{i}Bu$_3$/[PhNHMe$_2$][B(C_6F_5)$_4$] systems in Table 4, entry 5.

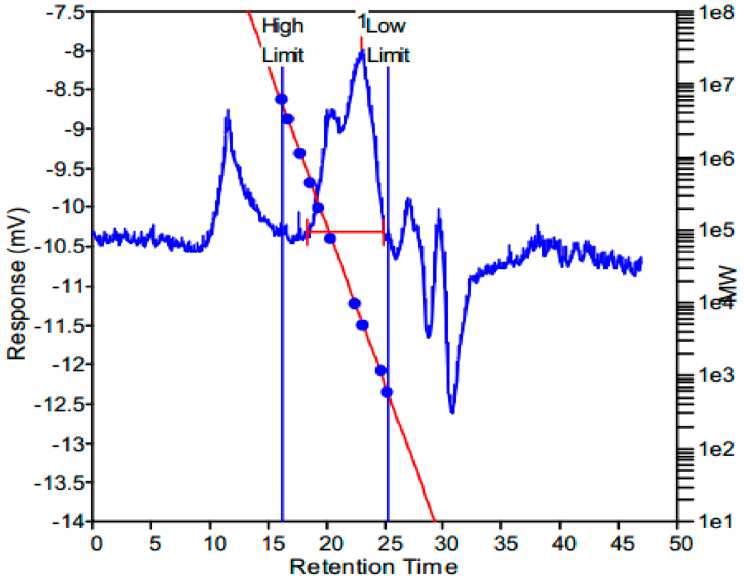

Peak No	Mp	Mn	Mw	Mz	Mz+1	Mv	PD
1	5330	7271	46678	156113	246425	34820	6.41975

Figure S65. GPC profiles of the PSTs by the **1**/Al^{i}Bu$_3$/[PhNHMe$_2$][B(C_6F_5)$_4$] systems in Table 4, entry 6.

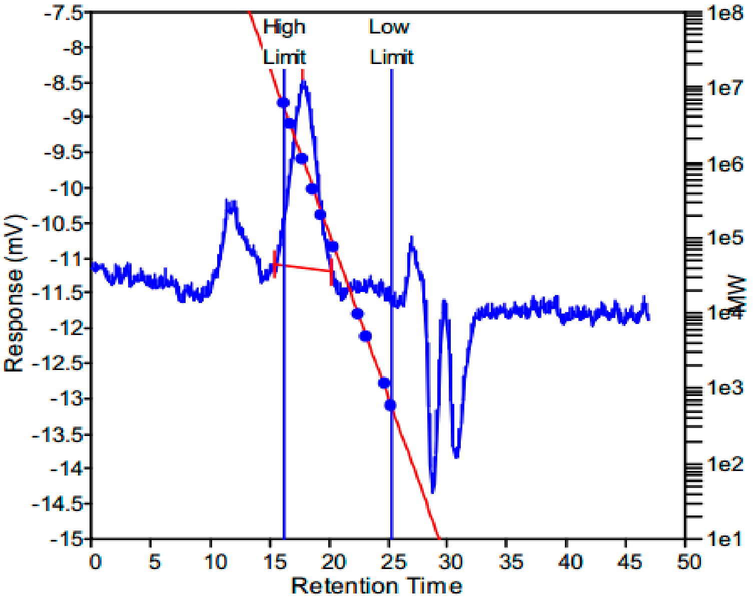

Peak No	Mp	Mn	Mw	Mz	Mz+1	Mv	PD
1	1157271	731879	1663886	3409254	5357720	1454263	2.27344

Figure S66. GPC profiles of the PSTs by the **1**/Al^{i}Bu$_3$/[PhNHMe$_2$][B(C$_6$F$_5$)$_4$] systems in Table 4, entry 9.

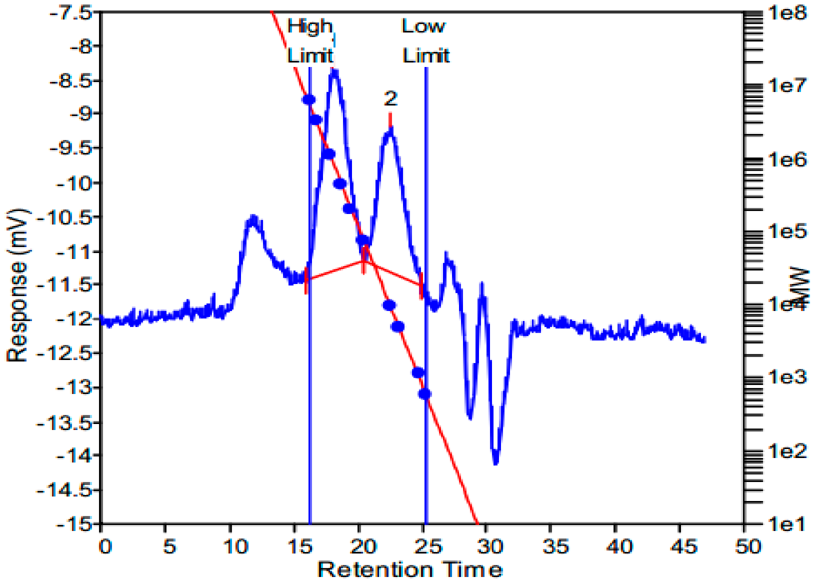

Peak No	Mp	Mn	Mw	Mz	Mz+1	Mv	PD
1	899296	551134	1149336	2121601	3170509	1023508	2.0854
2	9128	6303	12750	21809	30900	11491	2.02285

Figure S67. GPC profiles of the PSTs by the **1**/Al^{i}Bu$_3$/[PhNHMe$_2$][B(C$_6$F$_5$)$_4$] systems in Table 4, entry 12.

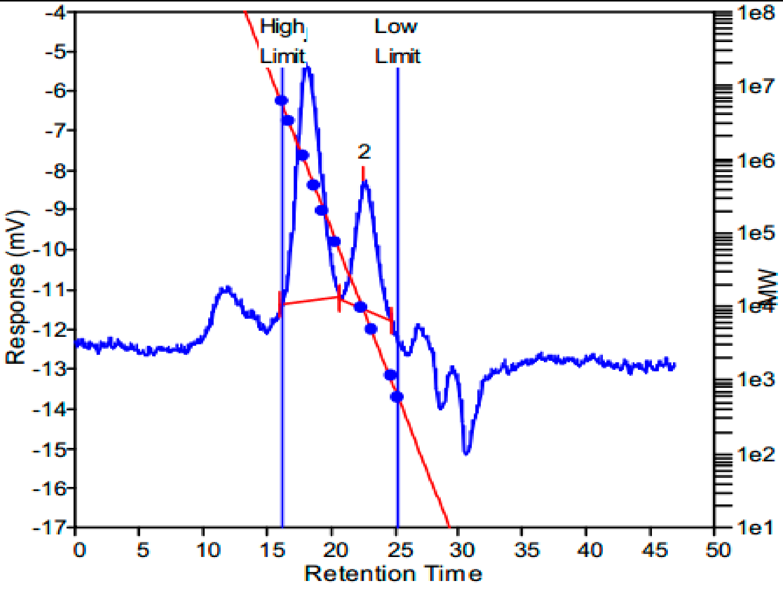

Peak No	Mp	Mn	Mw	Mz	Mz+1	Mv	PD
1	994754	505307	1009791	1714420	2437750	912014	1.99837
2	9283	6273	10676	16850	23623	9824	1.7019

Figure S68. GPC profiles of the PSTs by the **1**/Al^{i}Bu$_3$/[PhNHMe$_2$][B(C$_6$F$_5$)$_4$] systems in Table 4, entry 13.

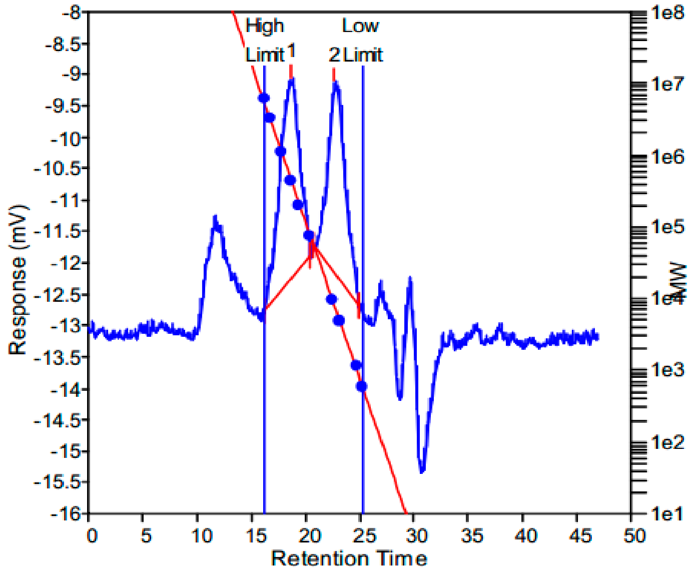

Peak No	Mp	Mn	Mw	Mz	Mz+1	Mv	PD
1	451351	413514	819683	1537210	2361879	731127	1.98224
2	7846	4964	8791	14464	21067	8036	1.77095

Figure S69. GPC profiles of the PSTs by the **1**/Al^{i}Bu$_3$/[PhNHMe$_2$][B(C$_6$F$_5$)$_4$] systems in Table 4, entry 14.

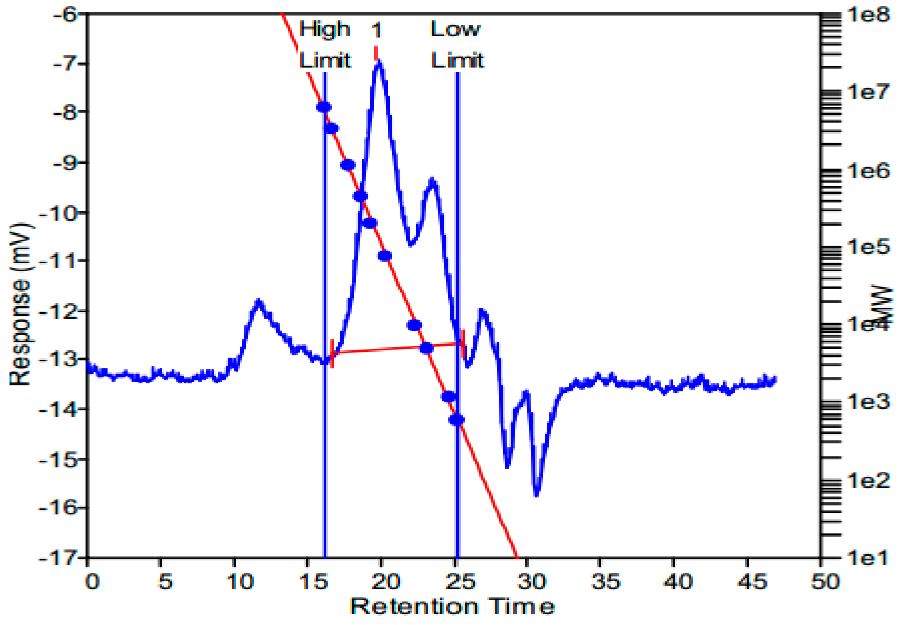

Peak No	Mp	Mn	Mw	Mz	Mz+1	Mv	PD
1	151312	8846	166108	600191	1082524	121148	18.7778

Figure S70. GPC profiles of the PSTs by the **1**/Al^{i}Bu$_3$/[PhNHMe$_2$][B(C$_6$F$_5$)$_4$] systems in Table 4, entry 15.

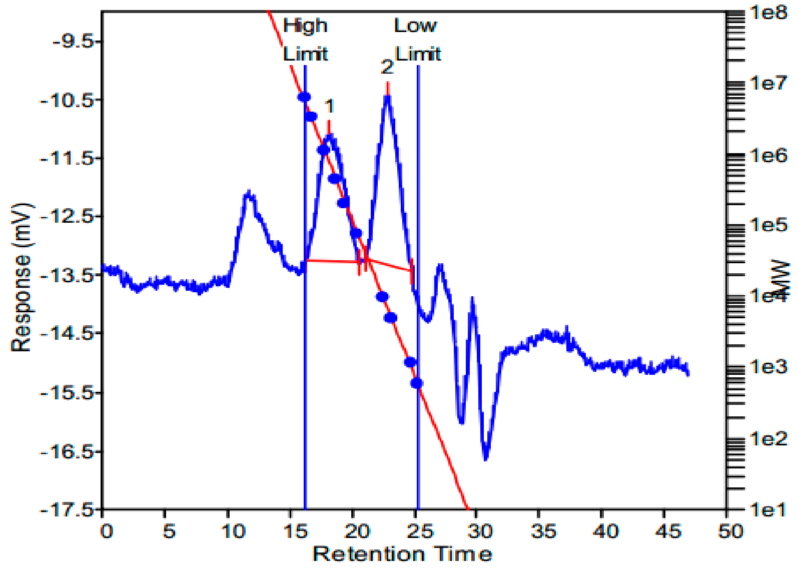

Peak No	Mp	Mn	Mw	Mz	Mz+1	Mv	PD
1	722728	452846	914476	1579249	2236213	822428	2.0194
2	7093	5267	8719	13350	17949	8055	1.6554

Figure S71. GPC profiles of the PSTs by the **1**/Al^{i}Bu$_3$/[PhNHMe$_2$][B(C$_6$F$_5$)$_4$] systems in Table 4, entry 16.

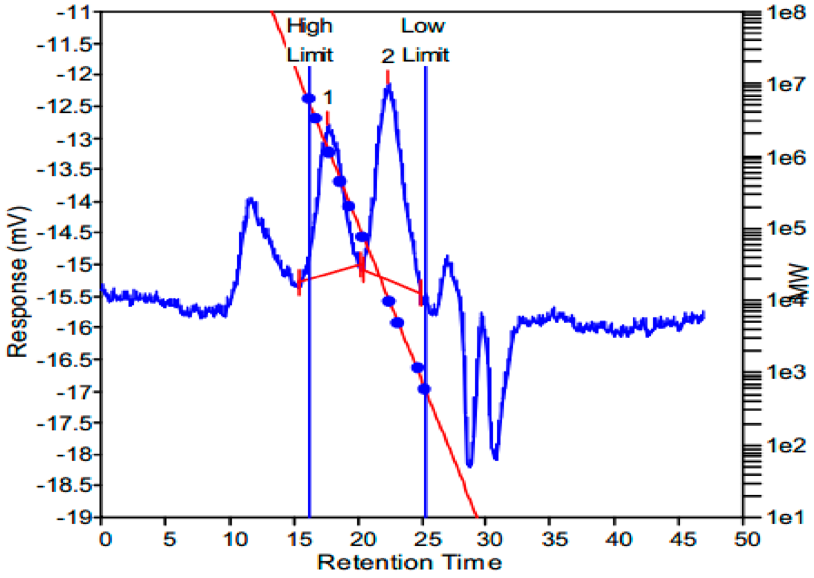

Peak No	Mp	Mn	Mw	Mz	Mz+1	Mv	PD
1	1196849	763874	1550112	2899559	4587761	1383692	2.02928
2	11169	6500	13875	24540	35177	12409	2.13462

Figure S72. GPC profiles of the PSTs by the **1**/Al^{i}Bu$_3$/[PhNHMe$_2$][B(C$_6$F$_5$)$_4$] systems in Table 4, entry 17.

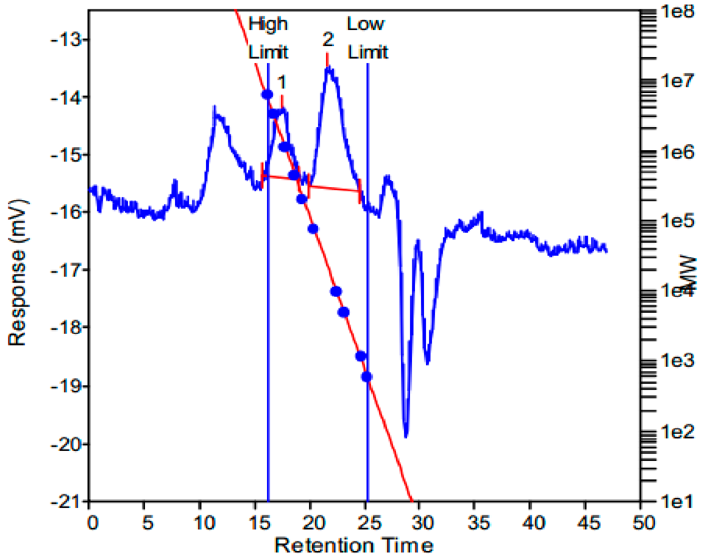

Peak No	Mp	Mn	Mw	Mz	Mz+1	Mv	PD
1	1540181	1303793	1937667	2772342	3659743	1817791	1.48618
2	21519	10056	21535	37499	53643	19316	2.14151

Figure S73. GPC profiles of the PSTs by the **1**/Al^{i}Bu$_3$/[PhNHMe$_2$][B(C$_6$F$_5$)$_4$] systems in Table 4, entry 18.

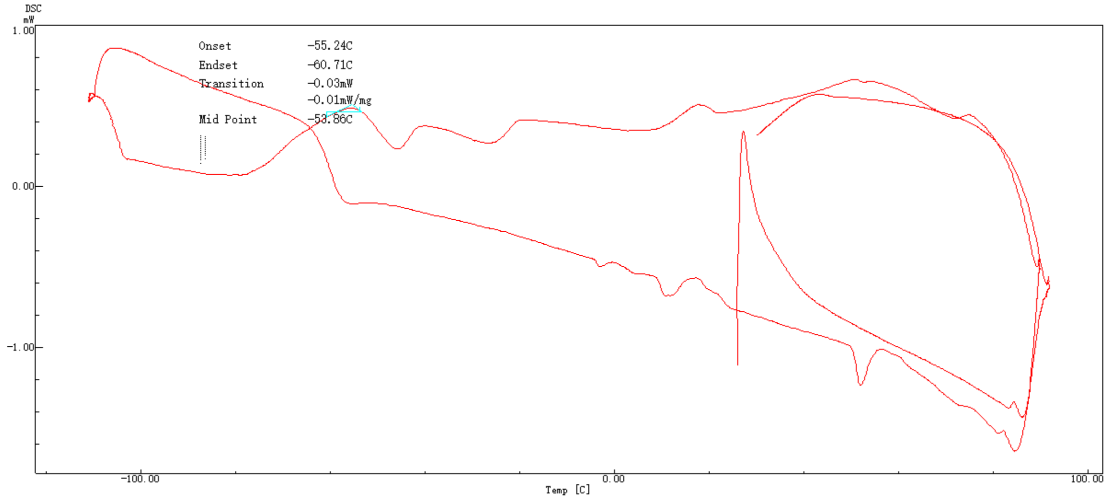

Figure S74. DSC charts of the PIPs by the **3**/Al^{i}Bu$_3$/[Ph$_3$C][B(C$_6$F$_5$)$_4$] systems in Table 2, entry 1.

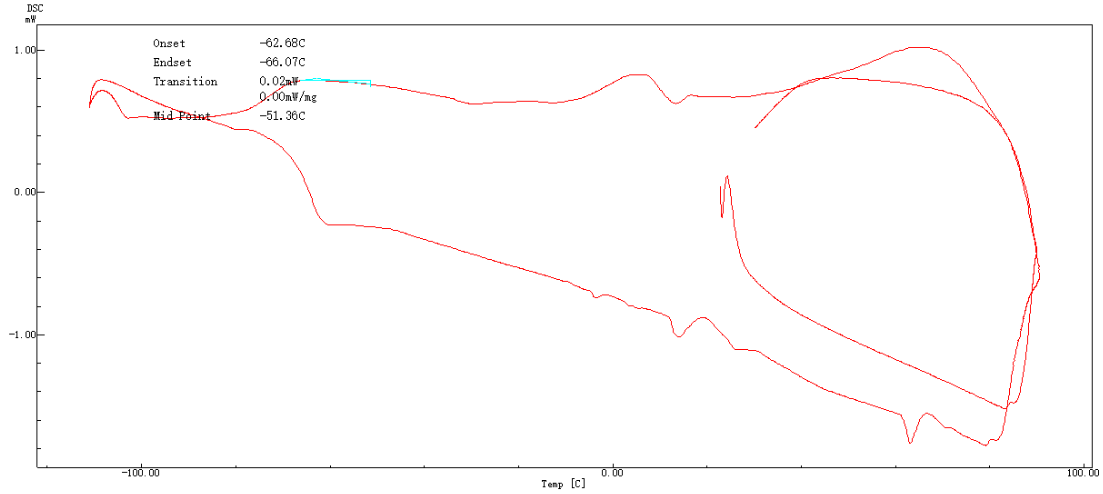

Figure S75. DSC charts of the PIPs by the **3**/Al^{i}Bu$_3$/[PhNHMe$_2$][B(C$_6$F$_5$)$_4$] systems in Table 2, entry 2.

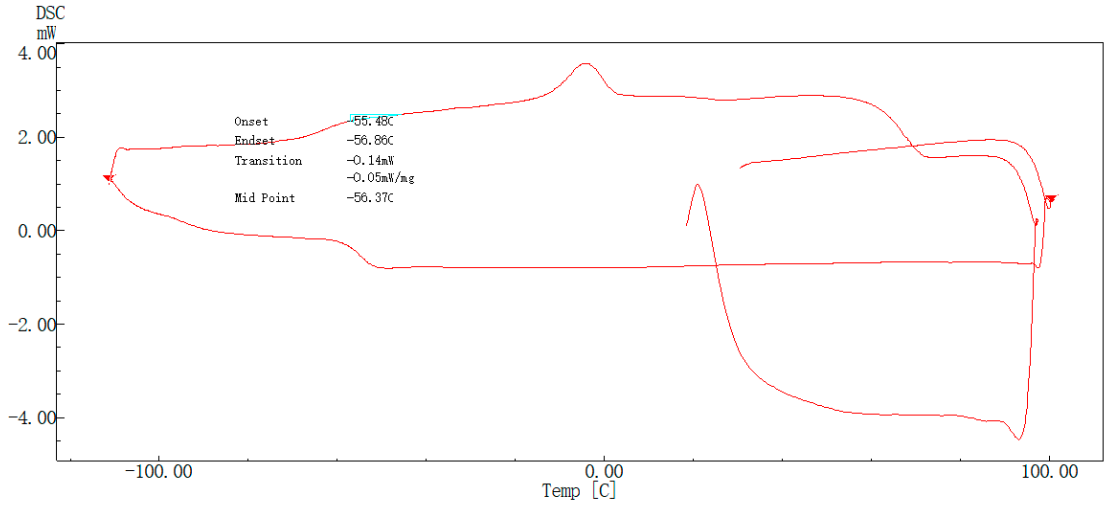

Figure S76. DSC charts of the PIPs by the **1**/Al^{i}Bu$_3$/[Ph$_3$C][B(C$_6$F$_5$)$_4$] systems in Table 2, entry 4

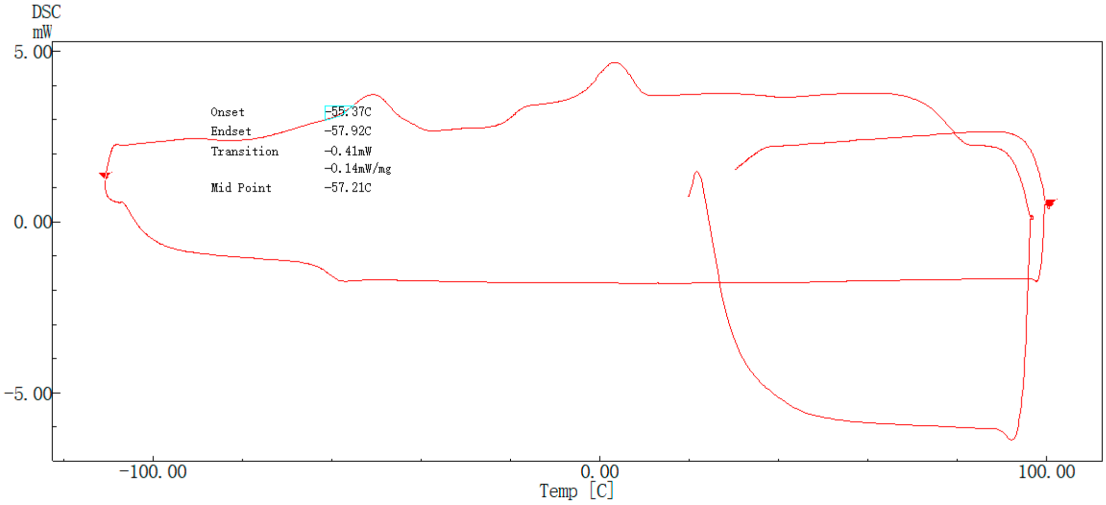

Figure S77. DSC charts of the PIPs by the **2**/Al^{i}Bu$_3$/[Ph$_3$C][B(C$_6$F$_5$)$_4$] systems in Table 2, entry 5.

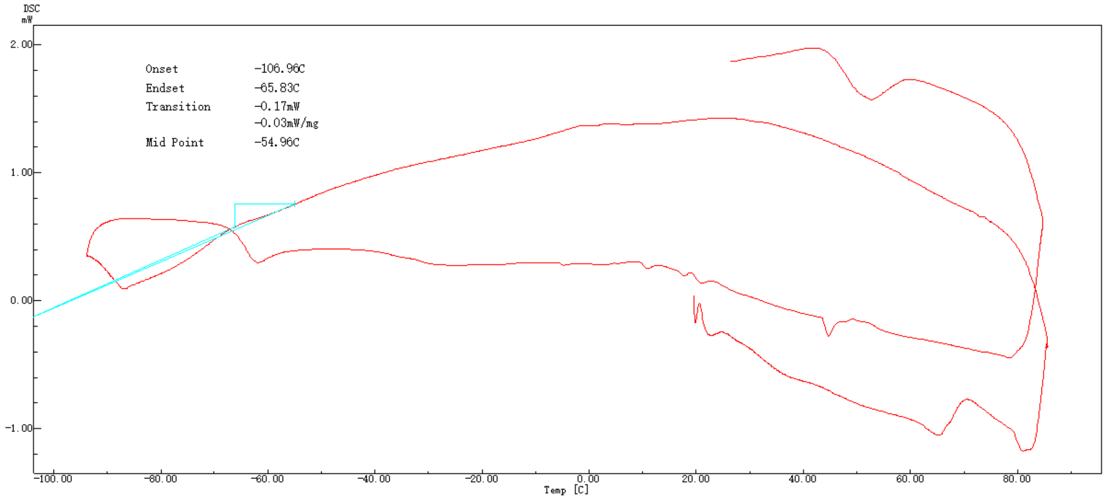

Figure S78. DSC charts of the PIPs by the **3**/Al^{i}Bu$_3$/[Ph$_3$C][B(C$_6$F$_5$)$_4$] systems in Table 2, entry 6.

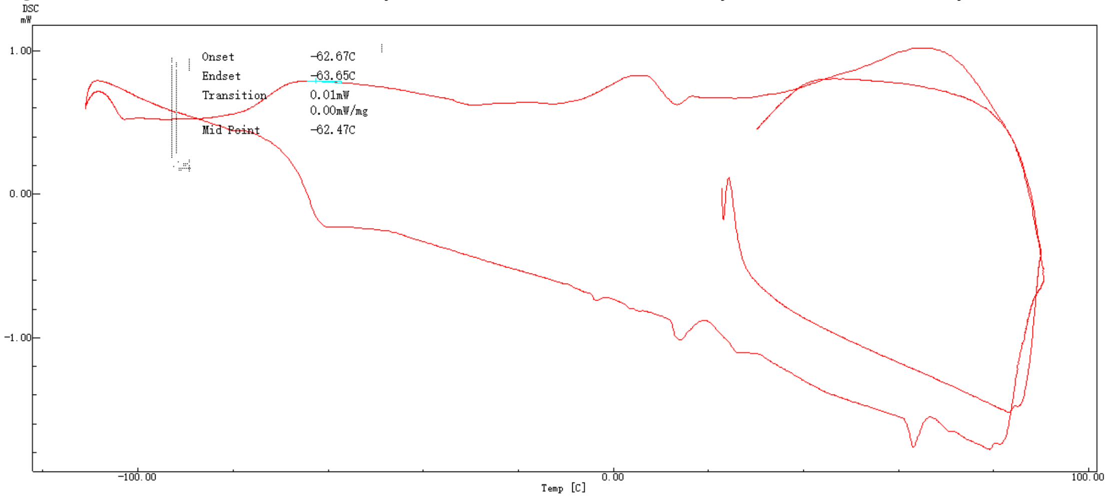

Figure S79. DSC charts of the PIPs by the **1**/AlMe$_3$/[Ph$_3$C][B(C$_6$F$_5$)$_4$] systems in Table 2, entry 7.

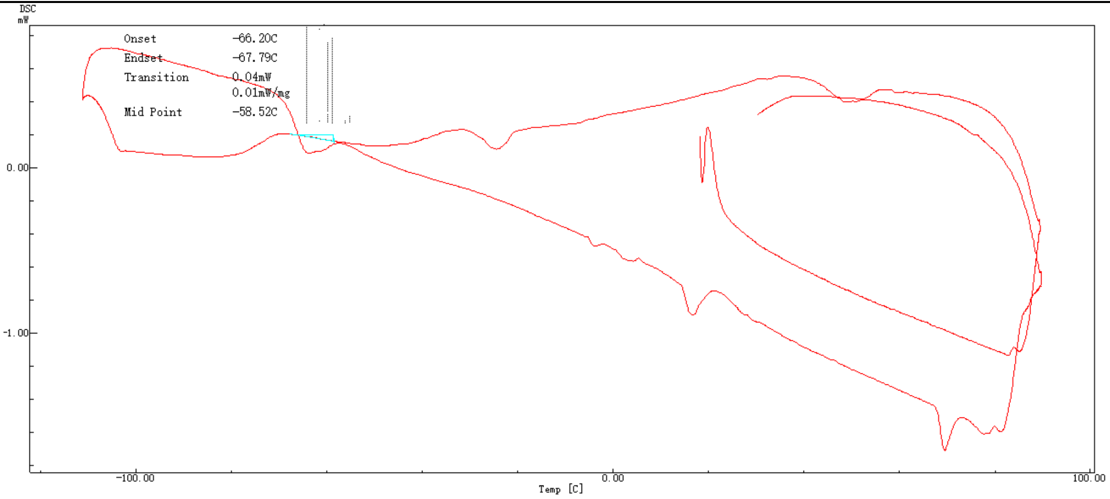

Figure S80. DSC charts of the PIPs by the **1**/$AlEt_3$/$[Ph_3C][B(C_6F_5)_4]$ systems in Table 2, entry 8.

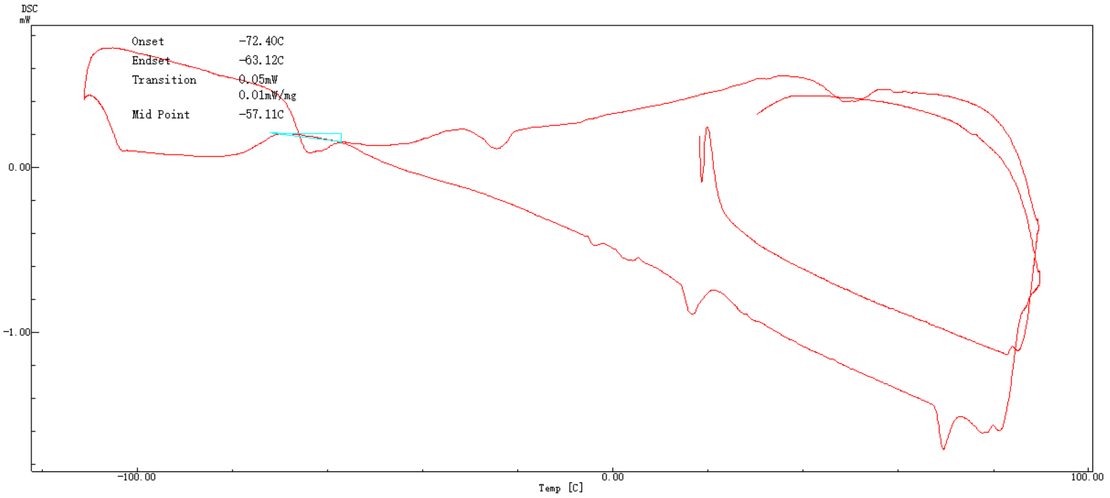

Figure S81. DSC charts of the PIPs by the **1**/$AlMe_3$/$[Ph_3C][B(C_6F_5)_4]$ systems in Table 2, entry 9.

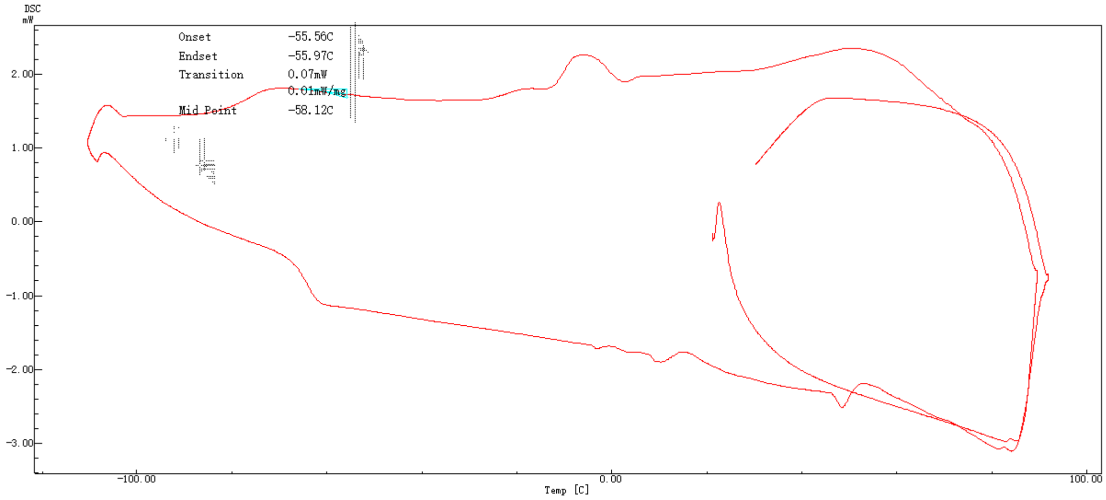

Figure S82. DSC charts of the PIPs by the **1**/$AlMe_3$/$[Ph_3C][B(C_6F_5)_4]$ systems in Table 2, entry 10.

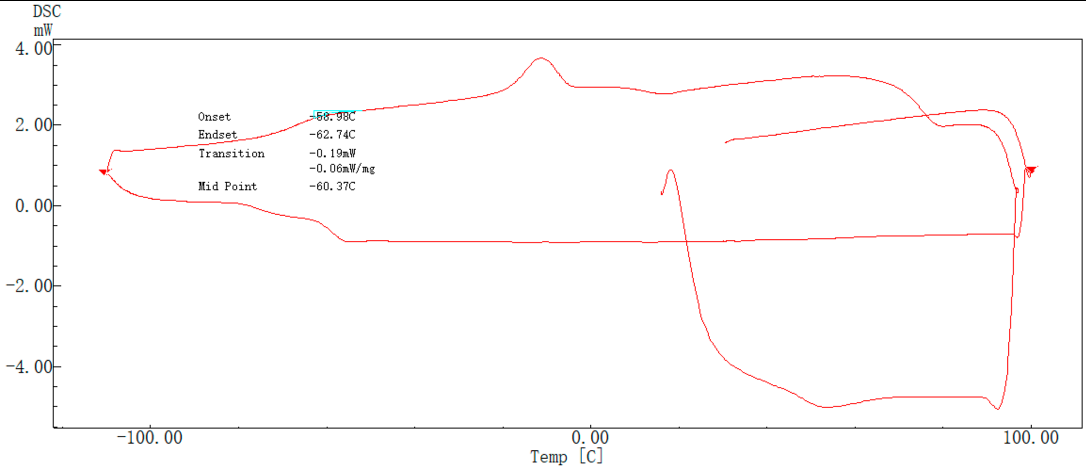

Figure S83. DSC charts of the PIPs by the **1**/$AlMe_3$/[Ph_3C][$B(C_6F_5)_4$] systems in Table 2, entry 11.

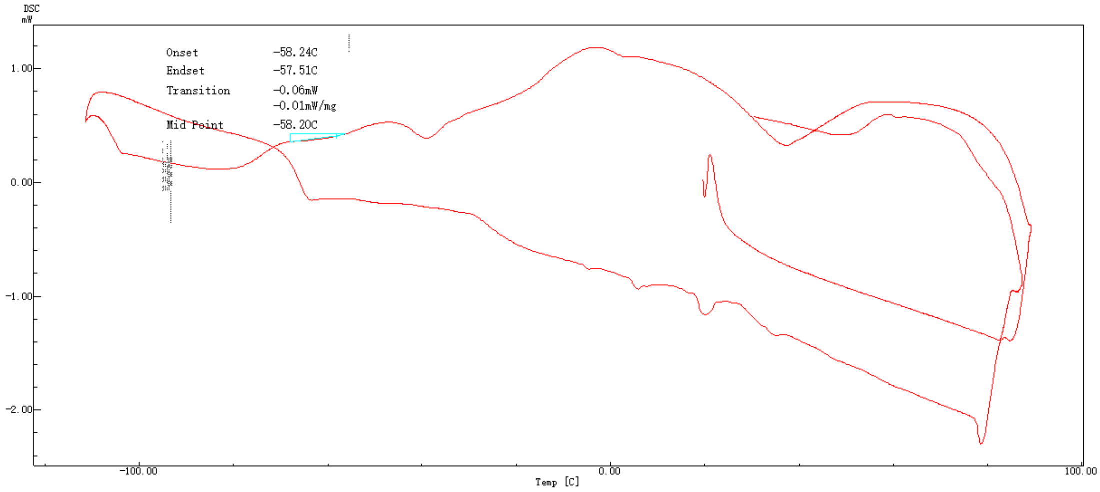

Figure S84. DSC charts of the PIPs by the **1**/$AlMe_3$/[Ph_3C][$B(C_6F_5)_4$] systems in Table 2, entry 12.

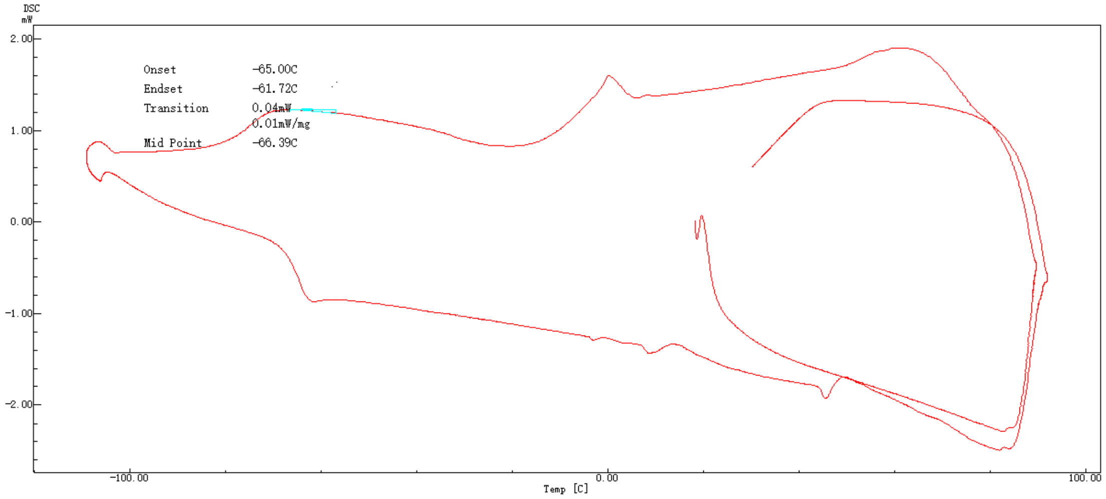

Figure S85. DSC charts of the PIPs by the **1**/$AlMe_3$/[Ph_3C][$B(C_6F_5)_4$] systems in Table 2, entry 13.

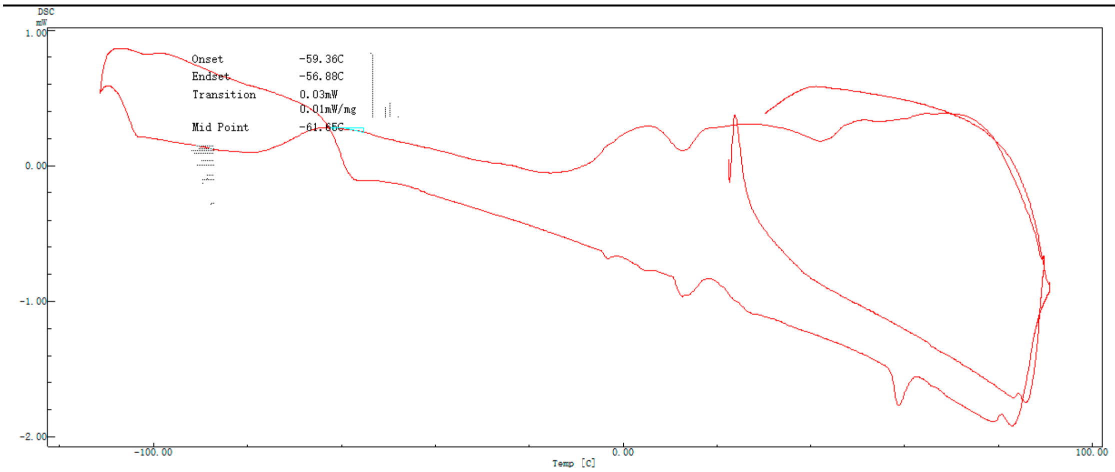

Figure S86. DSC charts of the PIPs by the **1**/$AlMe_3$/$[Ph_3C][B(C_6F_5)_4]$ systems in Table 2, entry 14.

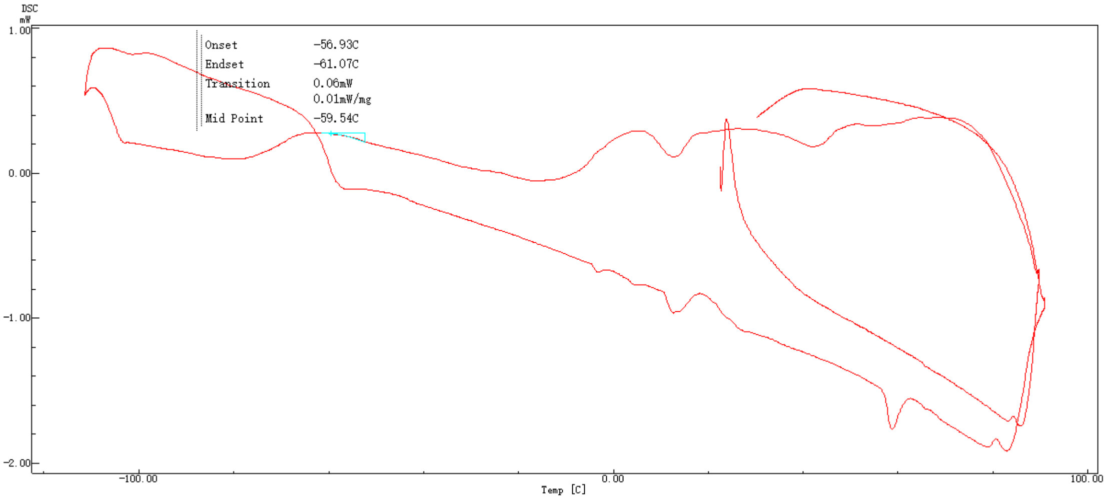

Figure S87. DSC charts of the PIPs by the **1**/$AlMe_3$/$[Ph_3C][B(C_6F_5)_4]$ systems in Table 2, entry 15.

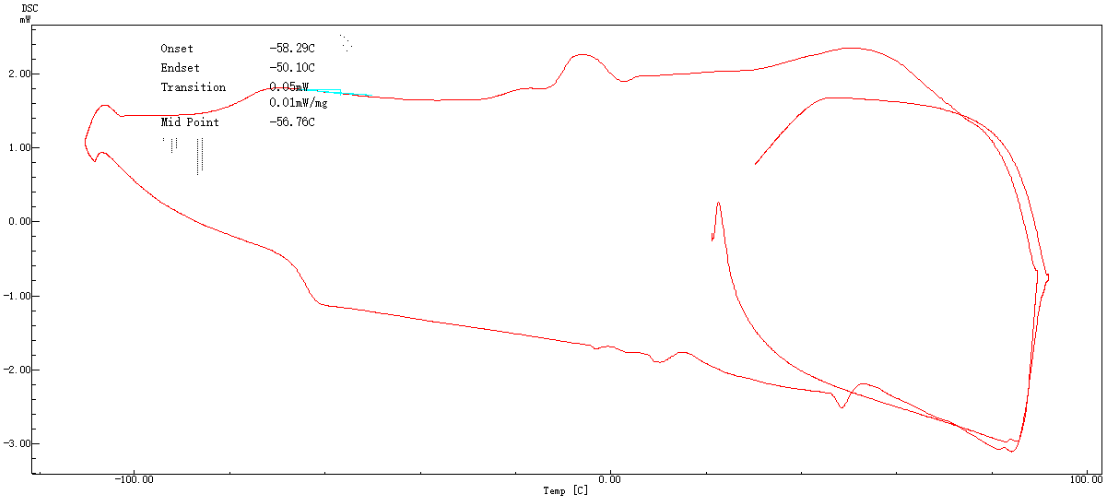

Figure S88. DSC charts of the PIPs by the **1**/Al^iBu_3/$[Ph_3C][B(C_6F_5)_4]$ systems in Table 2, entry 16.

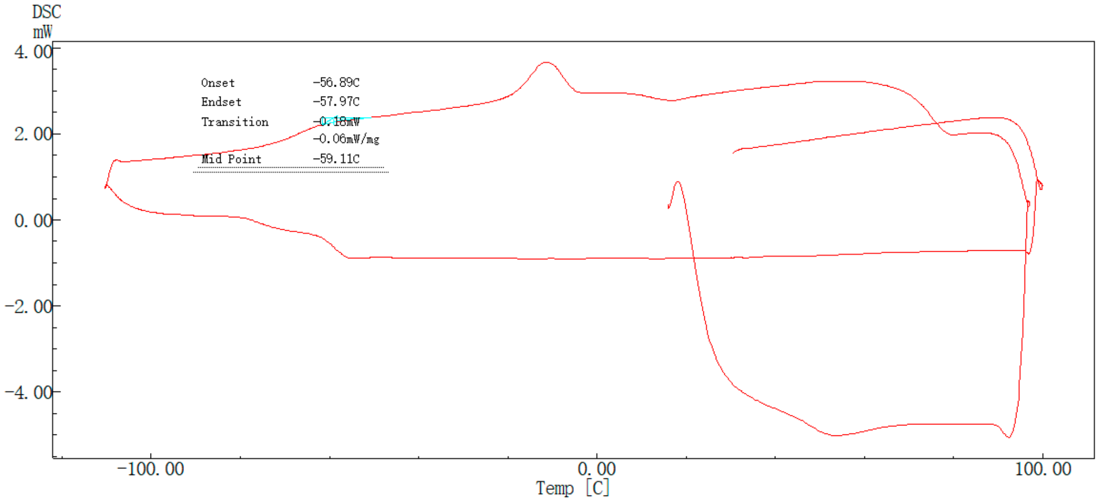

Figure S89. DSC charts of the PIPs by the **1**/$AlMe_3$/[Ph_3C][$B(C_6F_5)_4$] systems in Table 2, entry 17.

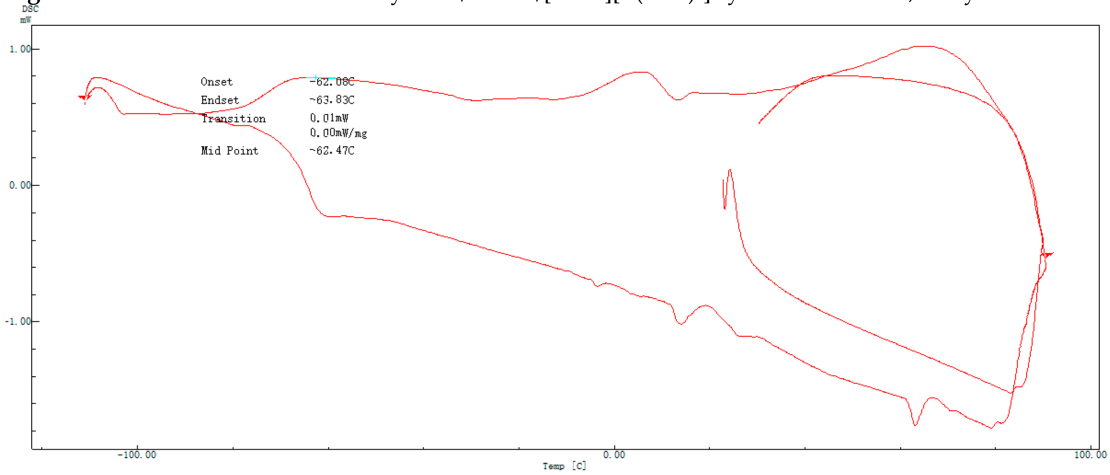

Figure S90. DSC charts of the PIPs by the **1**/$AlMe_3$/[Ph_3C][$B(C_6F_5)_4$] systems in Table 2, entry 18

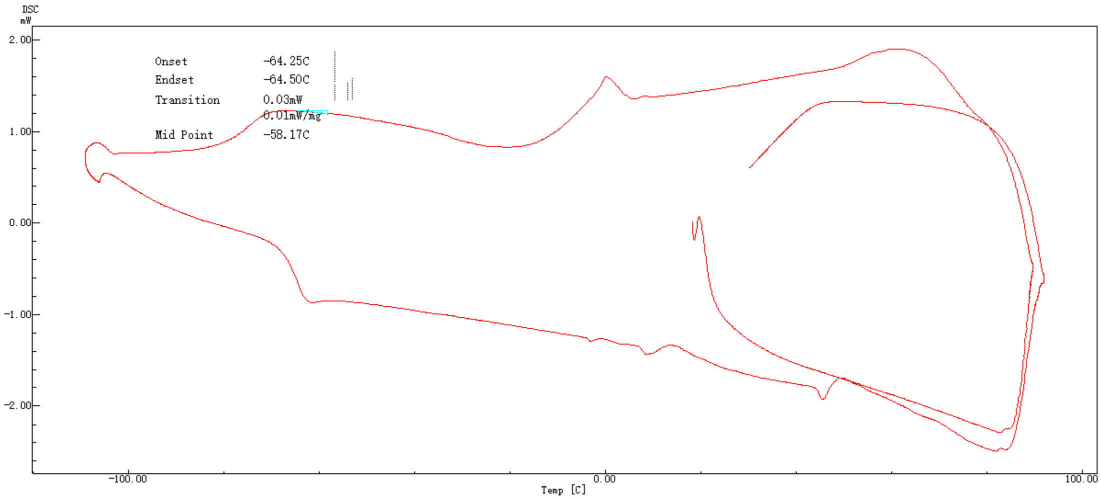

Figure S91. DSC charts of the PIPs by the **1**/$AlMe_3$/[Ph_3C][$B(C_6F_5)_4$]systems in Table 2, entry 19.

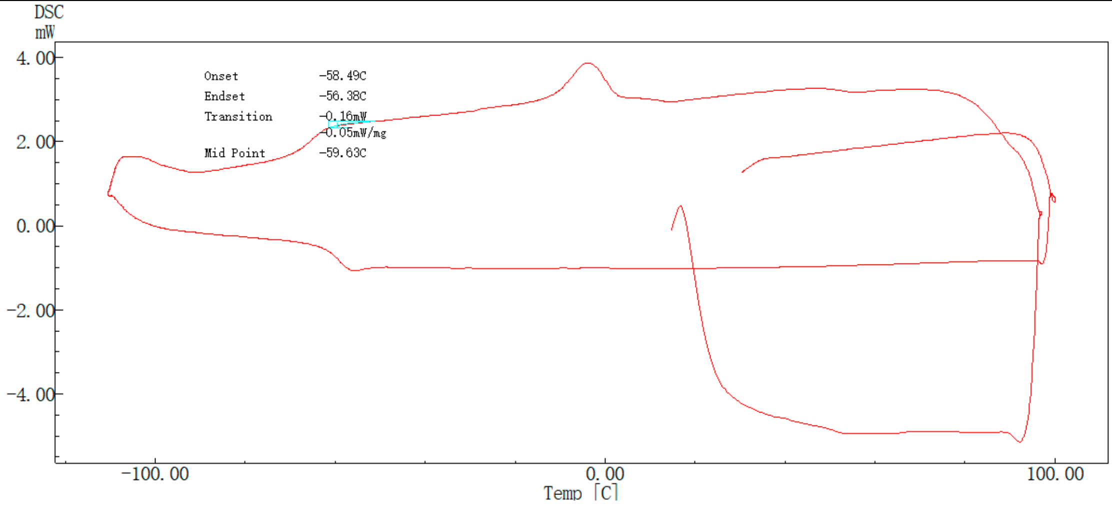

Figure S92. DSC charts of the PMYs by the **3**/Al^{i}Bu$_3$/[Ph$_3$C][B(C$_6$F$_5$)$_4$] systems in Table 3, entry 1.

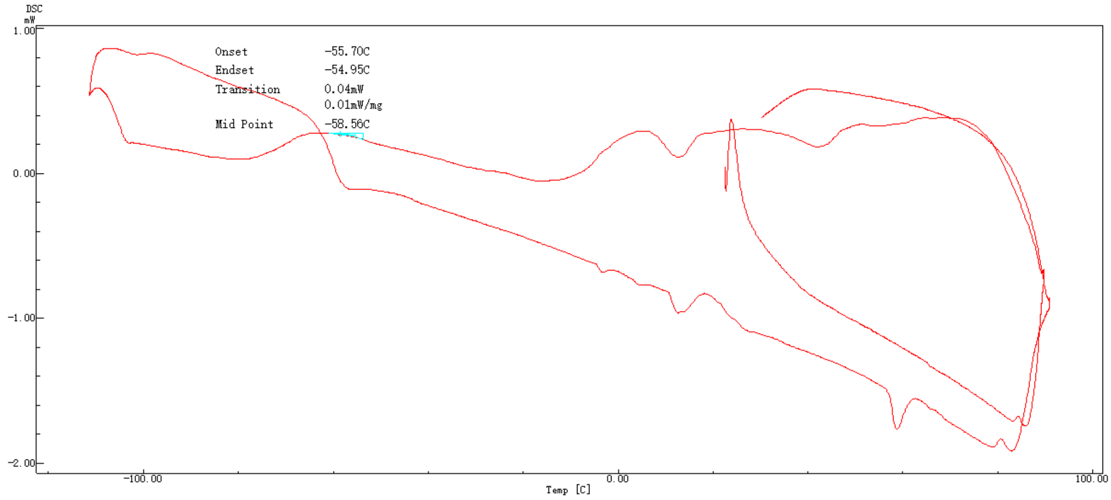

Figure S93. DSC charts of the PMYs by the **3**/Al^{i}Bu$_3$/[PhNHMe$_2$][B(C$_6$F$_5$)$_4$] systems in Table 3, entry 2.

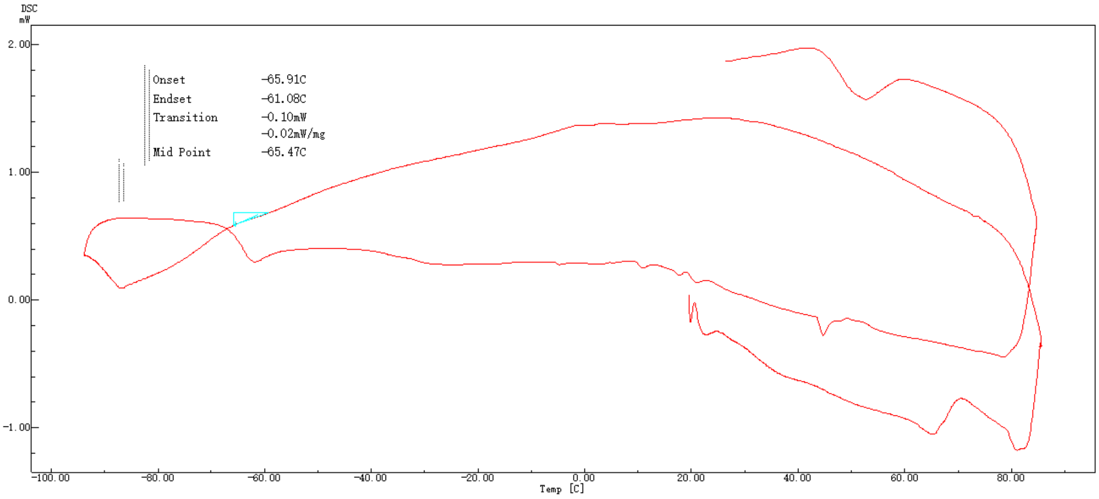

Figure S94. DSC charts of the PMYs by the **3**/Al^{i}Bu$_3$/B(C$_6$F$_5$)$_3$ systems in Table 3, entry 3.

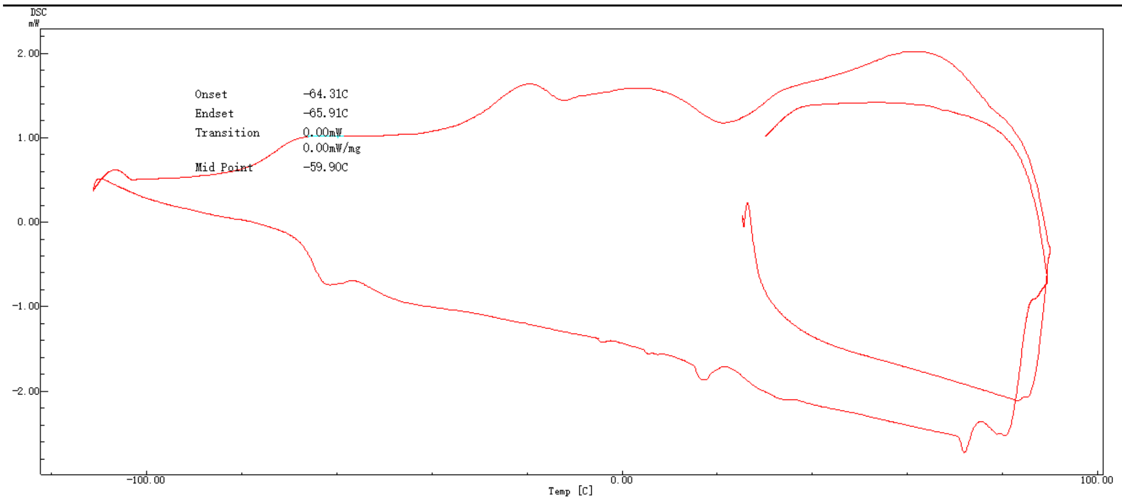

Figure S95. DSC charts of the PMYs by the **1**/Al^{i}Bu$_3$/B(C$_6$F$_5$)$_3$ systems in Table 3, entry 4.

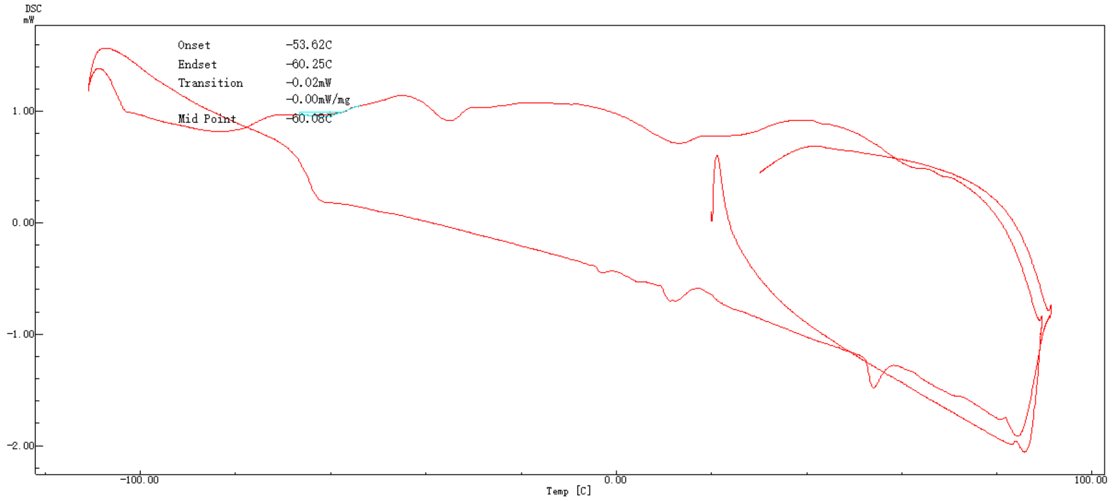

Figure S96. DSC charts of the PMYs by the **2**/Al^{i}Bu$_3$/B(C$_6$F$_5$)$_3$ systems in Table 3, entry 5.

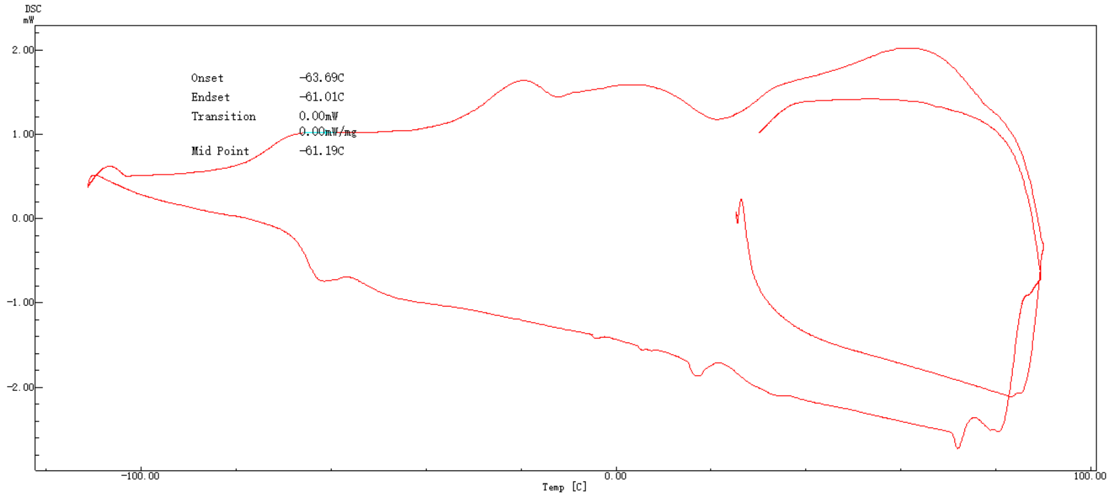

Figure S97. DSC charts of the PMYs by the **3**/Al^{i}Bu$_3$/B(C$_6$F$_5$)$_3$ systems in Table 3, entry 6.

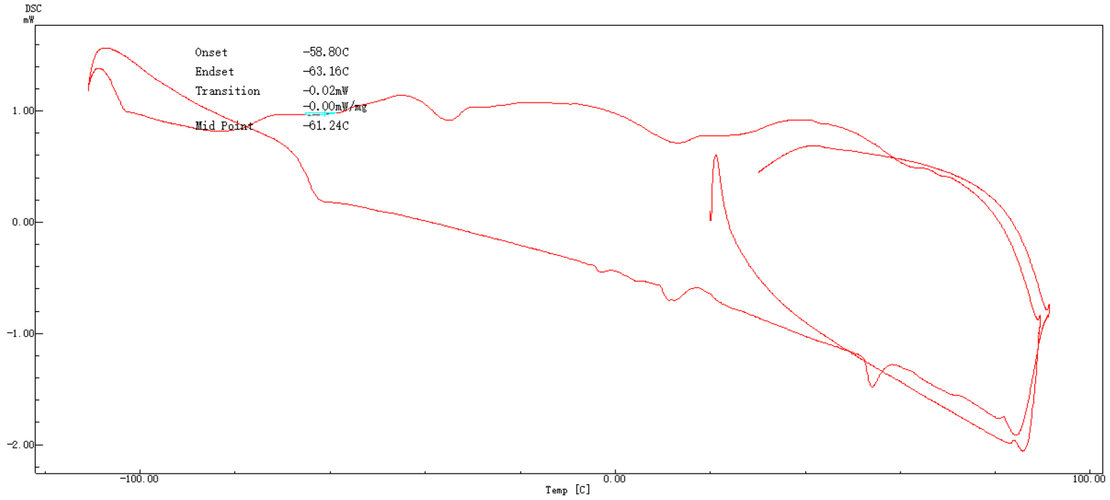

Figure S98. DSC charts of the PMYs by the **3**/Al^{i}Bu$_3$/B(C_6F_5)$_3$ systems in Table 3, entry 7.

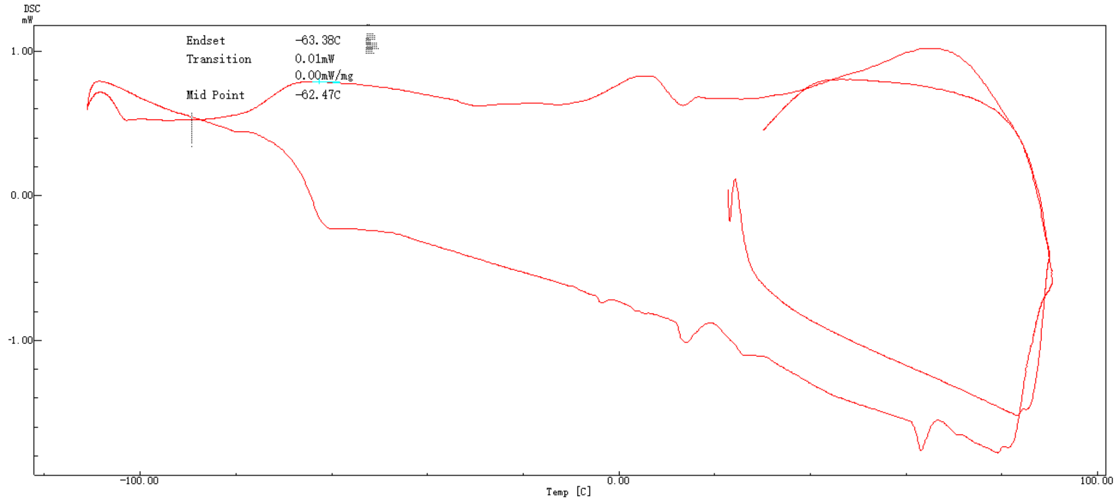

Figure S99. DSC charts of the PMYs by the **3**/Al^{i}Bu$_3$/B(C_6F_5)$_3$ systems in Table 3, entry 8.

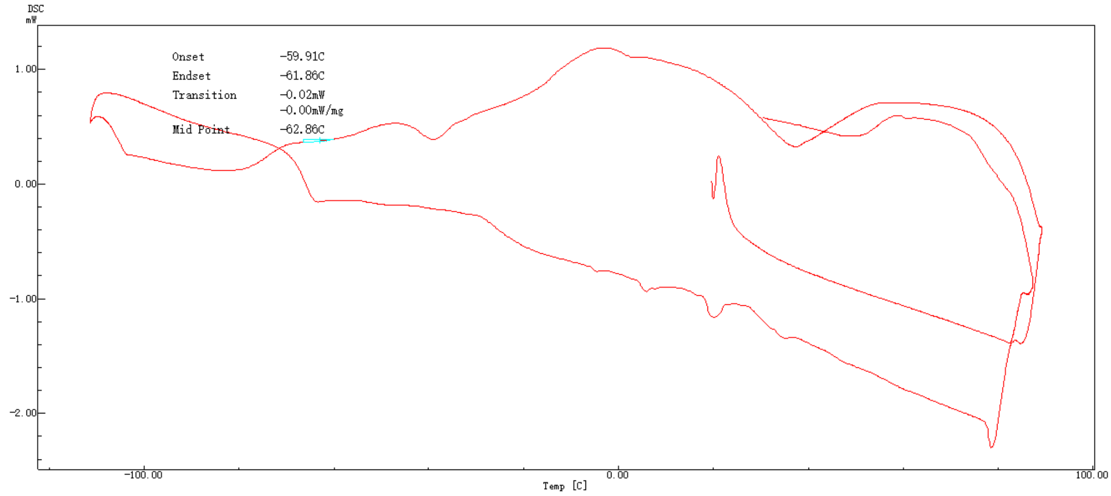

Figure S100. DSC charts of the PMYs by the **3**/Al^{i}Bu$_3$/B(C_6F_5)$_3$systems in Table 3, entry 9.

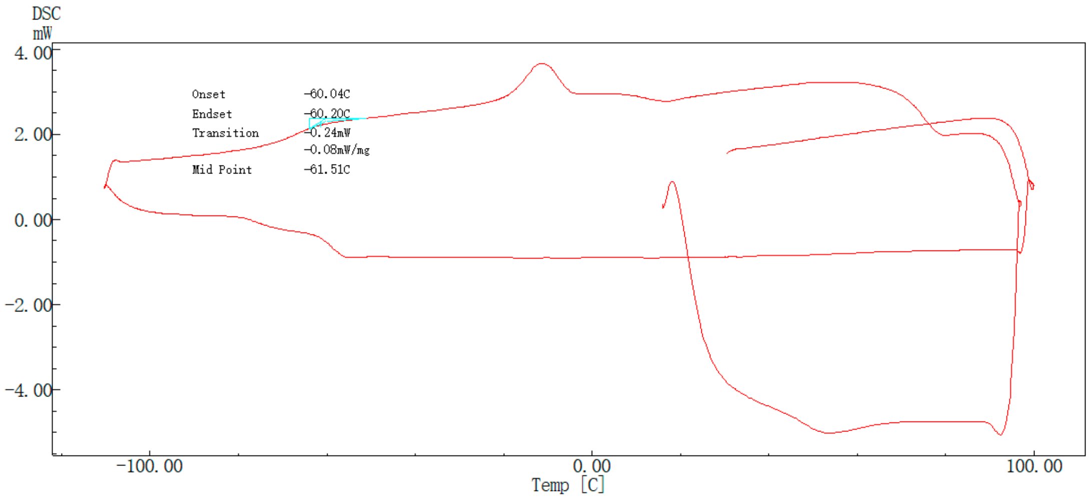

Figure S101. DSC charts of the PMYs by the **3**/Al^{i}Bu$_3$/ B(C_6F_5)$_3$ systems in Table 3, entry 10.

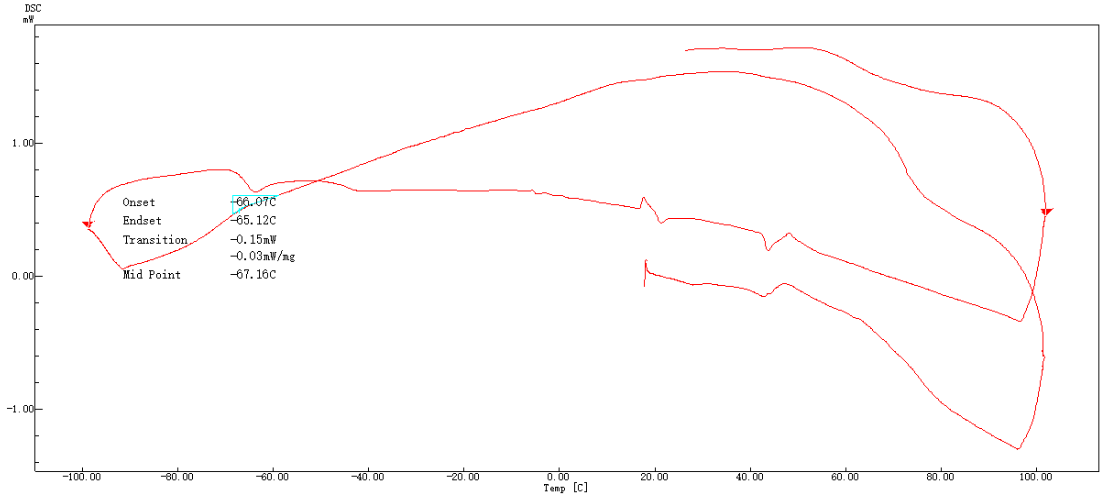

Figure S102. DSC charts of the PMYs by the **3**/Al^{i}Bu$_3$/B(C_6F_5)$_3$ systems in Table 3, entry 11.

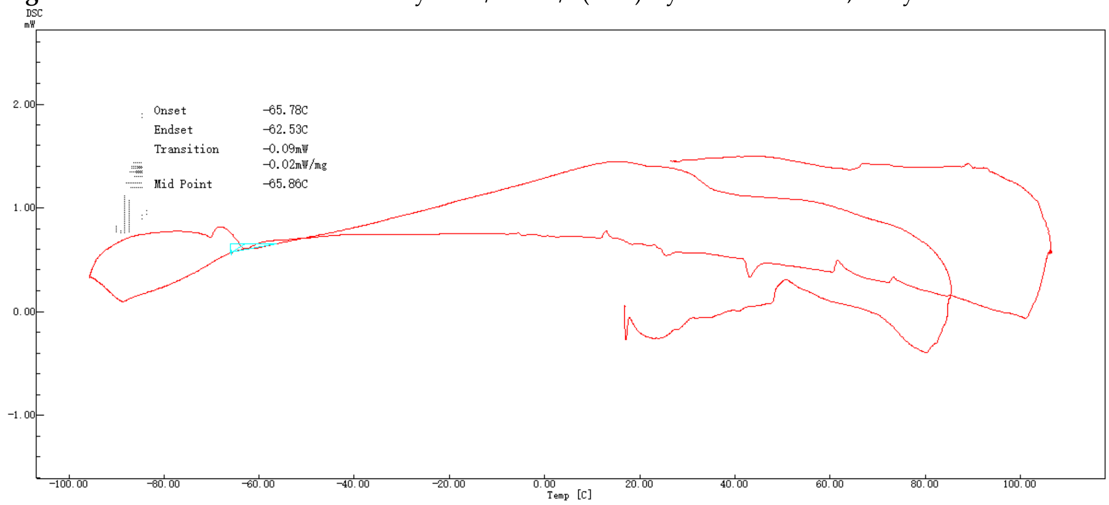

Figure S103. DSC charts of the PMYs by the **3**/Al^{i}Bu$_3$/B(C_6F_5)$_3$ systems in Table 3, entry 12.

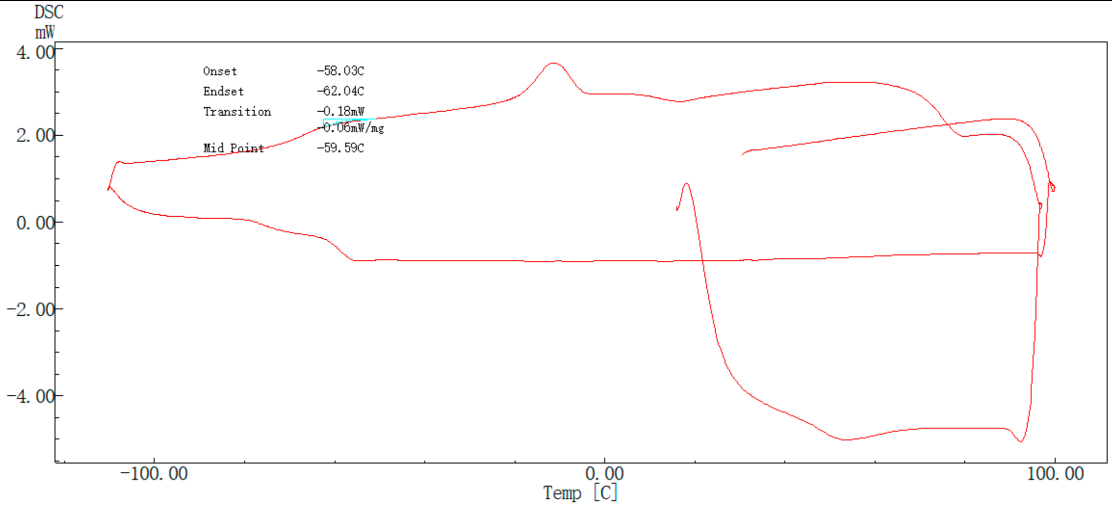

Figure S104. DSC charts of the PMYs by the **3**/Al^{i}Bu$_3$/B(C$_6$F$_5$)$_3$ systems in Table 3, entry 13.

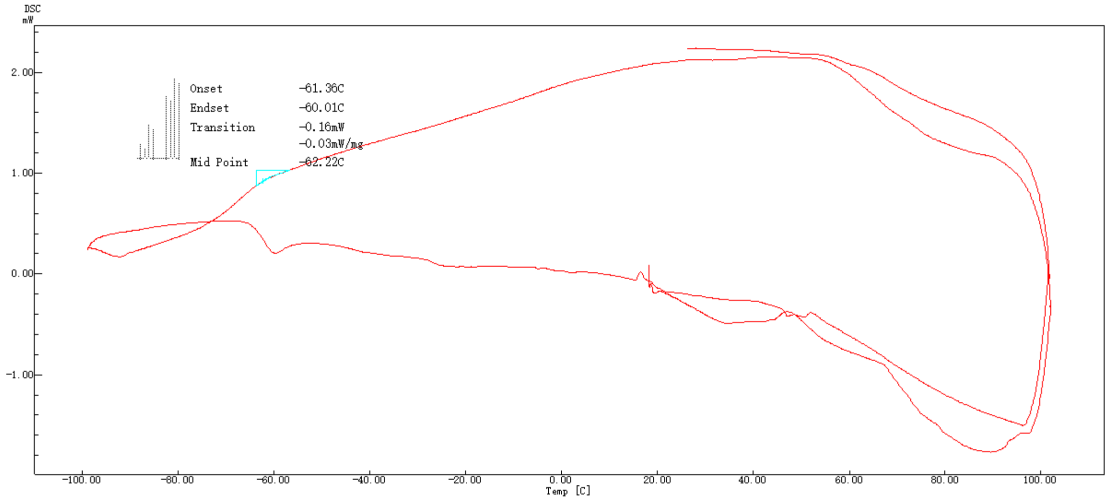

Figure S105. DSC charts of the PMYs by the **3**/Al^{i}Bu$_3$/ B(C$_6$F$_5$)$_3$ systems in Table 3, entry 14.

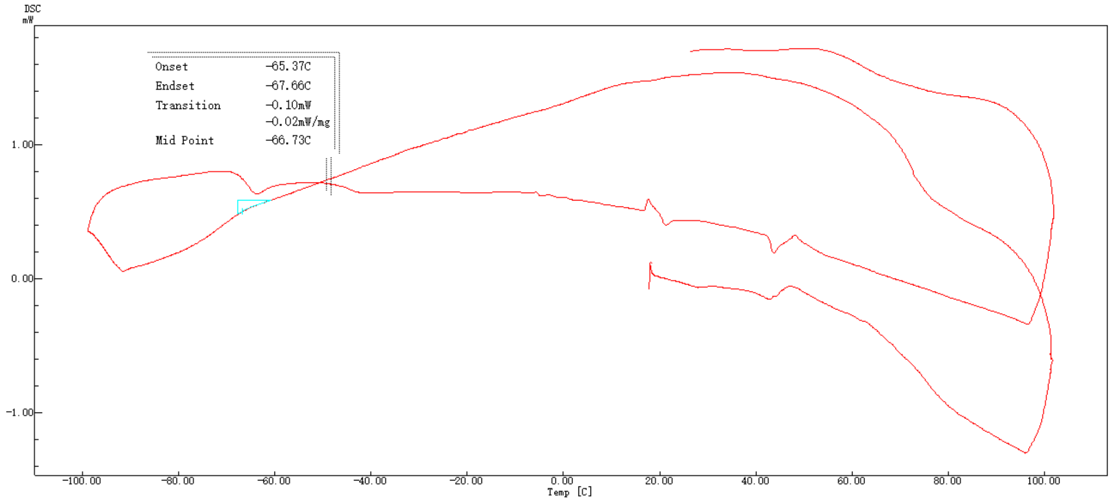

Figure S106. DSC charts of the PMYs by the **3**/Al^{i}Bu$_3$/B(C$_6$F$_5$)$_3$ systems in Table 3, entry 15.

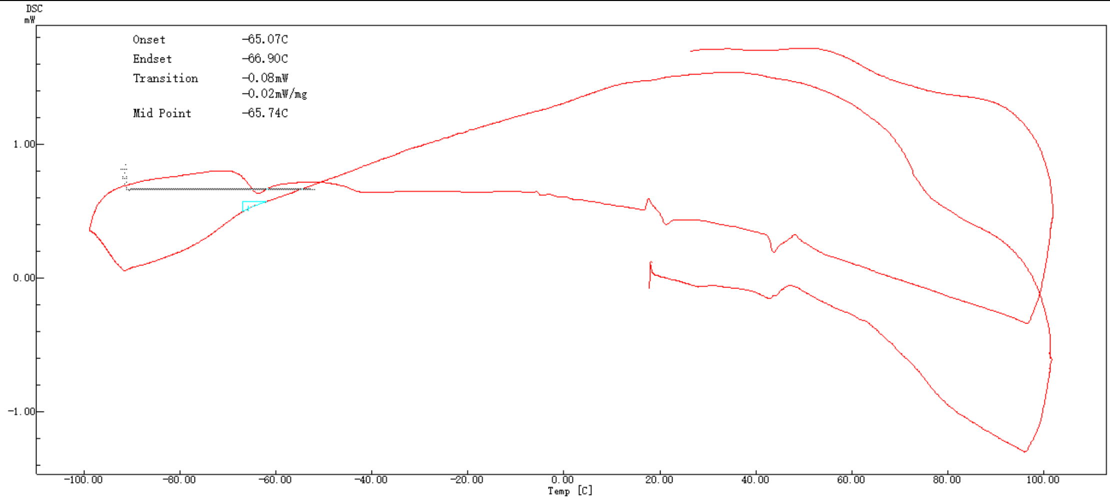

Figure S107. DSC charts of the PMYs by the **3**/Al^{i}Bu$_3$/B(C_6F_5)$_3$ systems in Table 3, entry 16.

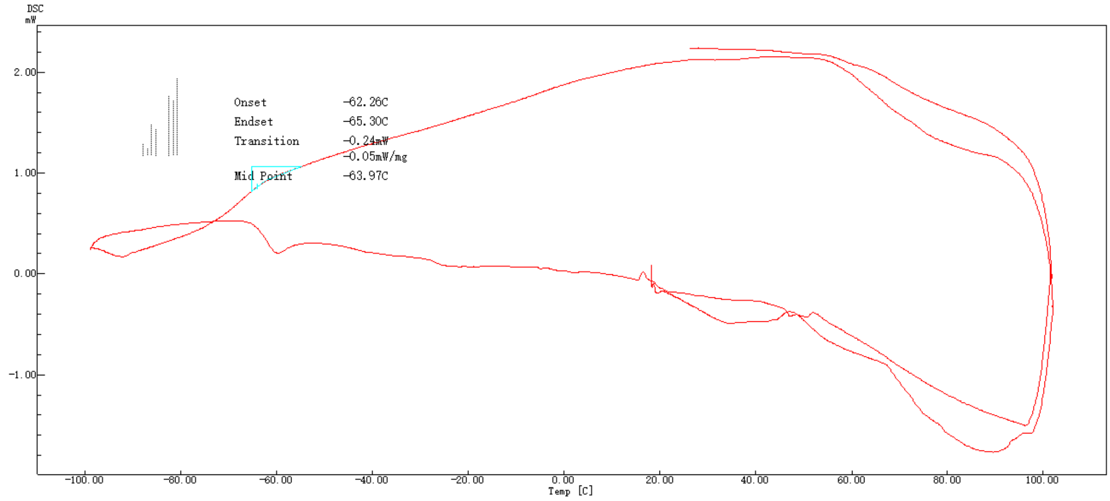

Figure S108. DSC charts of the PMYs by the **3**/Al^{i}Bu$_3$/ B(C_6F_5)$_3$ systems in Table 3, entry 17.

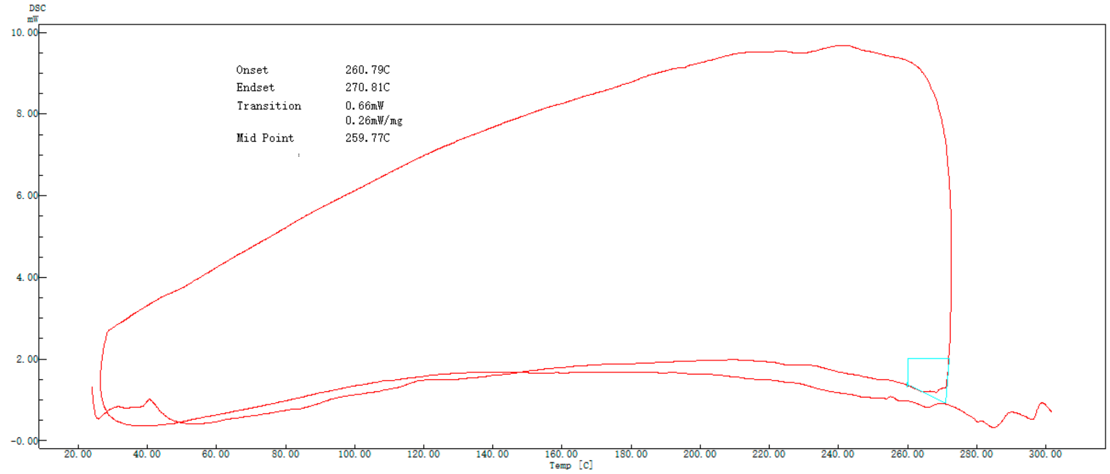

Figure S109. DSC charts of the PSTs by the **1**/Al^{i}Bu$_3$/[Ph$_3$C][B(C_6F_5)$_4$] systems in Table 4, entry 4.

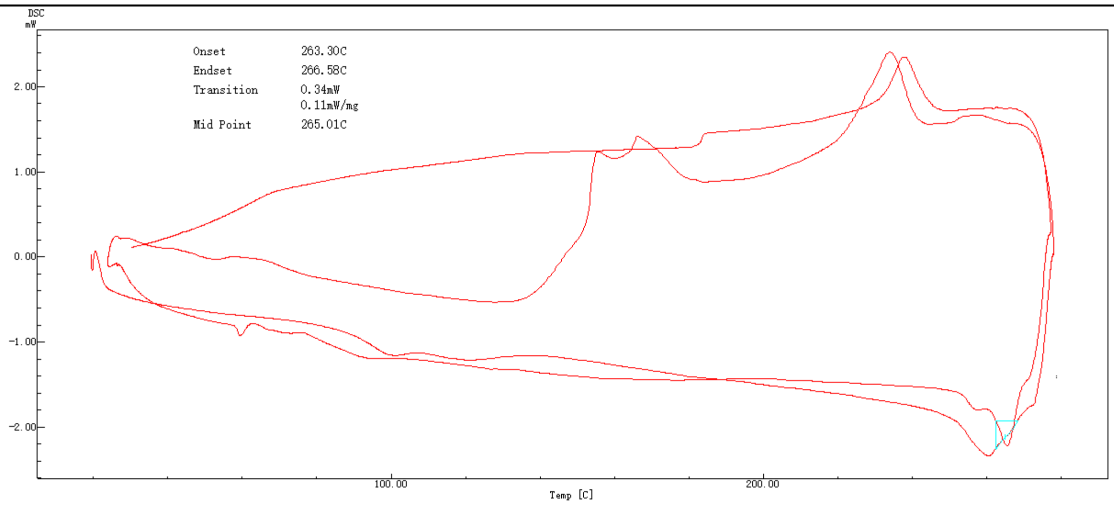

Figure S110. DSC charts of the PSTs by the **1**/Al^{i}Bu$_3$/[Ph$_3$C][B(C$_6$F$_5$)$_4$] systems in Table 4, entry 7.

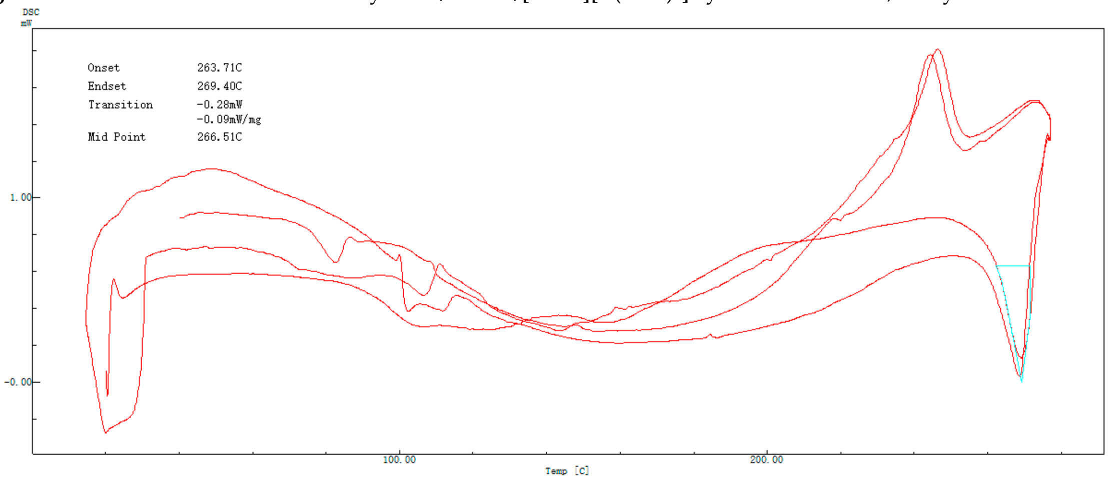

Figure S111. DSC charts of the PSTs by the **1**/Al^{i}Bu$_3$/[PhNHMe$_2$][B(C$_6$F$_5$)$_4$] systems in Table 4, entry 8.

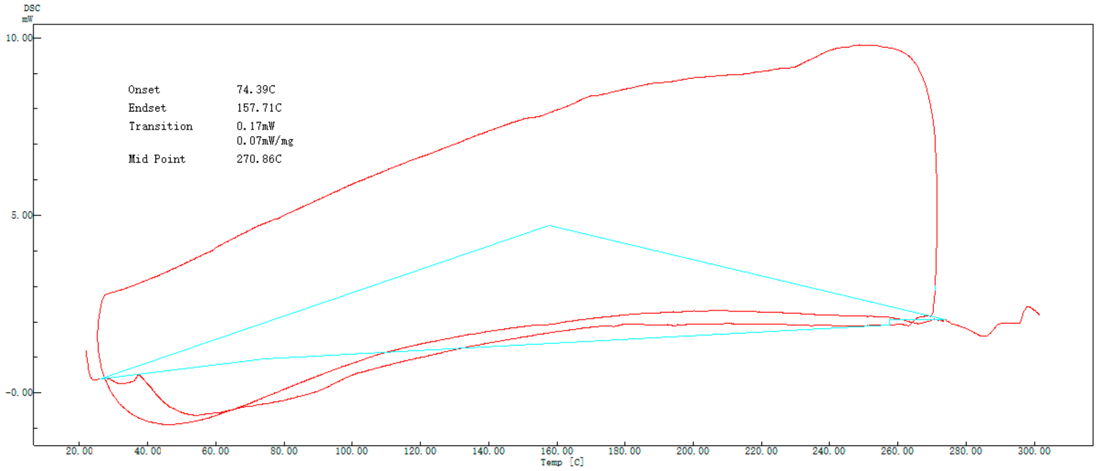

Figure S112. DSC charts of the PSTs by the **1**/Al^{i}Bu$_3$/[PhNHMe$_2$][B(C$_6$F$_5$)$_4$] systems in Table 4, entry 12.

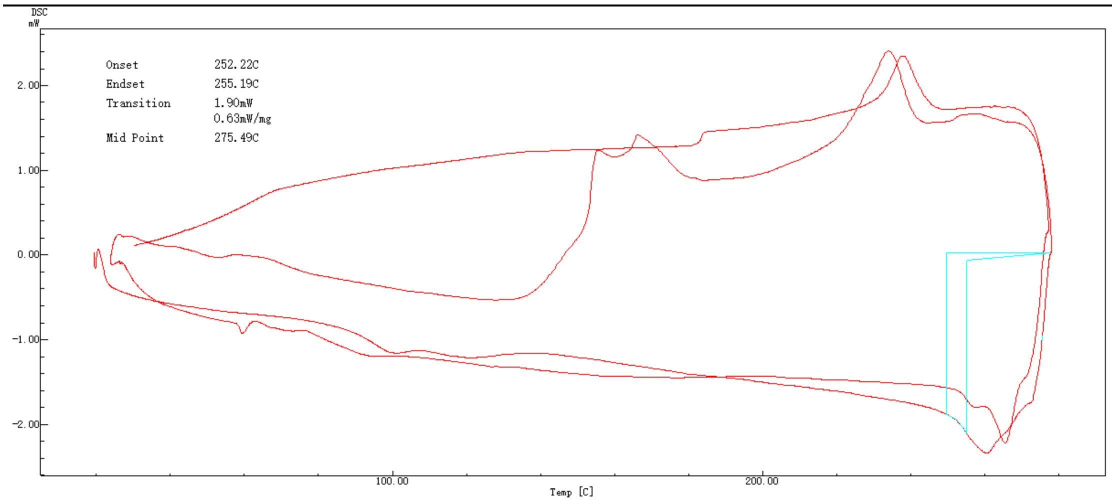

Figure S113. DSC charts of the PSTs by the **1**/Al^{i}Bu$_3$/[PhNHMe$_2$][B(C$_6$F$_5$)$_4$] systems in Table 4, entry 16.

Table S1. X-ray diffraction experimental details for complexes **1**, **2** and **3**.

	**1**	**2**	**3**
Formula	$C_{31}H_{50}NO_2ScSi_2$	$C_{31}H_{50}NO_2LuSi_2$	$C_{31}H_{50}NO_2YSi_2$
M_w	569.86	699.87	613.81
Temperature [K]	296(2) K	296(2) K	296(2) K
Crystal system	Orthorhombic	Orthorhombic	Orthorhombic
Space group	$P2_12_12_1$	$P2_12_12_1$	$P2_12_12_1$
a [Å]	12.1244(3)	12.1763(6)	12.2091(19)
b [Å]	15.5457(4)	15.6753(8)	15.729(3)
c [Å]	19.9964(5)	20.1058(10)	20.211(3)
α [°]	90	90	90
β [°]	90	90	90
γ [°]	90	90	90
Volume [Å^3]	3768.97(16)	3837.5(3)	3881.3(11)
Z	4	4	4
Calc. ϱ [mg/m^3]	1.004	1.211	1.050
μ [mm^{-1}]	0.281	2.657	1.586
Crystal size [mm^3]	0.30 × 0.15 × 0.10	0.30 × 0.20 × 0.18	0.30 × 0.15 × 0.10
θ range [°]	1.964 to 25.050	1.647 to 25.044	1.64 to 28.05
GOF	1.068	1.079	0.981
R1, wR2 [I>2σ (I)]	0.0362/0.0913	0.0169/0.0420	0.0368/0.0911
R indexes (all data)	0.0396/0.0928	0.0180/0.0425	0.0445/0,0935
Largest diff. peak/hole/e Å^3	0.290/-0.194	0.381/-0.283	0.646/-0.402
Data/restraints/params	6681/1/334	6789/3/334	9337/2/334

Crystal Data

Bond length of Complex 1

Atom	Atom	Length/Å	Atom	Atom	Length/Å
Sc1	N1	2.065(3)	C9	C10	1.380(5)
Sc1	O1	2.252(2)	C9	C8	1.399(5)
Sc1	O2	2.161(2)	C8	C13	1.429(5)
Sc1	C1	2.395(3)	C8	C7	1.454(5)
Sc1	C2	2.730(3)	C7	C6	1.389(5)
Sc1	C3	3.067(3)	C7	C2	1.426(5)
Sc1	C20	2.226(4)	C11	C10	1.404(5)
Si1	N1	1.718(3)	C3	C4	1.379(5)
Si1	C1	1.864(3)	C3	C2	1.403(5)
Si1	C14	1.890(4)	C2	C1	1.470(5)

Si1	C15	1.886(4)	C13	C1	1.450(5)
Si2	C20	1.849(4)	C4	C5	1.395(6)
Si2	C21	1.872(4)	C27	C26	1.510(5)
Si2	C22	1.875(4)	C24	C25	1.503(5)
Si2	C23	1.889(4)	C5	C6	1.384(6)
O2	C28	1.459(4)	C25	C26	1.536(5)
O2	C31	1.461(4)	C16	C17	1.531(5)
O1	C27	1.450(4)	C16	C18	1.544(5)
O1	C24	1.468(4)	C16	C19	1.551(6)
N1	C16	1.480(4)	C30	C29	1.518(6)
C12	C11	1.383(5)	C30	C31	1.528(5)
C12	C13	1.403(5)	C29	C28	1.520(5)

Bond length of Complex 2

Atom	Atom	Length/Å	Atom	Atom	Length/Å
Lu1	N1	2.180(3)	C9	C10	1.379(7)
Lu1	O1	2.338(3)	C9	C8	1.400(6)
Lu1	O2	2.255(3)	C8	C13	1.422(6)
Lu1	C1	2.504(4)	C8	C7	1.435(7)
Lu1	C2	2.746(4)	C7	C6	1.412(6)
Lu1	C3	3.044(4)	C7	C2	1.445(6)
Lu1	C20	2.338(4)	C11	C10	1.403(7)
Si1	N1	1.717(4)	C3	C4	1.380(6)
Si1	C1	1.873(4)	C3	C2	1.398(6)
Si1	C14	1.876(5)	C2	C1	1.462(6)
Si1	C15	1.896(5)	C13	C1	1.450(6)
Si2	C20	1.839(4)	C4	C5	1.396(7)
Si2	C21	1.878(5)	C27	C26	1.514(6)
Si2	C22	1.900(6)	C24	C25	1.512(6)
Si2	C23	1.904(6)	C5	C6	1.364(7)
O2	C28	1.460(5)	C25	C26	1.532(7)
O2	C31	1.457(5)	C16	C17	1.548(7)
O1	C27	1.461(5)	C16	C18	1.538(7)
O1	C24	1.447(5)	C16	C19	1.524(7)
N1	C16	1.487(5)	C30	C29	1.479(7)
C12	C11	1.388(6)	C30	C31	1.523(7)
C12	C13	1.401(6)	C29	C28	1.516(6)

Bond length of Complex 3

Atom	Atom	Length/Å	Atom	Atom	Length/Å
Y1	N1	2.213(3)	C2	C1	1.464(5)
Y1	O1	2.378(2)	C9	C8	1.397(5)
Y1	O2	2.310(2)	C9	C10	1.371(6)
Y1	C1	2.566(3)	C8	C7	1.434(6)
Y1	C2	2.769(3)	C7	C6	1.402(6)
Y1	C3	3.036(4)	C8	C13	1.432(5)
Y1	C20	2.392(4)	C11	C12	1.378(5)
Si1	N1	1.719(3)	C3	C4	1.385(6)
Si1	C1	1.871(3)	C12	C13	1.402(5)
Si1	C14	1.884(4)	C13	C1	1.456(5)
Si1	C15	1.877(4)	C24	C25	1.532(6)
Si2	C20	1.835(4)	C25	C26	1.507(6)
Si2	C21	1.878(5)	C10	C11	1.400(6)
Si2	C22	1.889(5)	C26	C27	1.495(5)
Si2	C23	1.873(5)	C4	C5	1.414(7)
O2	C28	1.446(5)	C6	C5	1.368(7)

O2	C31	1.454(4))	C16	C17	1.527(6)
O1	C27	1.473(4)	C16	C18	1.532(7)
O1	C24	1.464(4)	C16	C19	1.518(6)
N1	C16	1.474(5)	C30	C29	1.512(7)
C2	C3	1.403(6)	C30	C31	1.513(6)
C2	C7	1.428(5)	C29	C28	1.517(6)

Bond Angle of Complex 1

Atom	Atom	Atom	Angle/。	Atom	Atom	Atom	Angle/。
N1	Sc1	O2	93.35(10)	Si1	N1	Sc1	100.18(13)
N1	Sc1	C20	109.34(13)	O1	C27	C26	106.3(3)
N1	Sc1	O1	160.04(10)	O1	C24	C25	104.9(3)
N1	Sc1	C1	75.05(11)	C29	C30	C31	103.6(3)
C20	Sc1	O1	90.50(11)	Si1	C1	Sc1	85.25(12)
C3	Sc1	O2	171.23(9)	N1	C16	C17	112.1(3)
O1	Sc1	C2	82.28(10)	N1	C16	C19	110.7(3)
O1	Sc1	C1	90.11(10)	N1	C16	C18	109.7(3)
C20	Sc1	C1	144.95(13)	C6	C5	C4	119.5(3)
O2	Sc1	C20	98.64(11)	C17	C16	C19	106.6(3)
N1	Sc1	C2	91.58(11)	C17	C16	C18	108.8(3)
O2	Sc1	O1	81.08(9)	C19	C16	C18	108.8(3)
C20	Sc1	C2	113.15(12)	C2	C7	C8	107.4(3)
O2	Sc1	C1	116.05(11)	C3	C2	Sc1	89.9(2)
C1	Sc1	C2	32.52(11)	C7	C2	Sc1	120.5(2)
N1	Sc1	C3	86.93(11)	C1	C2	Sc1	61.12(17)
O2	Sc1	C2	144.07(10)	C12	C13	C8	118.4(3)
C20	Sc1	C3	89.52(11)	C4	C3	Sc1	133.9(2)
O1	Sc1	C3	95.71(9)	C3	C2	C7	118.6(3)
C1	Sc1	C3	55.57(11)	C3	C2	C1	131.0(3)
C2	Sc1	C3	27.22(10)	C7	C2	C1	110.2(3)
C16	N1	Sc1	134.6(2)	C12	C13	C1	130.8(3)
C20	Si2	C21	111.55(17)	C8	C13	C1	110.8(3)
C21	Si2	C22	107.54(19)	C4	C3	C2	119.7(3)
C20	Si2	C23	110.54(18)	C3	C4	C5	121.6(4)
C21	Si2	C23	107.44(18)	C2	C3	Sc1	62.87(18)
C22	Si2	C23	106.4(2)	C13	C1	Si1	131.1(3)
C28	O2	C31	110.1(2)	C6	C7	C8	132.3(3)
C24	O1	C27	108.9(2)	C5	C6	C7	120.2(3)
C27	O1	Sc1	130.1(2)	C6	C7	C2	120.3(3)
C24	O1	Sc1	120.95(19)	C9	C8	C7	131.8(3)
C16	N1	Si1	125.1(2)	C8	C9	C10	119.7(3)
C28	O2	Sc1	125.33(19)	C11	C12	C13	119.7(3)
C31	O2	Sc1	124.01(19)	C9	C8	C13	120.9(3)
Si2	C20	Sc1	141.29(19)	C13	C8	C7	107.3(3)
C2	C1	Sc1	86.37(19)	C12	C11	C10	121.7(3)
N1	Si1	C1	99.16(14)	C24	C25	C26	101.4(3)
N1	Si1	C15	117.16(16)	C27	C26	C25	103.4(3)
C1	Si1	C15	110.89(17)	C9	C10	C11	119.7(3)
N1	Si1	C14	115.11(16)	C13	C1	C2	104.3(3)
C1	Si1	C14	110.10(17)	C2	C1	Si1	121.6(3)
C14	Si1	C15	104.44(17)	C13	C1	Sc1	115.6(2)
O2	C31	C30	105.0(3)	C30	C29	C28	102.4(3)
O2	C28	C29	105.4(3)	C20	Si2	C22	113.07(17)

Bond Angle of Complex 2

Atom	Atom	Atom	Angle/。	Atom	Atom	Atom	Angle/。

N1	Lu1	O2	92.53(12)	Si1	N1	Lu1	101.25(16)
N1	Lu1	C20	111.36(14)	O1	C27	C26	105.1(4)
N1	Lu1	O1	156.89(12)	O1	C24	C25	104.9(3)
N1	Lu1	C1	71.80(13)	C29	C30	C31	102.8(4)
C20	Lu1	O1	91.40(13)	Si1	C1	Lu1	86.14(15)
C3	Lu1	O2	169.22(11)	N1	C16	C17	108.7(4)
O1	Lu1	C2	84.14(11)	N1	C16	C19	111.6(4)
O1	Lu1	C1	91.14(12)	N1	C16	C18	110.4(4)
C20	Lu1	C1	148.23(15)	C6	C5	C4	121.6(4)
O2	Lu1	C20	97.40(13)	C17	C16	C19	107.3(4)
N1	Lu1	C2	88.71(12)	C17	C16	C18	110.0(5)
O2	Lu1	O1	80.14(11)	C19	C16	C18	108.8(4)
C20	Lu1	C2	117.13(14)	C2	C7	C8	107.3(4)
O2	Lu1	C1	114.23(13)	C3	C2	Lu1	88.3(3)
C1	Lu1	C2	31.92(13)	C7	C2	Lu1	117.9(3)
N1	Lu1	C3	85.21(13)	C1	C2	Lu1	64.9(2)
O2	Lu1	C2	142.30(12)	C12	C13	C8	118.8(4)
C20	Lu1	C3	93.24(14)	C4	C3	Lu1	130.2(3)
O1	Lu1	C3	97.89(11)	C3	C2	C7	118.6(4)
C1	Lu1	C3	55.06(13)	C3	C2	C1	131.6(4)
C2	Lu1	C3	27.32(12)	C7	C2	C1	109.6(4)
C16	N1	Lu1	133.6(3)	C12	C13	C1	130.4(4)
C20	Si2	C21	110.8(2)	C8	C13	C1	110.8(4)
C20	Si2	C22	111.3(2)	C4	C3	C2	120.1(4)
C21	Si2	C22	108.4(3)	C3	C4	C5	120.8(5)
C20	Si2	C23	110.5(2)	C2	C3	Lu1	64.4(2)
C21	Si2	C23	107.4(3)	O2	C28	C29	105.2(3)
C22	Si2	C23	108.3(3)	C6	C7	C8	132.7(4)
C28	O2	C31	109.4(3)	C5	C6	C7	119.0(4)
C24	O1	C27	110.2(3)	C6	C7	C2	119.9(4)
C27	O1	Lu1	130.2(2)	C7	C8	C9	131.2(4)
C24	O1	Lu1	119.7(2)	C8	C9	C10	119.5(4)
C16	N1	Si1	125.0(3)	C11	C12	C13	119.4(4)
C28	O2	Lu1	124.4(2)	C9	C8	C13	120.9(4)
C31	O2	Lu1	125.7(2)	C13	C8	C7	107.8(4)
Si2	C20	Lu1	137.4(2)	C12	C11	C10	121.6(4)
C2	C1	Lu1	83.2(2)	C24	C25	C26	101.5(4)
N1	Si1	C1	100.48(17)	C27	C26	C25	104.1(4)
N1	Si1	C15	116.3(2)	C9	C10	C11	119.8(4)
C1	Si1	C15	110.5(2)	C13	C1	C2	104.4(4)
N1	Si1	C14	115.2(2)	C2	C1	Si1	121.6(3)
C1	Si1	C14	109.5(2)	C13	C1	Si1	132.1(3)
C14	Si1	C15	104.8(2)	C30	C29	C28	104.5(4)
O2	C31	C30	104.7(4)	C13	C1	Lu1	113.4(3)

Bond Angle of Complex 3

Atom	Atom	Atom	Angle/。	Atom	Atom	Atom	Angle/。
N1	Y1	O2	92.56(10)	Si1	N1	Y1	101.86(14)
N1	Y1	C20	112.29(13)	O1	C27	C26	104.2(3)
N1	Y1	O1	155.92(11)	O1	C24	C25	105.3(3)
N1	Y1	C1	70.57(11)	C29	C30	C31	104.2(4)
C20	Y1	O1	91.35(12)	Si1	C1	Y1	86.00(12)
C3	Y1	O2	168.51(10)	N1	C16	C17	112.9(4)
O1	Y1	C2	84.65(10)	N1	C16	C19	109.4(3)
O1	Y1	C1	91.72(10)	N1	C16	C18	110.6(4)
C20	Y1	C1	149.02(13)	C6	C5	C4	120.6(4)
O2	Y1	C20	96.98(12)	C17	C16	C19	109.3(4)

N1	Y1	C2	87.82(11)	C17	C16	C18	107.1(4)
O2	Y1	O1	79.54(9)	C19	C16	C18	107.4(4)
C20	Y1	C2	118.36(13)	C2	C7	C8	108.1(3)
O2	Y1	C1	113.89(10)	C3	C2	Y1	86.9(2)
C1	Y1	C2	31.58(11)	C7	C2	Y1	116.9(2)
N1	Y1	C3	84.56(11)	C1	C2	Y1	66.55(18)
O2	Y1	C2	141.54(10)	C12	C13	C8	118.5(3)
C20	Y1	C3	94.39(12)	C4	C3	Y1	129.2(3)
O1	Y1	C3	98.66(10)	C3	C2	C7	119.3(4)
C1	Y1	C3	54.68(11)	C3	C2	C1	130.7(3)
C2	Y1	C3	27.47(11)	C7	C2	C1	109.8(3)
C16	N1	Y1	132.7(3)	C12	C13	C1	131.3(3)
C20	Si2	C21	111.5(2)	C8	C13	C1	110.2(3)
C20	Si2	C22	111.7(2)	C4	C3	C2	119.9(4)
C21	Si2	C22	107.8(3)	C3	C4	C5	120.3(4)
C20	Si2	C23	111.4(2)	C2	C3	Y1	65.6(2)
C21	Si2	C23	107.9(2)	O2	C28	C29	105.4(4)
C22	Si2	C23	106.3(2)	C6	C7	C8	132.3(4)
C28	O2	C31	110.0(3)	C5	C6	C7	120.2(4)
C24	O1	C27	109.5(3)	C6	C7	C2	119.6(4)
C27	O1	Y1	119.6(2)	C7	C8	C9	132.3(4)
C24	O1	Y1	130.9(2)	C8	C9	C10	120.2(4)
C16	N1	Si1	125.3(3)	C11	C12	C13	119.9(4)
C28	O2	Y1	125.1(2)	C9	C8	C13	120.1(4)
C31	O2	Y1	124.4(2)	C13	C8	C7	107.5(3)
Si2	C20	Y1	136.4(2)	C12	C11	C10	121.3(4)
C2	C1	Y1	81.9(2)	C24	C25	C26	103.1(3)
N1	Si1	C1	101.17(15)	C27	C26	C25	103.7(3)
N1	Si1	C15	116.64(19)	C9	C10	C11	120.0(4)
C1	Si1	C15	110.53(18)	C13	C1	C2	104.5(3)
N1	Si1	C14	115.46(19)	C2	C1	Si1	122.7(3)
C1	Si1	C14	108.78(18)	C13	C1	Si1	131.8(3)
C14	Si1	C15	104.2(2)	C30	C29	C28	102.2(4)
O2	C31	C30	105.5(3)	C13	C1	Y1	111.9(2)

References

1. Okuda, J.; Schattenmann, F.J.; Wocadlo, S.; Massa, W. Synthesis and Characterization of Zirconium Complexes Containing a Linked Amido-Fluorenyl Ligand. Organometallics 1995, 14, 789–795.
